# COVID-19 vaccine hesitancy in the UK: the Oxford coronavirus explanations, attitudes, and narratives survey (Oceans) II

**DOI:** 10.1017/S0033291720005188

**Published:** 2020-12-11

**Authors:** Daniel Freeman, Bao S. Loe, Andrew Chadwick, Cristian Vaccari, Felicity Waite, Laina Rosebrock, Lucy Jenner, Ariane Petit, Stephan Lewandowsky, Samantha Vanderslott, Stefania Innocenti, Michael Larkin, Alberto Giubilini, Ly-Mee Yu, Helen McShane, Andrew J. Pollard, Sinéad Lambe

**Affiliations:** 1 Department of Psychiatry, University of Oxford, Oxford, UK; 2 Oxford Health NHS Foundation Trust, Oxford, UK; 3 The Psychometrics Centre, University of Cambridge, Cambridge, UK; 4 Online Civic Culture Centre, Department of Communication and Media, Loughborough University, Loughborough, UK; 5 School of Psychological Science, University of Bristol, Bristol, UK; 6 Oxford Vaccine Group, Department of Paediatrics, University of Oxford, Oxford, UK; 7 Smith School of Enterprise and the Environment, University of Oxford, Oxford, UK; 8 Department of Psychology, Life and Health Sciences, Aston University, Birmingham, UK; 9 Oxford Uehiro Centre for Practical Ethics, University of Oxford, Oxford, UK; 10 Nuffield Department of Primary Care, University of Oxford, Oxford, UK; 11 Nuffield Department of Medicine, The Jenner Institute, University of Oxford, Oxford, UK

**Keywords:** Covid-19 vaccine hesitancy, vaccine confidence, mistrust, conspiracy beliefs

## Abstract

**Background:**

Our aim was to estimate provisional willingness to receive a coronavirus 2019 (COVID-19) vaccine, identify predictive socio-demographic factors, and, principally, determine potential causes in order to guide information provision.

**Methods:**

A non-probability online survey was conducted (24th September−17th October 2020) with 5,114 UK adults, quota sampled to match the population for age, gender, ethnicity, income, and region. The Oxford COVID-19 vaccine hesitancy scale assessed intent to take an approved vaccine. Structural equation modelling estimated explanatory factor relationships.

**Results:**

71.7% (*n*=3,667) were willing to be vaccinated, 16.6% (*n*=849) were very unsure, and 11.7% (*n*=598) were strongly hesitant. An excellent model fit (RMSEA=0.05/CFI=0.97/TLI=0.97), explaining 86% of variance in hesitancy, was provided by beliefs about the collective importance, efficacy, side-effects, and speed of development of a COVID-19 vaccine. A second model, with reasonable fit (RMSEA=0.03/CFI=0.93/TLI=0.92), explaining 32% of variance, highlighted two higher-order explanatory factors: ‘excessive mistrust’ (*r*=0.51), including conspiracy beliefs, negative views of doctors, and need for chaos, and ‘positive healthcare experiences’ (*r*=−0.48), including supportive doctor interactions and good NHS care. Hesitancy was associated with younger age, female gender, lower income, and ethnicity, but socio-demographic information explained little variance (9.8%). Hesitancy was associated with lower adherence to social distancing guidelines.

**Conclusions:**

COVID-19 vaccine hesitancy is relatively evenly spread across the population. Willingness to take a vaccine is closely bound to recognition of the collective importance. Vaccine public information that highlights prosocial benefits may be especially effective. Factors such as conspiracy beliefs that foster mistrust and erode social cohesion will lower vaccine up-take.

## Introduction

The success of a safe, efficacious coronavirus 2019 (COVID-19) vaccine will depend on uptake; if there are individuals who are reluctant or unwilling to be immunised, uptake will be limited. A low level of intent to be vaccinated can be understood within the concept of hesitancy, the behavioural ‘delay in acceptance or refusal of vaccines despite availability of vaccine services’ (SAGE Working Group on Vaccine Hesitancy, 2014). There is a continuum from acceptance to refusal of all vaccines, with vaccine hesitancy considered to reside between the two poles, and potential variation within individuals in stance on vaccination for different diseases. Vaccine hesitancy can have effects for both the individual (a greater risk of having the disease) and potentially the community (greater virus transmission). In this paper, by assessing expressed willingness to receive an approved vaccine, we wished to estimate in the UK the level of potential COVID-19 vaccine hesitancy (delay or refusal) and identify groups within the population in which it may be higher. Our primary focus was on explaining vaccine hesitancy at an individual psychological level in order to inform strategies to increase acceptance rates. Beliefs, which are potentially amenable to change, are well-established drivers of actions. Therefore, we focussed on identifying a broad cluster of cognitions that may inhibit or facilitate uptake of a COVID-19 vaccine. In essence, we wished to estimate the extent of the potential problem, identify pockets of pronounced hesitancy, and determine the content of key cognitions driving vaccine hesitancy.

In early May 2020, our first Oxford Coronavirus Explanations, Attitudes, and Narratives Survey (OCEANS) was focussed on coronavirus conspiracy beliefs and adherence to government social distancing guidelines (Freeman et al., 2020). When survey respondents were asked whether they would accept a COVID-19 vaccine, 47.5% responded definitely, 22.1% probably, 18.4% possibly, 7.3% probably not, and 4.8% definitely not. Higher levels of vaccine hesitancy were associated with higher levels of coronavirus conspiracy beliefs. A connection between vaccine hesitancy and conspiracy beliefs has been replicated (Bertin et al., 2020), and is consistent with work showing that a conspiracy mentality is associated with rejection of vaccine science (Lewandowsky et al., 2013). In a UK survey conducted in July 2020, 2,237 people were asked how likely they would be to get a coronavirus vaccine (The Policy Institute, 2020); 30% of participants responded that they would be certain to get a vaccine, 23% very likely, 20% fairly likely, 9% not very likely, 3% not at all likely, 4% definitely not, and 11% did not know. These initial studies indicate that approximately 70% of the UK population are likely to accept a COVID-19 vaccine relatively readily, 20% may be ambivalent, and 10% are unlikely to accept a vaccine.

Vaccine hesitancy is typically viewed within the framework of complacency, confidence, and convenience, although it is notable that this is a complex research area in which terms, definitions, and measurement can vary in usage (Bedford et al., 2018; Dudley et al., 2020). At the heart, an individual's decision as to whether to accept a vaccine can be understood as a weighing up of risk and benefit. Vaccine hesitancy is seen when there is a low perception of need for a vaccination (termed complacency) and concerns over the efficacy and safety (termed low confidence) (e.g. Larson et al., 2015; Crouse Quinn et al., 2019; Karlsson et al., 2019; Mesch & Schwirian, 2019). A lack of confidence in a vaccine is exacerbated by misunderstanding of how immunisation works (e.g. Zingg & Siergrist, 2012), distrust of government and healthcare authorities (e.g. Lee et al., 2016), and newness of a vaccine (e.g. Karafillakisa et al., 2019). Added to the balance is consideration of how easy it is to access a vaccine (termed convenience) (e.g. Fournet et al., 2018). Social processes such as norms, altruistic intent, and collective responsibility, have also been highlighted (Brewer et al., 2017; Crouse Quinn, Hilyard et al., 2017; Betsch et al., 2018).

We wished to build on this framework to understand COVID-19 vaccine hesitancy. In relation to a future UK approved COVID-19 vaccine, we aimed to assess a range of specific beliefs about the importance, benefits, and safety (i.e. complacency and confidence). There are specific aspects of the current epidemic that may affect complacency (e.g. inaccurate severity analogies to seasonal influenza) and confidence (e.g. speed of vaccine development). As the UK has universal free at point of access healthcare provision, we did not focus on convenience, although we did include questions concerning access via general practitioner surgeries, pharmacies, and schools, which are the most likely delivery routes. We also added an additional approach to assessing specific beliefs about the benefits and risks of a COVID-19 vaccine: general trust and mistrust. In essence, willingness to take a vaccine is about trust: that the vaccine is needed, that it will work, and that it is safe. Therefore, unwillingness to take a vaccine will be more likely when excessive mistrust is an individual's default position. If an individual is mistrustful of experts, authority, and institutions, the same tendency will apply to attitudes to a vaccination. Distrust will be more likely when individuals (i) generally feel disrespected and vulnerable to exploitation (marginalised), (ii) view doctors as looking down on them, (iii) hold conspiracy beliefs, (iv) hold particular worldviews (e.g. individualism), and (v) are discontented (e.g. express a ‘need for chaos’). Therefore, we conducted a new survey of the UK population, in which we assessed willingness to be vaccinated for COVID-19 (as a marker of vaccine hesitancy) and a wide range of explanatory factors. We hypothesised that vaccine hesitancy would be closely connected to specific confidence and complacency beliefs about a COVID-19 vaccine and also associated with wider makers of mistrust.

## Methods

### Participants

An online survey with a new quota sampled UK participant group of 5,114 adults (18+ years old) was conducted from 24th September to the 17th October 2020 via a market research company. The quotas were based on UK Office for National Statistics population estimate data for gender, age, ethnicity, income, and region. Invited respondents did not know the topic of the survey before provisional agreement to complete it. OCEANSII was approved by the University of Oxford Central University Research Ethics Committee. (See online supplementary materials for further survey detail.)

### Assessments

All of the assessment items included in the survey, the details of the factor analyses, the items that comprise each factor, and Cronbach's alphas can be viewed in the online supplementary materials.

#### Oxford COVID-19 vaccine hesitancy scale

There was no existing scale to assess expressed intent to accept a COVID-19 vaccine. We therefore developed a pool of 15 items (see online supplementary materials), with feedback from public involvement groups including representatives from ethnic minority health groups. Item-specific response options (Saris et al., 2010), coded from 1 to 5, were used. A ‘Don't know’ option was also provided, which was excluded from scoring. Exploratory and confirmatory factor analyses were used to derive a final seven-item scale from the item pool (Figure 3). Higher scores indicate a higher level of vaccine hesitancy.

#### Oxford COVID-19 vaccine confidence and complacency scale

We developed, with public involvement in item generation, 20 items concerning attitudes around vaccine complacency and confidence. Item-specific response options (Saris et al., 2010), coded from 1 to 5, were used. A ‘Don't know’ option was also provided, which was excluded from scoring. Exploratory and confirmatory factor analyses identified four factors: collective importance of a COVID-19 vaccine; beliefs that the respondent may get COVID-19 and the vaccine will work; speed of vaccine development; and side effects. Higher scores indicate a greater degree of negative attitudes.

#### Vaccine hesitancy scale

This scale (Shapiro et al., 2018) was included to test convergent validity with the Oxford COVID-19 vaccine hesitancy scale. The nine-item VHS asks parents about their views of childhood vaccines, and we re-worded the scale where appropriate to make it suitable for all survey respondents. A one-factor solution emerged. Each item was rated on a 1 (strongly disagree) to 5 (strongly agree) scale. Higher scores indicate greater hesitancy.

#### Vaccination knowledge scale

This questionnaire presents individuals with nine statements about vaccines that they are asked to rate as correct, incorrect, or do not know (Zingg & Siergrist, 2012). Factor analysis identified two factors: general knowledge about vaccines and knowledge about childhood vaccines. Incorrect or do not know are scored as zero and correct is scored as one. Therefore, higher scores indicate better knowledge of vaccines.

#### Oxford trust in doctors and developers questionnaire

We developed 11 new items about inter-personal disrespect from doctors and 5 items about mistrust of vaccine developers. Each item was rated on a 1 (disagree completely) to 4 (agree completely) scale, with a ‘don't know’ option that was not scored. Factor analysis indicated three factors: interpersonal disrespect by doctors; respect from doctors and negative views of vaccine developers. Higher scores indicated greater disrespect from doctors, less respect from doctors, and greater negative views of vaccine developers.

#### Attitudes to doctors and medicine questionnaire

Nineteen items about doctors and medicine are rated on a scale from strongly disagree (1) to strongly agree (6) (Marteau, 1990). Confirmatory factor analysis supported a four-factor model: positive attitude to doctors; negative attitude to doctors; positive attitude to medicine; and negative attitude to medicine. Higher scores indicate greater positive attitudes to doctors or medicine and greater negative attitudes to doctors or medicine.

#### The MacArthur scale of subjective social status

Two separate items assess where people view themselves on a social ladder relative to other people in the UK or relative to other people in their community (Adler et al., 2000). Each item is rated 0−10. Higher scores were coded to indicate lower subjective social status.

#### Brief core schema scales

Twelve items assess beliefs about self, rated on a scale from do not believe (0) to believe it totally (4) (Fowler et al., 2006). Confirmatory factor analysis indicated a negative self-factor and a positive self-factor. Higher scores reflect greater endorsement of items.

#### General practice assessment questionnaire-R2

Eight items were used from this questionnaire, assessing how the person has been treated by their GP (Roland et al., 2013). Each item is rated on a scale from very good (1) to very poor (5). Factor analysis indicated a one-factor solution concerning positive GP experiences. Higher scores indicate fewer positive GP experiences.

#### NHS experience questionnaire

We developed eight items assessing positive and negative experiences of the NHS. Each item is rated on a three-point scale, from No (1) to Yes (3). Factor analysis identified two factors: positive NHS experiences; and negative NHS experiences. Higher scores indicate fewer positive NHS experiences, and greater negative NHS experiences.

#### OCEANS coronavirus conspiracy scale

From the first OCEANS study, we developed a seven-item general coronavirus conspiracy scale and a 14-item-specific coronavirus conspiracy scale (Freeman et al., 2020). Each item was rated on a scale from do not agree (1) to agree completely (5). We also added a ‘Don't know’ response option, which was not included in the score. Higher scores indicate greater endorsement of coronavirus conspiracy beliefs. We only used the general conspiracy scale for the explanatory modelling. Confirmatory factor analysis confirmed a single factor.

#### Vaccine conspiracy beliefs scale

This seven-item questionnaire asks participants how much they agree on a seven-point scale with vaccine conspiracy statements (Shapiro et al., 2016). A one-factor CFA model indicated a good fit for the seven items. Higher scores indicate greater endorsement of conspiracy statements.

#### Everyday discrimination scale

Individuals rate on a scale from almost everyday (1) to never (6) how often nine negative experiences happen to them (Williams et al., 1997). Factor analysis indicated two factors: others disrespectful and others react negatively. Higher scores indicate fewer negative experiences.

#### Dimensions of anger reactions-5

Anger is assessed with five items, rated on a scale from 1 (none) to 5 (all of the time) (Forbes et al., 2014). CFA resulted in a single factor with good model fit. Higher scores indicate higher levels of anger.

#### Need for chaos

Eleven items assessing ‘need for chaos’, understood as a desire to bring down the established political order in order to increase one's own social status, are rated on a scale from strongly disagree (1) to strongly agree (7) (Petersen et al., 2020). A one-factor CFA resulted in an excellent fit. Higher scores indicate a higher level of need for chaos.

#### Lifestyle and economic/government liberty

Seven items assessing libertarian worldviews were rated on a scale from strongly disagree (1) to strongly agree (6) (Iyer et al., 2012). Factor analysis identified a one-factor solution. Higher scores indicate greater libertarian beliefs.

#### Populist attitudes

Five items assessed populist views, each rated on a scale from strongly disagree (1) to strongly agree (6) (Akkerman et al., 2014). Factor analysis identified a one-factor model. Higher scores indicate a greater level of populist beliefs.

#### Perceived religious influence on health behaviour and illness as punishment by God for sin

This questionnaire contains two separate scales concerning the influence of religion on an individual's health views (Holt et al., 2009). Each of the 15 items is rated on a scale from strongly disagree (1) to strongly agree (4). Factor analysis identified a religious influence on health behaviour factor and an illness as punishment for sin factor. Higher scores indicate higher influence of religion on health behaviour and greater belief in illness as punishment.

#### Following of UK government coronavirus guidance

Participants were asked to rate how often they followed nine key aspects of government guidance at the time on a five-point scale. Higher scores indicate greater adherence to guidance. Participants were also asked to rate on the same scale two general items assessing how much they follow the guidance. Participants were also asked to rate on a five-point scale how likely it was that they would accept a COVID-19 diagnostic test and a COVID-19 antibody test. Higher scores on these two items indicate less likelihood of accepting the tests.

### Analysis

Structural equation modelling (SEM) was used to assess two explanatory models. The first was between beliefs about a COVID-19 vaccine and hesitancy, and the second was between the broader psychological constructs of mistrust and vaccine hesitancy. SEM comprises a confirmatory measurement model, also known as the confirmation factor analysis (CFA), which estimates the relations among latent constructs and their observed indicators, and a structural model, which estimates the relations among constructs (Kline, 2015). The lavaan R package was used to conduct the SEM analysis (Rosseel, 2012). We utilised the full information maximum likelihood (FIML) estimation procedure with robust (Huber−White) standard errors and a scaled test statistic to estimate the SEM models. In assessing the relations among constructs in the structural model, we employed a backward elimination procedure to reduce multicollinearity issues (Hocking, 1976; Grewal et al., 2004). Full details of the SEM methods can be seen in the online supplementary materials.

## Results

A summary of the socio-demographic characteristics of the participants is provided in Table 1.

**Table 1.** Socio-demographic information

	Mean (SD)/ *n* (%)
Age in years	46.9 (17.1)
Age ranges	
18−24	571 (11.2%)
25−34	898 (17.6%)
35−44	883 (17.3%)
45−54	929 (18.2%)
55−64	761 (14.9%)
65−99	1072 (21.0%)
Gender: male; female; non-binary; prefer not say	2574; 2515; 20; 5
Ethnicity:	
White	
English / Welsh / Scottish / Northern Irish / British	4056 (79.3%)
Irish	57 (1.1%)
Gypsy or Irish Traveller	8 (0.2%)
Any other White background	204 (4.0%)
Mixed / Multiple ethnic groups	
White and Black Caribbean	43 (0.8%)
White and Black African	17 (0.3%)
White and Asian	34 (0.7%)
Any other Mixed / Multiple ethnic background	27 (0.5%)
Asian / Asian British	
Indian	146 (2.9%)
Pakistani	105 (2.1%)
Bangladeshi	50 (1.0%)
Chinese	49 (1.0%)
Any other Asian background	51 (1.0%)
Black / African / Caribbean / Black British	
African	128 (2.5%)
Caribbean	71 (1.4%)
Any other Black / African / Caribbean background	16 (0.3%)
Other ethnic group	
Arab	13 (0.3%)
Any other ethnic group	13 (0.3%)
Marital status:	
Single	1672 (32.7%)
Married or civil partnership	2476 (48.4%)
Cohabiting	555 (10.9%)
Separated	229 (4.5%)
Widowed	182 (3.6%)
Highest level of education:	
No qualifications	330 (6.5%)
GCSEs grades A*−C (or equivalent)	1359 (26.6%)
AS levels (or equivalent)	254 (5.0%)
A levels (or equivalent)	1355 (26.5%)
Certificate of higher education (e.g. BA, BSc, or equivalent)	1256 (24.6%)
Post-graduate qualifications (e.g. MA, MSc, PhD, DPhil)	560 (11.0%)
Total household income:	
Less than £15,000	869 (17.0%)
£15,000−£19,999	633 (12.4%)
£20,000−£29,999	1015 (19.8%)
£30,000−£39,999	791 (15.5%)
£40,000−£49,999	630 (12.3%)
£50,000−£59,999	413 (8.1%)
£60,000−£69,999	243 (4.8%)
£70,000−£99,999	315 (6.2%)
£100,000−£149,999	144 (2.8%)
£150,000 and above	61 (1.2%)
Housing situation:	
Rented from council	957 (18.7%)
Rented from private landlord	1090 (21.3%)
Homeowner	2825 (55.2%)
Other	242 (4.7%)
Region:	
North East	192 (3.8%)
North West	567 (11.1%)
Yorkshire and the Humber	414 (8.1%)
East Midlands	357 (7.0%)
West Midlands	470 (9.2%)
East	405 (7.9%)
London	723 (14.1%)
South East	731 (14.3%)
South West	427 (8.3%)
Wales	257 (5.0%)
Scotland	465 (9.1%)
Northern Island	106 (2.1%)
Pre-coronavirus employment status:	
Unemployed	455 (8.9%)
Employed full-time	2228 (43.6%)
Employed part-time	717 (14.0%)
Self-employed	275 (5.4%)
Retired	986 (19.3%)
Student	155 (3.0%)
Homemaker	298 (5.8%)
Employment change due to coronavirus:	
None	3010 (58.9%)
None, but working from a different location (e.g. work from home)	629 (12.3%)
Working hours have reduced	687 (13.4%)
Working hours have increased	189 (3.7%)
Furlough	227 (4.4%)
Newly unemployed	209 (4.1%)
Newly employed (full-time)	106 (2.1%)
Newly employed (part-time)	57 (1.1%)
Had COVID-19:	
Yes, a positive test	195 (3.8%)
No, a negative test	984 (19.2%)
May have had it but not been tested	600 (11.7%)
Not had it but not been tested	3222 (63.0%)
Other	113 (2.2%)
High risk for severe COVID-19 course	
Low risk	2434 (47.6%)
Moderate risk	1869 (36.5%)
Very high risk	811 (15.9%)

### Frequency of vaccine hesitancy

The mean score on the Oxford COVID-19 vaccine hesitancy scale was 13.6 (SD=7.3). The factor score for the Oxford COVID-19 vaccine hesitancy scale was significantly associated with the factor score for the vaccine hesitancy scale, *r*=0.47, *p* < 0.001. The frequencies of endorsement of each of the Oxford COVID-19 vaccine hesitancy scale items are summarised in Table 2.

**Table 2.** Endorsement of vaccine hesitancy items

Item	Response	%	*n*
1. Would you take a COVID-19 vaccine (approved for use in the UK) if offered?	Definitely	48.6	2485
	Probably	25.2	1291
	I may or I may not	12.7	647
	Probably not	5.7	293
	Definitely not	6.1	314
	Don't know	1.6	84
2. If there is a COVID-19 vaccine available	I will want to get it as soon as possible	32.9	1682
	I will take it when offered	36.6	1873
	I'm not sure what I will do	15.6	796
	I will put off (delay) getting it	6.8	348
	I will refuse to get it	6.4	327
	Don't know	1.7	88
3. I would describe my attitude towards receiving a COVID-19 vaccine as:	Very keen	32.7	1672
	Pretty positive	34.6	1772
	Neutral	14.5	743
	Quite uneasy	10.8	551
	Against it	6.1	311
	Don't know	1.3	65
4. If a COVID-19 vaccine was available at my local pharmacy, I would:	Get it as soon as possible	46.3	2369
	Get it when I have time	23.5	1201
	Delay getting it	8.2	418
	Avoid getting it for as long as possible	6.1	310
	Never get it	8.0	409
	Don't know	8.0	407
5. If my family or friends were thinking of getting a COVID-19 vaccination, I would:	Strongly encourage them	38.6	1972
	Encourage them	28.4	1450
	Not say anything to them about it	18.1	924
	Ask them to delay getting the vaccination	4.7	239
	Suggest that they do not get the vaccination	5.0	254
	Don't know	5.4	275
6. I would describe myself as:	Eager to get a COVID-19 vaccine	32.2	1648
	Willing to get the COVID-19 vaccine	39.2	2006
	Not bothered about getting the COVID-19 vaccine	10.3	529
	Unwilling to get the COVID-19 vaccine	8.8	451
	Anti-vaccination for COVID-19	5.1	260
	Don't know	4.3	220
7. Taking a COVID-19 vaccination is:	Really important	43.2	2210
	Important	31.6	1616
	Neither important nor unimportant	11.8	604
	Unimportant	3.4	175
	Really unimportant	4.5	231
	Don't know	5.4	278

In all, 3892 (76.1%) participants did not endorse any clear vaccine hesitancy response (a response rating of 4 or 5) on any of the seven items, while 1222 (23.9%) endorsed at least one of the items with a hesitant response. Further, 596 (11.7%) participants endorsed four or more of the seven items (i.e. over half) with a clear vaccine hesitancy response (a rating of 4 or 5). This group can be considered strongly vaccine hesitant. 196 (3.8%) participants endorsed all seven items with a clear vaccine hesitancy response, and this can be considered a very extreme group.

4193 (82.0%) participants endorsed at least one of the seven items with a clear positive response (a rating of 1 or 2), while 921 (18.0%) did not rate any of the items with a clear positive response. 3669 (71.7%) participants endorsed four or more of the seven items with a clear positive response (a rating of 1 or 2). This group can be considered clearly positive about a COVID-19 vaccine. 2823 (55.2%) participants endorsed all seven items with a clear positive response.

1842 (36.0%) participants endorsed at least one of the items with the middle response (a rating of 3), while 3272 (64.0%) did not endorse any middle responses. 466 (9.1%) participants endorsed four or more of the seven items with the middle response. This group can be considered as consistently very unsure about a COVID-19 vaccine.

737 (14.4%) participants endorsed at least one of the items with a do not know response, while 4377 (85.6%) did not use this response option. 106 (2.1%) participants endorsed four or more of the seven items with a do not know response. This group can also be considered as consistently very unsure.

There were also individuals who showed two different sets of three consistent responses (but no set of four consistent responses). 34 (0.7%) participants endorsed extreme hesitancy responses (a response rating of 4 or 5) for three of the seven items and three undecided middle options, 47 (0.9%) endorsed three clear positive responses (a rating of 1 or 2) and three undecided middle options, 19 (0.4%) endorsed three undecided middle options and three don't know options, and 11 (0.2%) endorsed three clear positive and three clear negative views.

In summary, 71.7% of the population responded in a consistently positive way towards taking a COVID-19 vaccine, 16.6% were very unsure about taking a COVID-19 vaccine, and 11.7% were strongly hesitant.

### Vaccine hesitancy and socio-demographic factors

The associations of vaccine hesitancy and socio-demographic factors, tested in individual linear regressions, are summarised in Table 3. Vaccine hesitancy is associated with lower age, female gender, lower education, lower income, black and mixed ethnicities, not being single or widowed, not being a homeowner, not being employed full-time, not retired, a change in working, and having a child at school. The *R*^2^ scores indicate that each variable explains only a small percentage of vaccine hesitancy, with age explaining the highest amount (3.8%). When all the socio-demographic variables were entered into a multiple regression (see online supplementary materials), the *R*^2^ was 0.098. COVID-19 vaccine hesitancy was significantly lower in those at very high risk of a severe COVID-19 illness course compared with those at moderate risk or low risk, and those at moderate risk were significantly lower in hesitancy scores than those at low risk of a severe illness course. There was no evidence of interactions between age, gender, and income in explaining vaccine hesitancy (*p* > 0.1). Hesitancy was not associated with political views, but more right-wing political views were associated with coronavirus conspiracy beliefs, *B*=0.093, standard error=0.007, Beta=0.17, *p* < 0.001, *R*^2^=0.03. A hierarchical regression showed that both the linear political item, *B*=−0.115, standard error=0.030, Beta=0.21, *p* < 0.001, and a quadratic term (the political item squared), *B*=0.026, standard error=0.004, Beta=0.40, *p* < 0.001 were significant predictors of general coronavirus conspiracy scores.

**Table 3.** Associations of demographic factors with vaccine hesitancy (individual regressions).

	B	Standard error	*p*-value	Standardised coefficient	*R*^2^
Age	−0.031	0.001	<0.001	−0.20	0.038
Sex	0.224	0.031	<0.001	0.10	0.010
Education	−0.052	0.010	<0.001	−0.07	0.005
Religion	−0.008	0.007	0.290	−0.02	0.000
Politics	0.009	0.012	0.462	0.01	0.000
Income	−0.054	0.007	<0.001	−0.11	0.012
Ethnicity: White					
Asian	0.100	0.056	0.074	0.02	0.012
Black	0.542	0.087	<0.001	0.10	
Mixed	0.316	0.107	0.003	0.04	
Other	0.245	0.235	0.297	0.0216	
Relationship status: Married or civil partnership					
Single	−0.286	0.035	<0.001	−0.13	0.018
Cohabit	0.041	0.057	0.477	0.01	
Separated	−0.106	0.081	0.193	−0.02	
Widowed	−0.377	0.085	<0.001	−0.06	
Region: South East					
London	0.049	0.092	0.591	0.02	0.008
North West	0.088	0.098	0.371	0.02	
West Midlands	0.052	0.099	0.602	0.01	
Scotland	−0.099	0.094	0.291	−0.03	
South West	−0.002	0.096	0.986	−0.00	
Yorkshire and the Humber	0.132	0.103	0.199	0.03	
East	0.221	0.092	0.017	0.07	
East Midlands	0.114	0.097	0.242	0.03	
Wales	−0.120	0.104	0.247	−0.02	
North East	0.101	0.095	0.287	0.03	
Northern Ireland	0.199	0.143	0.165	0.03	
Housing: Rented from council					
Rented from private landlord	0.008	0.053	0.885	0.00	0.022
Homeowner	−0.328	0.044	<0.001	−0.15	
Other	0.001	0.084	0.991	0.00	
Employment status before epidemic: Unemployed					
Full time	−0.162	0.062	0.009	−0.07	0.031
Part-time	−0.137	0.070	0.052	−0.04	
Self-employed	−0.093	0.092	0.314	−0.02	
Retired	−0.581	0.065	<0.001	−0.21	
Student	−0.104	0.105	0.321	−0.07	
Homemaker	0.080	0.090	0.376	0.02	
Employment change due to epidemic: None					
Working from a different location	−0.074	0.046	0.113	−0.02	0.008
Working hours have reduced	0.065	0.044	0.144	0.02	
Working hours have increased	0.206	0.075	0.006	0.04	
Furlough	0.228	0.084	0.007	0.04	
Unemployed	0.363	0.085	<0.001	0.07	
Newly employed (full-time)	0.082	0.116	0.476	0.01	
Newly employed (part-time)	0.104	0.141	0.461	0.01	
Child at school	−0.154	0.034	<0.001	−0.07	0.004
Risk of severe COVID-19 illness course: Low risk					
Moderate risk	−0.250	0.033	<0.001	−0.11	0.025
Very high risk	−0.456	0.044	<0.001	−0.15	

### Vaccine hesitancy and adherence to distancing guidelines

Higher levels of vaccine hesitancy are associated with less following of all guidelines and less likelihood of taking a diagnostic or antibody test (all *p*-values<0.001) (see Table 4).

**Table 4.** Individual correlations of government social distancing guidelines with vaccine hesitancy (all *p*-values <0.001).

Item	Vaccine hesitancy factor score
Overall, how much have you followed guidance from the government about COVID-19?	−0.31
How much will you follow *future* guidance from the government about COVID-19?	−0.37
Only socialise indoors with up to six people if you do not live with them, and maintain social distancing with them. This includes when dining out or going to the pub (except if an exception applies to you e.g. more than six in your household)	−0.18
Only socialise outdoors in a group of up to six people from different households and maintain social distancing with them (except if an exception applies to you e.g. more than six in your household)	−0.19
Limit the number of people you see socially, especially over short periods of time	−0.23
Not hold or attend gatherings where it is difficult to maintain social distancing and avoid social interaction	−0.19
Only stay overnight away from your home in groups of up to six people	−0.14
When asked, provide your contact details to a business so that you can be contacted as needed by the NHS Test and Trace programme	−0.33
Wear a face covering in indoor public settings.	−0.29
Wash your hands with soap and water often, for at least 20 seconds	−0.22
If you do go out, staying 2 m apart from other people at all times	−0.26
Take a COVID-19 diagnostic test (to test if you currently have COVID-19) if offered?	0.52
Take a COVID-19 antibody test (to test if you have previously had COVID-19) if offered	0.50

### Conspiracy belief endorsement

Tables 5 and 6 provide a summary of prevalence rates for misinformation and conspiracy beliefs about the pandemic and vaccinations. It can be seen that there is appreciable endorsement of coronavirus conspiracy beliefs and vaccination conspiracy beliefs.

**Table 5.** Endorsement of coronavirus conspiracy beliefs (OCEANS coronavirus conspiracy scale).

	Do not agree	Agree a little	Agree moderately	Agree a lot	Agree completely	Don't know
1. The virus is a hoax.	3682 (72.0%)	355 (6.9%)	388 (7.6%)	216 (4.2%)	197 (3.9%)	276 (5.4%)
2. The virus is manmade.	1542 (30.2%)	772 (15.1%)	741 (14.5%)	645 (12.6%)	746 (14.6%)	668 (13.1%)
3. The spread of the virus is a deliberate attempt to reduce the size of the global population.	2604 (50.9%)	607 (11.9%)	584 (11.4%)	402 (7.9%)	427 (8.3%)	490 (9.6%)
4. The spread of the virus is a deliberate attempt by governments to gain political control.	2799 (54.7%)	569 (11.1%)	544 (10.6%)	372 (7.3%)	386 (7.5%)	444 (8.7%)
5. The spread of the virus is a deliberate attempt by a group of powerful people to make money.	2794 (54.6%)	536 (10.5%)	512 (10.0%)	372 (7.3%)	415 (8.1%)	485 (9.5%)
6. The spread of the virus is a deliberate attempt by one nation to destabilise another.	2539 (49.6%)	642 (12.6%)	538 (10.5%)	400 (7.8%)	455 (8.9%)	540 (10.6%)
7. The spread of the virus is a deliberate attempt by global companies to take control.	2882 (56.4%)	493 (9.6%)	461 (9.0%)	397 (7.8%)	382 (7.5%)	499 (9.8%)
8. COVID-19 is a bioweapon developed by China to destroy the West.	2321 (45.4%)	701 (13.7%)	538 (10.5%)	395 (7.7%)	439 (8.6%)	720 (14.1%)
9. The virus is a biological weapon manufactured by the United States.	3277 (64.1%)	345 (6.7%)	350 (6.8%)	314 (6.1%)	207 (4.0%)	621 (12.1%)
10. The United Nations (UN) and World Health Organisation (WHO) have manufactured the virus to take global control.	3358 (65.7%)	350 (6.8%)	393 (7.7%)	246 (4.8%)	212 (4.1%)	555 (10.9%)
11. Jews have created the virus to collapse the economy for financial gain.	3730 (72.9%)	214 (4.2%)	308 (6.0%)	242 (4.7%)	165 (3.2%)	455 (8.9%)
12. The elite have created the virus in order to establish a one-world government.	3357 (65.6%)	312 (6.1%)	343 (6.7%)	300 (5.9%)	272 (5.3%)	530 (10.4%)
13. Bill Gates has created the virus in order to reduce the world population.	3572 (69.8%)	246 (4.8%)	308 (6.0%)	268 (5.2%)	239 (4.7%)	481 (9.4%)
14. Big Pharma created COVID-19 to profit from the vaccines.	3333 (65.2%)	342 (6.7%)	346 (6.8%)	288 (5.6%)	256 (5.0%)	549 (10.7%)
15. COVID-19 is being used by the government to implement a police state.	2992 (58.5%)	611 (11.9%)	473 (9.2%)	307 (6.0%)	300 (5.9%)	431 (8.4%)
16. COVID-19 is caused by 5 G and is a form of radiation poisoning transmitted through radio waves.	3698 (72.3%)	236 (4.6%)	358 (7.0%)	270 (5.3%)	164 (3.2%)	388 (7.6%)
17. The virus is a smokescreen for a global conspiracy that swapped the real world with a simulation.	3546 (69.3%)	322 (6.3%)	358 (7.0%)	261 (5.1%)	205 (4.0%)	422 (8.3%)
18. COVID-19 was created to force everyone to get vaccinated.	3393 (66.3%)	373 (7.3%)	333 (6.5%)	306 (6.0%)	291 (5.7%)	418 (8.2%)
19. The vaccine will be used to carry out mass sterilisation.	3470 (67.9%)	275 (5.4%)	323 (6.3%)	296 (5.8%)	284 (5.6%)	466 (9.1%)
20. The World Health Organisation (WHO) already has a vaccine and are withholding it.	3278 (64.1%)	372 (7.3%)	338 (6.6%)	299 (5.8%)	242 (4.7%)	585 (11.4%)
21. Antibody testing is a plot to harvest our DNA.	3355 (65.6%)	310 (6.1%)	356 (7.0%)	301 (5.9%)	282 (5.5%)	510 (10.0%)

**Table 6.** Endorsement of general vaccination conspiracy beliefs (vaccine conspiracy beliefs scale).

	Strongly disagree	Disagree	Somewhat disagree	Neutral	Somewhat agree	Agree	Strongly agree
Vaccine safety data are often fabricated (made up).	1106 (21.6%)	1055 (20.6%)	652 (12.7%)	1407 (27.5%)	475 (9.3%)	222 (4.3%)	197 (3.9%)
Immunising children is harmful and this fact is covered up.	1644 (32.1%)	1135 (22.2%)	655 (12.8%)	970 (19.0%)	354 (6.9%)	201 (3.9%)	155 (3.0%)
Pharmaceutical companies cover up the dangers of vaccines.	1016 (19.9%)	1050 (20.5%)	692 (13.5%)	1158 (22.6%)	689 (13.5%)	265 (5.2%)	244 (4.8%)
People are deceived about the effectiveness of vaccines.	1086 (21.2%)	1073 (21.0%)	644 (12.6%)	1186 (23.2%)	623 (12.2%)	291 (5.7%)	211 (4.1%)
Vaccine effectiveness data are often fabricated (made up).	1221 (23.9%)	1055 (20.6%)	632 (12.4%)	1194 (23.3%)	554 (10.8%)	236 (4.6%)	222 (4.3%)
People are deceived about vaccine safety.	1155 (22.6%)	1056 (20.6%)	598 (11.7%)	1150 (22.5%)	615 (12.0%)	310 (6.1%)	230 (4.5%)
The government is trying to cover up the link between vaccines and autism.	1526 (29.8%)	906 (17.7%)	537 (10.5%)	1176 (23.0%)	475 (9.3%)	254 (5.0%)	240 (4.7%)

### Vaccine hesitancy explanatory factors

The simple associations of the factor scores for vaccine hesitancy and the explanatory factors are shown in Table 6. Every factor score was significantly associated with vaccine hesitancy (p < 0.001) (Table 7).

**Table 7.** Individual correlations of explanatory factor scores with vaccine hesitancy (all *p*-values < 0.001).

Explanatory factor	Vaccine hesitancy
Importance of a COVID-19 vaccine	0.94
Beliefs that may get COVID-19 and the vaccine will work	0.85
Speed of vaccine development	0.85
Vaccine side effects	0.93
General knowledge about vaccines	−0.49
Knowledge about childhood vaccines	−0.44
Interpersonal disrespect by doctors	0.37
Respect from doctors	0.50
Negative views of vaccine developers	0.54
Positive attitude to doctors	−0.45
Negative attitude to doctors	0.32
Positive attitude to medicine	−0.47
Negative attitude to medicine	0.31
Social ladder relative to other people in the UK	0.15
Social ladder relative to other people in their community	0.13
Negative beliefs about the self	0.09
Positive beliefs about the self	−0.08
Positive GP experiences	0.33
Positive NHS experiences	0.45
Negative NHS experiences	0.28
Coronavirus conspiracy beliefs (general)	0.38
Vaccine conspiracy beliefs	0.48
Others disrespectful	−0.12
Others react negatively	−0.11
Anger	0.12
Need for chaos	0.20
Libertarian views	0.11
Populist views	0.16
Religious influence on health behaviour	0.06
Illness as punishment for sin	0.11

### Model 1: Beliefs about a COVID-19 vaccine

**Fig. 1.**
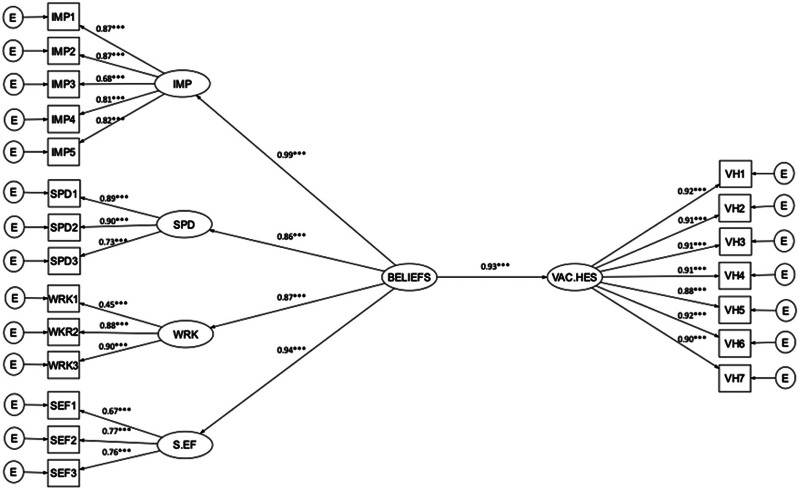
Structural equation model 1: Beliefs about a COVID-19 vaccine and vaccine hesitancy. *Note*: *p < 0.01, **p < 0.01, ***p < 0.001. IMP = collective importance; SPD = speed of development; WRK = vaccine will be effective; S.EF = side effects; VAC.HES = vaccine hesitancy.

There were high correlations between the four factors derived for beliefs about a COVID-19 vaccine (assessed by the Oxford COVID-19 vaccine confidence and complacency scale) (see online supplementary materials, Table S1). Therefore, the four factors were merged into a higher-order construct labelled as ‘Beliefs about a COVID-19 vaccine’. The CFA model with a higher-order construct suggested a very good model fit (FIML, *χ*2(73, *N*= 5081)=1094.88, *p* < 0.001, RMSEA=0.05; SRMR=0.03; CFI=0.97; TLI=0.96). The strongest individual factor contributing to the higher-order factor was beliefs about the collective importance of a COVID-19 vaccine. The data were subsequently fitted to the hypothesised structural model and the fit indexes indicated an excellent model fit (FIML, *χ*2(184, *N*=5109)=2220.53, *p* < 0.001, RMSEA=0.05; SRMR=0.03; CFI=0.97; TLI=0.97). Beliefs about a COVID-19 vaccine are a major predictor of vaccine hesitancy (unstandardised *b*=1.63, standard error=0.05, *p* < 0.001), accounting for 86% of the variance in vaccine hesitancy. The SEM model diagram with factor loadings and the standardised regression weights is shown in Figure 1 (also see online supplementary materials, Table S2).

### Model 2: Mistrust and vaccine hesitancy

The initial CFA model indicated a very good model fit (FIML, *χ*2(5307, *N*=5,114)=20532.83, *p* < 0.001, RMSEA=0.02; SRMR=0.05; CFI=0.95; TLI=0.95). However, inspection of the factor correlations in the CFA model revealed several factors correlating substantially amongst the latent constructs (see online supplementary materials, Table S3). These factors were merged into two distinct higher-order factors. The higher-order factors were termed as ‘Mistrust’ and ‘Positive Healthcare’, respectively. The resulting model with two higher-order factors indicated adequate fit (FIML, *χ*2(5481, *N*=5114)=27070.50, *p* < 0.001, RMSEA=0.03; SRMR=0.07; CFI=0.93; TLI=0.93). Details of this SEM model can be found in online supplementary materials, Table S4 and Fig. S1.

The backward elimination procedure reduced the number of predictors from 14 to 10. Direct effects of four factors (religious influence on health behaviour, others reacting negatively, libertarian beliefs, and populist beliefs) were fixed to zero in the structural model. The SEM model after this backward elimination procedure indicated reasonable fit (FIML, *χ*2(5697, *N*=5114)=28926.01, *p* < 0.001, RMSEA=0.03; SRMR=0.07; CFI=0.93; TLI=0.93). Since the reduced SEM model is considered nested from the baseline model where the relations between the constructs were freely estimated, an analysis of variance (ANOVA) was conducted to test for the best-fitting model from all the models, which indicated that the final reduced model (SEM model 5) was preferable.

**Fig. 2.**
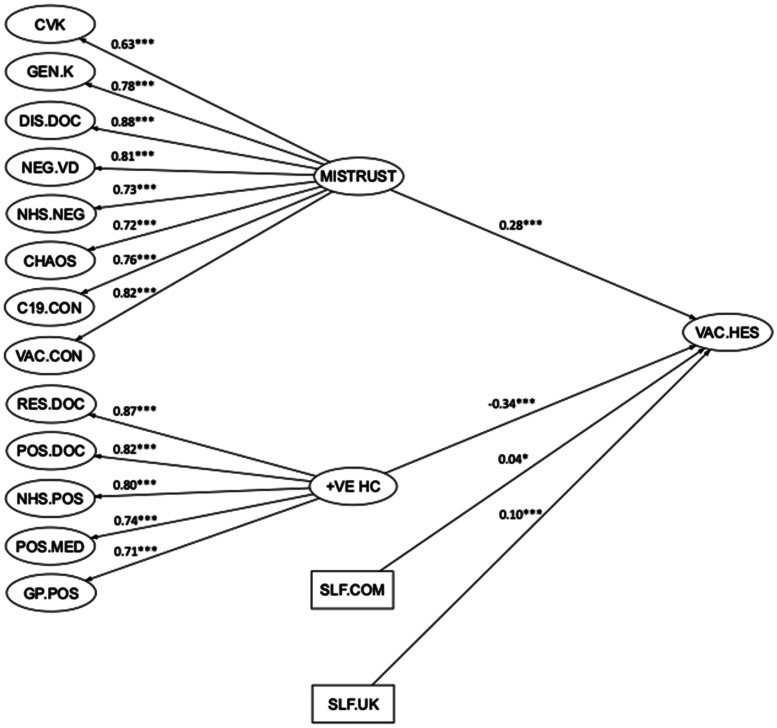
Structural equation model 2 (final): Mistrust. *Note:* *p < 0.01, **p < 0.01, ***p < 0.001. CVK = Knowledge about childhood vaccinations; GEN.K = general knowledge about vaccines; DIS.DOC = interpersonal disrespect from doctors; NEG.VD = negative views of vaccine developers; NHS.NEG= negative experiences of NHS care; CHAOS = need for chaos; C19.CON = coronavirus general conspiracy beliefs; VAC.CON = vaccination conspiracy beliefs; RES.DOC = respect from doctors; POS.DOC = positive attitudes to doctors; NHS.POS = positive NHS experiences; POS.MED = positive attitudes to medication; GP.POS = positive GP experiences; MISTRUST = higher order excessive mistrust factor; +VE HC = higher-order positive healthcare experiences factor; SLF.COM = subjective sense of social status in community; SLF.UK = subjective sense of social status in UK; VAC.HES = vaccine hesitancy.

Further review of the beta estimates suggested that several predictors may be acting as suppressors (see online supplementary materials). Thus, the direct effects of five predictors (negative self, anger, illness as punishment, others disrespectful, positive self) were fixed to zero in the structural model. The variance inflation factor and tolerance levels of all remaining variables were less than 5 and tolerance values greater than 0.1, indicating an absence of multicollinearity (see online supplementary materials, Table S7). The final SEM model indicated reasonable fit to the data (FIML, *χ*2(5702, *N*=5114)=29308.76, *p* < 0.001, RMSEA=0.03; SRMR=0.070; CFI=0.93; TLI=0.92) and the variance explained by the structural model was 32%. Online supplementary materials, Table S8 summarises the parameter estimates of the structural model. The reduced SEM model (i.e. SEM model 5) is shown in Figure 2. The higher-order factors had the strongest associations with vaccine hesitancy. The higher-order factor of positive experiences of healthcare was associated with lower levels of COVID-19 vaccine hesitancy; the higher-order factor of mistrust was associated with higher levels of COVID-19 hesitancy. A lower subjective sense of self in relation to others (the UK or community) was also associated with greater vaccine hesitancy.

**Fig. 3.**
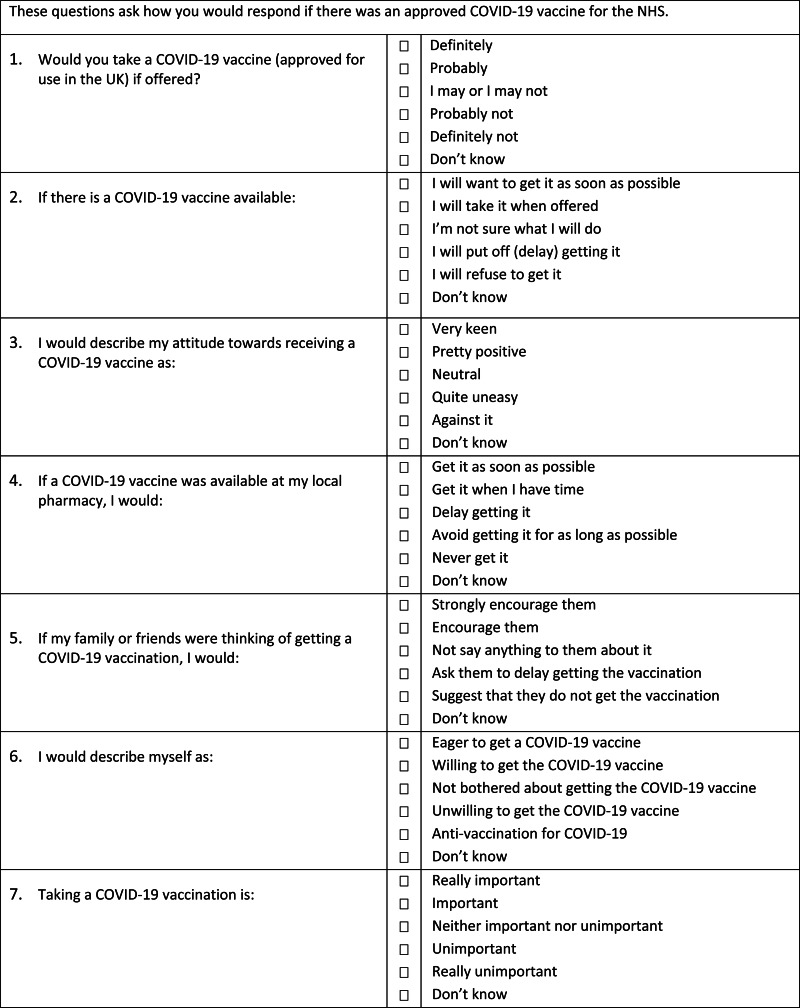
Oxford Covid-19 vaccine hesitancy scale.

## Discussion

The substantial majority view in the UK public that vaccines are safe, effective, and important remains intact, but appears vulnerable given the presence of significant proportions of the population either very doubtful or rejecting of a COVID-19 vaccine. The danger is of a tipping point being approached, whereby mistrust of vaccines becomes mainstream, with negative consequences for individuals, healthcare services, and potentially herd immunity. With the caution that this was an online non-probability survey, the majority of the population – around 72% − are likely to accept reasonably readily a COVID-19 vaccine. It is possible that this majority will remain robust in the months ahead, but this is likely to be tested. The remainder of the population, a sizeable minority, are clearly very ambivalent (17%) about a COVID-19 vaccine or are unlikely to take it (12%). Around one in 20 people would even describe themselves as explicitly anti-vaccination for COVID-19. When the time comes, the behaviour of those who are ambivalent about a vaccination will be a defining factor in the success of implementation of an approved vaccine. Socio-demographics do not explain vaccine hesitancy to any helpful degree. Doubt is spread relatively evenly in the population − it is not circumscribed to specific groups − which may be another indicator of the issue being at a tipping point. The understanding of the causes of vaccine hesitancy has never been more important.

There are a set of beliefs, tightly bound to willingness to take a COVID-19 vaccine, that are plausible drivers of vaccine up-take. Fused with acceptance of a vaccine are beliefs about the collective importance: that a vaccine will save lives, help the community, and that it will be dangerous if many people do not get vaccinated. This chimes with evidence that emphasising collective − rather than personal – responsibility may lead to greater change in individuals’ behaviour (e.g. Obradovich & Guenther, 2016). Pro-social behaviour may be especially self-rewarding and therefore affect behaviour (e.g. Aknin et al., 2012). Betsch and colleagues (2015) in an online experiment with fictitious disease scenarios found that explaining the social benefits of herd immunity can increase intent to be vaccinated. In the OCEAN survey, beliefs about whether a vaccine will provide freedom or restriction were part of the collective importance factor too. There were three other key types of beliefs about a COVID-19 vaccine: if a respondent thought it likely that they would be infected and the vaccine would work; whether speed of development of the vaccine would affect safety and efficacy; and the degree to which receiving the vaccine may be physically unpleasant and that the recipient would feel experimented on. All these findings are highly consistent with the framing in the vaccine hesitancy literature of the importance of complacency and confidence in vaccine decision-making.

We also set out to determine the more distal individual factors that may affect vaccine up-take. The hypothesis was that mistrust may skew vaccine decision making. The modelling indicated a key higher-order factor of excessive mistrust that was associated with vaccine hesitancy. This factor included coronavirus conspiracy beliefs, which were at a troubling prevalence comparable to our first OCEAN survey in May (Freeman et al., 2020). We also note that a significant minority hold general vaccine conspiracy beliefs, while an even higher proportion is neutral as to whether vaccine conspiracy beliefs are true or false. These fringe beliefs do appear to have become mainstream. Negative perceptions of doctors (‘They do not really care about me’), vaccine developers (‘They just want to make money’), and healthcare experiences (‘The NHS treats my community badly’) feed into the distrust. There also appears to be a contribution of anger: a need for chaos (‘I think society should be burned to the ground’), anger directed at societal structures, contributed to the higher-order factor. Individuals who were vaccine hesitant also viewed themselves as lower on the social ladder, indicating a degree of perception of vulnerability with regards to other people. Against this mistrust and vulnerability, perceived positive healthcare experiences were connected to lower vaccine hesitancy. Positive GP experiences (‘My GP is polite and considerate’), views of doctors (‘They have my best interests at heart’), medicine (‘The improved health of the nation is due to effective medicine’), and NHS experiences (‘Staff have gone out of their way to help’) were connected with greater enthusiasm about a COVID-19 vaccine. In sum, factors that erode trust in healthcare and society are likely to be pernicious for vaccine up-take but positive experiences of institutions, those that rebuild trust, are likely to maintain the consensus of the importance of being vaccinated.

The overall immediate implications of our explanatory work are the potential importance of emphasising in public health messaging the prosocial benefits of a COVID-19 vaccine, as well as the necessity to be transparent about safety and efficacy. Public health communication may need to be carefully attuned to the different kinds of collective identities and benefits in order to resonate with pro-social motives. In this way, such messaging may not only help consolidate the majority willing to be vaccinated but perhaps increase the willingness of those who are hesitant. Careful testing and refining of messaging across the spectrum of hesitancy will be needed. The survey findings also indicate that materials may benefit from highlighting the many positive contributions that NHS staff make. There is an urgent need to counter misinformation, ideally by ‘prebunking’ or inoculation (Wong, 2016; van der Linden et al., 2017), and provide strong presentation of accurate information. The findings also reiterate the longer-term work needed to rebuild trust in experts and institutions. However there are limitations to the survey. Foremost, we used a non-probability online quota sampling method, which will have introduced bias to who was approached to take part. We do know that, taken as a whole, the respondents in this survey were broadly representative of the adult general population on a number of basic demographic features (although, for example, levels of higher education were slightly high) but not that individual respondents were representative of the general population. This means that prevalence estimates especially must be treated with caution, as well as the identification of demographic predictors. It is plausible that the survey method will have introduced less bias into the explanatory parts of the study (Pasek, 2015). A further caution concerns our primary measure: we do not know the extent to which expressed intent to take a vaccine is associated with actual behaviour. The key limitation for the conclusions concerning the explanatory variables is that the survey is cross-sectional. We do not know whether the beliefs, attitudes, and perceived experiences actually cause willingness to take a COVID-19 vaccine. Our plan is to use detailed qualitative interviewing, guided by the results of the survey, to deepen our understanding of vaccine hesitancy, and then conduct experimental tests to assess change and causation.

## Supplementary material

For supplementary material accompanying this paper visit https://doi.org/10.1017/S0033291720005188. click here to view supplementary material
