# Supplementary material for: COVID-19 vaccine hesitancy in the UK: the Oxford coronavirus explanations, attitudes, and narratives survey (Oceans) II
Source: Psychol Med. 2020 Dec 11:1–15. doi: 10.1017/S0033291720005188 (PMC7804077; doi:10.1017/S0033291720005188)
Supplement: Supplementary file 1 [file S0033291720005188sup.zip › S0033291720005188sup001.docx]

**Items in each factor**

Oxford COVID-19 Vaccine Confidence & Complacency Scale.

Collective importance of a COVID-19 vaccine (items B20, B21, B24, B28, B32); beliefs that the respondent may get COVID-19 and the vaccine will work (items B16, B17, B18); speed of vaccine development (items B22, B23, B33); and side effects (items B26, B27, B34).

Vaccine Hesitancy Scale (VHS) (Shapiro et al, 2018).

Items C1, C2, C3, C4, C6, C7, C8.

Vaccination Knowledge Scale (Zingg & Siergrist, 2012).

General knowledge about vaccines (items D1.1, D1.2, D1.3, D1.8) and knowledge about childhood vaccines (items D1.4, D1.5, D1.7, D1.9).

Oxford Trust in Doctors and Developers Questionnaire).

Interpersonal disrespect by doctors (items G1.2, G1.5, G1.7, G1.8, G1.9, G1.11); respect from doctors (items G1.1, G1.3, G1.6) and negative views of vaccine developers (items, G1.13, G1.14, G1.15, G1.16).

Attitudes to Doctors and Medicine Questionnaire (Marteau, 1990).

Positive attitude to doctors (items H1.2, H1.3, H1.4); negative attitude to doctors (items H1.6, H1.7, H1.8, H1.10); positive attitude to medicine (items H1.11, H1.13, H1.14); and negative attitude to medicine (items H1.15, H1.17, H1.18, H1.19).

Brief Core Schema Scales – Self scales (BCSS) (Fowler et al, 2006).

Negative self factor (items J1.2, J1.3, J1.4, J1.5, J1.6); positive self factor (items J1.7, J1.9, J1.10, J1.12).

General Practice Assessment Questionnaire-R2 (GPAQ-R2) (Rowland et al, 2013).

Items K1.2, K1.4, K1.6, K1.7, K1.8.

NHS experience questionnaire.

Positive NHS experiences (items K3.1, K3.3, K3x2.1); negative NHS experiences (items K3.2, K3.4, K3.5, K3x2.3).

OCEANS Coronavirus Conspiracy Scale (Freeman et al, 2020).

General coronavirus conspiracy beliefs: L1.1, L1.3, L1.4, L1.5, L1.6, L1.7.

Vaccine Conspiracy Beliefs Scale (Shapiro et al, 2016).

All seven items.

Everyday discrimination scale (Williams et al, 1997).

Others disrespectful (items O1.2, O1.3, O1.4, O1x2.3); others react negatively (items O1x2.1, O1x2.2, O1x2.5).

Dimensions of Anger Reactions-5 (DAR-5) (Forbes et al, 2014).

All five items.

Need for chaos (Petersen et al, 2020).

Items Q1.1, Q1.3, Q1.5, Q1.9, Q1.11.

Lifestyle and Economic/Government Liberty (Iyer et al, 2012).

Items R1.2, R1.5, R1.6, R1.7.

Populist attitudes (Akkerman et al, 2014).

Items R1.8, R1.9, R1.10, R1.11.

Perceived religious influence on health behaviour and illness as punishment by God for sin (Holt et al., 2009).

Religious influence on health behaviour factor (items S1.1, S1.2, S1.3, S1.5, S1.6, S1.7); Illness as punishment for sin factor (items S1.8, S1.9, S1.10, S1.11, S1.12, S1.13, S1.14, S1.15).

# Oxford Coronavirus Explanations, Attitudes, and Narratives Survey II (OCEANS II)

# A. Demographics

1. **What is your current age?**
   - Please specify ________ (Terminate <18 years old)
2. **Please indicate your gender.**

| Male 🞏 | Female 🞏 | Non-binary 🞏 | Prefer not to say 🞏 |
| --- | --- | --- | --- |
|  |  |  |  |

2.a **In which region do you live?**

🞏 North East

🞏 North West

🞏 Yorkshire and the Humber

🞏 East Midlands

🞏 West Midlands

🞏 East

🞏 London

🞏 South East

🞏 South West

🞏 Wales

🞏 Scotland

🞏 Northern Ireland

1. **What is the highest level of education you obtained?**

🞏 No qualifications

🞏 GCSEs grades A*-C (or equivalent)

🞏 AS Levels (or equivalent)

🞏 A Levels (or equivalent)

🞏 Certificate of higher education (e.g. BA, BSc, or equivalent)

🞏 Post graduate qualifications (e.g. MA, MSc, PhD, DPhil)

4.       **Religious beliefs**

|  | Not at all  1 | 2 | 3 | 4 | 5 | 6 | Extremely important  7 |
| --- | --- | --- | --- | --- | --- | --- | --- |
| How important is religion in your life? |  |  |  |  |  |  |  |

5.       **Political beliefs**

In politics, many people use the terms “left” and “right”. When it comes to politics, where do you place yourself on this scale?

|  | Strongly left wing  1 | 2 | 3 | In the centre  4 | 5 | 6 | Strongly right wing  7 |
| --- | --- | --- | --- | --- | --- | --- | --- |
| Please indicate your political beliefs |  |  |  |  |  |  |  |

6.       **What is your total annual household income?**

| Less than £15,000 |  |  | £50,000-£59,999 |  |
| --- | --- | --- | --- | --- |
|  |  |  |  |  |
| £15,000-£19,999 |  |  | £60,000-£69,999 |  |
|  |  |  |  |  |
| £20,000-£29,999 |  |  | £70,000-£99,999 |  |
|  |  |  |  |  |
| £30,000-£39,999 |  |  | £100,000-£149,999 |  |
|  |  |  |  |  |
| £40,000-£49,999 |  |  | £150,000 and above |  |

7. **Please tick the box that best describes your current marital status.**

| Single |  |  | Married/civil partnership |  |
| --- | --- | --- | --- | --- |
|  |  |  |  |  |
| Cohabiting |  |  | Separated |  |
|  |  |  |  |  |
| Widowed |  |  |  |  |

8. **Please tick the box that best describes your ethnicity.**

| _1 White |  |  | _2 Black / African / Caribbean / Black British |  |
| --- | --- | --- | --- | --- |
| 🞏 English / Welsh / Scottish / Northern Irish / British  🞏 Irish  🞏 Gypsy or Irish Traveler  🞏 Any other White background |  |  | 🞏 African  🞏 Caribbean  🞏 Any other Black / African / Caribbean background |  |
| _3 Mixed / Multiple ethnic groups |  |  | _5 Other ethnic group |  |
| 🞏 White and Black Caribbean  🞏 White and Black African  🞏 White and Asian  🞏 Any other Mixed / Multiple ethnic background |  |  | 🞏 Arab  🞏 Other: please specify _________________ |  |
| _4 Asian / Asian British |  |  |  |  |

🞏 Indian

🞏 Pakistani

🞏 Bangladeshi

🞏 Chinese

🞏 Any other Asian background

| Prefer not to say |  |  |  |  |
| --- | --- | --- | --- | --- |

9. **Please tick the box that best describes your current housing situation.**

🞏 Rented from council

🞏 Rented from private landlord

🞏 Homeowner

🞏 Other

10. **Do you have a child at school? Yes / No**

11.**What was your employment/work status before COVID-19?**

🞏 Unemployed

🞏 Employed full-time

🞏 Employed part-time

🞏 Self-employed

🞏 Retired

🞏 Student

🞏 Homemaker

12. **Has your employment changed due to COVID-19?**

🞏 No

🞏 No, but I am working from a different location (e.g. work from home)

🞏 Yes, my working hours have reduced

🞏 Yes, my working hours have increased

🞏 Yes, I am now on furlough

🞏 Yes, I am now unemployed

🞏 Yes, I am now newly employed (full-time)

🞏 Yes, I am now newly employed (part-time)

# B. Views about a COVID-19 vaccine

| These questions are asking about how you would respond if there was an approved COVID-19 vaccine for the NHS. | | |
| --- | --- | --- |
| B1. Would you take a COVID-19 vaccine (approved for use in the UK) if offered? |  | Definitely  Probably  I may or I may not  Probably not  Definitely not  Don’t know |
| B2. If there is a COVID-19 vaccine available: |  | I will want to get it as soon as possible  I will take it when offered  I’m not sure what I will do  I will put off (delay) getting it  I will refuse to get it  Don’t know |
| B3. I would describe my attitude towards receiving a COVID-19 vaccine as: |  | Very keen  Pretty positive  Neutral  Quite uneasy  Against it  Don’t know |
| B4. If my GP surgery texted me to come and receive a COVID-19 vaccine, I would: |  | Make an appointment straight away  Make an appointment  Wait until a reminder  Wait until many reminders  Never make an appointment  Don’t know  I don’t use a mobile phone |
| B5. If a COVID-19 vaccine was available at my local pharmacy, I would: |  | Get it as soon as possible  Get it when I have time  Delay getting it  Avoid getting it for as long as possible  Never get it  Don’t know |
| B6. If my family or friends were thinking of getting a COVID-19 vaccination, I would: |  | Strongly encourage them  Encourage them  Not say anything to them about it  Ask them to delay getting the vaccination  Suggest that they do not get the vaccination  Don’t know |
| B7. If I had (or do have) a child and the school asked for permission to vaccinate my child for COVID-19, I would: |  | Definitely give permission  Probably give permission Possibly give permission  Probably not give permission  Definitely not give permission  Don’t know |
| B8. If we were all required by the government to take a COVID-19 vaccine, I would: |  | Definitely take it  Accept it  Be hesitant and delay it  Refuse  Strongly refuse  Don’t know |
| B9. If my family or friends suggested I get a COVID-19 vaccine I would: |  | Definitely get it  Probably get it  Possibly get it  Ignore them  Tell them they’re wrong  Don’t know |
| B10. If the vaccine was available at my GP surgery I would: |  | Get it as soon as possible  Get it when I have time  Delay getting it  Avoid getting it for as long as possible  Never get it  Don’t know |
| B11. I would describe myself as: |  | Eager to get a COVID-19 vaccine  Willing to get the COVID-19 vaccine  Not bothered about getting the COVID-19 vaccine  Unwilling to get the COVID-19 vaccine  Anti-vaccination for COVID-19  Don’t know |
| B12. Taking a COVID-19 vaccination is: |  | Really important  Important  Neither important nor unimportant  Unimportant  Really unimportant  Don’t know |
| B13. With regards to a COVID-19 vaccine, if I had (or do have) a child: |  | I would definitely get them vaccinated  I would probably get them vaccinated  I may or may not get them vaccinated  I would not get them vaccinated  I would make sure that they were not vaccinated  Don’t know |
| B14. If it was recommended to get a repeat vaccination for COVID-19 every year, I would: |  | Get it every year without fail  Most likely get it every year  Maybe get it every year  Be unlikely to get it every year  Definitely not get it every year  Don’t know |
| B15. I would get a COVID-19 vaccine to protect my friends and family: |  | Definitely  Probably  I may or I may not  Probably not  Definitely not  Don’t know |
| B16. Do you think you will be infected with COVID-19 over the next 12 months? |  | Definitely  Probably  Possibly  Probably not  Definitely not  Don’t know |
| B17. The COVID-19 vaccine is likely to: |  | Work for almost everyone  Work for most people  I am unsure how many people it will work for  Not work for most people  Not work for anyone  Don’t know |
| B18. The COVID-19 vaccine is likely to: |  | Definitely work for me  Probably work for me  May or may not work for me  Probably not work for me  Definitely not work for me  Don’t know |
| B19. If a COVID-19 vaccine becomes available: |  | It will be important for me to get it  It would be helpful if I got it  It may not matter whether I get it or not  I doubt that I need it  I will not need it  Don’t know |
| B20. If I get the COVID-19 vaccine it will be: |  | Really helpful for the community around me  Helpful for the community around me  Neither helpful nor unhelpful for the community around me  Unhelpful for the community around me  Really unhelpful for the community around me Don’t know |
| B21. If individuals like me get the COVID-19 vaccine it will: |  | Save a large number of lives  Save some lives  Have no impact  Lead to more deaths  Lead to a large number of deaths  Don’t know |
| B22. The speed of developing and testing the vaccine means it will be: |  | Really good  Good  Will not affect how good or bad it is  Bad  Really bad  Don’t know |
| B23. The speed of developing and testing the vaccine means it will be: |  | Really safe  Safe  It will not affect how safe it is  Unsafe  Really unsafe  Don’t know |
| B24. If many people do not get the vaccine this: |  | Will be dangerous  May be dangerous  Will have no consequences at all  May be good  Will be good  Don’t know |
| B25. Having the COVID-19 vaccine will be: |  | Very safe  Safe  It is not clear whether it will be safe or unsafe  Unsafe  Very unsafe  Don’t know |
| B26. I expect that receiving the vaccine will be: |  | Hardly noticeable  A little unpleasant  Moderately unpleasant  Painful  Extremely painful  Don’t know |
| B27. The side effects for people of getting the COVID-19 vaccine will be: |  | None  Mild  Moderate  Significant  Life-threatening  Don’t know |
| B28. The COVID-19 vaccine will: |  | Greatly strengthen my immune system  Strengthen my immune system  It will neither strengthen nor weaken my immune system  Weaken my immune system  Greatly weaken my immune system  Don’t know |
| B29. If the vaccine offered is made in a foreign country, rather than in the UK, it will be: |  | Just as good as if made in the UK  A little worse than if made in the UK  Worse than if made in the UK  Potentially harmful  Harmful  Don’t know |
| B30. If I got COVID-19: |  | I’d probably end up in hospital  I would get quite ill  It would be like getting a mild flu  It would hardly be noticeable  I’d be absolutely fine  Don’t know |
| B31. By taking a COVID-19 vaccine, I am: |  | Taking control of the situation  Gaining some control over the situation  Unsure whether I gain or lose control of the situation  Giving up some control of the situation  Giving up all control over the situation  Don’t know |
| B32. Taking the COVID-19 vaccine: |  | Will give me complete freedom to get on with life just as before  Will give me greater freedom  Will have no effect on my freedom  Will restrict my freedom  Will completely restrict my freedom to get on with life  Don’t know |
| B33. Getting the vaccine is a sign of: |  | Great personal strength  Personal strength  Not a sign of personal strength or weakness  Personal weakness  Great personal weakness  Don’t know |
| B34. Taking a new COVID-19 vaccine will make me feel like a guinea pig: |  | Do not agree  Agree a little  Agree moderately  Agree a lot  Completely agree  Don’t know |
| B35. I think that in my community: |  | Everyone will get a COVID-19 vaccine  Most will get a COVID-19 vaccine  About half will get a COVID-19 vaccine  Very few will get a COVID-19 vaccine  No one will get a COVID-19 vaccine  Don’t know |
| B36. Getting a COVID-19 vaccine will make me feel anxious: |  | Not at all  A little  Moderately  Very  Extremely  Don’t know |
| B37. Getting a COVID-19 vaccine will make me feel excited: |  | Not at all  A little  Moderately  Very  Extremely  Don’t know |
| B38. Getting a COVID-19 vaccine will make me feel relieved: |  | Not at all  A little  Moderately  Very  Extremely  Don’t know |
| B39. Getting a COVID-19 vaccine will make me feel angry: |  | Not at all  A little  Moderately  Very  Extremely  Don’t know |
| B40. Getting a COVID-19 vaccine will make me feel disgusted: |  | Not at all  A little  Moderately  Very  Extremely  Don’t know |
| B41. Getting a COVID-19 vaccine will make me feel happy: |  | Not at all  A little  Moderately  Very  Extremely  Don’t know |
| B42. Getting a COVID-19 vaccine will make me feel humiliated: |  | Not at all  A little  Moderately  Very  Extremely  Don’t know |

# C. Views on childhood vaccines (Vaccine Hesitancy Scale)

These questions are asking about general vaccinations in childhood. This is not specifically about COVID-19. For each statement, please indicate how much you disagree or agree:

|  | Strongly disagree | Disagree | Neither agree nor disagree | Agree | Strongly agree |
| --- | --- | --- | --- | --- | --- |
| C1_1 Childhood vaccines are important for children’s health |  |  |  |  |  |
| C1_2 Childhood vaccines are effective |  |  |  |  |  |
| C1_3 Having children vaccinated is important for the health of others in my community |  |  |  |  |  |
| C1_4 All childhood vaccines offered by the government program in my community are beneficial |  |  |  |  |  |
| C1_5 New vaccines carry more risks than older vaccines |  |  |  |  |  |
| C1_6 The information I receive about vaccines from the vaccine program is reliable and trustworthy |  |  |  |  |  |
| C1_7 Getting vaccines is a good way to protect children from disease |  |  |  |  |  |
| C1_8 Generally people should do what doctors or health care providers recommend about vaccines for children |  |  |  |  |  |
| C1_9 I am concerned about serious adverse effects of vaccines |  |  |  |  |  |

# D. True or false questions about vaccines (Vaccination Knowledge Scale)

This is about vaccines in general. It is not specifically about the COVID-19 vaccine. For each statement, please indicate if you think they are correct, incorrect or you don’t know.

|  | Correct | Incorrect | Do not know |
| --- | --- | --- | --- |
| D1_1. Vaccines are superfluous (unnecessary), as diseases can be treated (e.g. with antibiotics). |  |  |  |
| D1_2. Without broadly applied vaccine programmes, smallpox would still exist. |  |  |  |
| D1_3. The efficacy of vaccines has been proven. |  |  |  |
| D1_4. Children would be more resistant if they were not always vaccinated against all diseases. |  |  |  |
| D1_5. Diseases like autism, multiple sclerosis, and diabetes might be triggered through vaccination. |  |  |  |
| D1_6. The immune system of children is not overloaded through many vaccinations. |  |  |  |
| D1_7. Many vaccinations are administered too early, so that the body’s own immune system has no possibility to develop. |  |  |  |
| D1_8. The doses of the vaccines are not dangerous for humans. |  |  |  |
| D1_9. Vaccinations increase the occurrence of allergies. |  |  |  |

# E. Experience of COVID-19

| E1. Have you had COVID-19? |  | Yes, I had a positive test  No, I had a negative test  I think I may have had it but not been tested  I do not think I have had it but not been tested  Other |
| --- | --- | --- |
| E2. Are you considered to be at greater risk for a severe course of COVID-19 (e.g. over 70, underlying health condition, pregnant)? |  | Low risk  Moderate risk  Very high risk |

# F: Questions about the COVID-19 guidelines

Please indicate how much you manage to follow the social distancing guidelines.

|  | Not at all | Occasionally | Some of the time | Most of the time | All of the time |
| --- | --- | --- | --- | --- | --- |
| F3_1 Only socialise indoors with up to six people if you do not live with them, and maintain social distancing with them. This includes when dining out or going to the pub (except if an exception applies to you e.g. more than six in your household) |  |  |  |  |  |
| F3_2 Only socialise outdoors in a group of up to six people from different households and maintain social distancing with them (except if an exception applies to you e.g. more than six in your household) |  |  |  |  |  |
| F3_3 Limit the number of people you see socially, especially over short periods of time |  |  |  |  |  |
| F3_4 Not hold or attend gatherings where it is difficult to maintain social distancing and avoid social interaction |  |  |  |  |  |
| F3_5 Only stay overnight away from your home in groups of up to six people |  |  |  |  |  |
| F3_6 When asked, provide your contact details to a business so that you can be contacted as needed by the NHS Test and Trace programme |  |  |  |  |  |
| F3_7 Wear a face covering in indoor public settings. |  |  |  |  |  |
| F3_8 Wash your hands with soap and water often, for at least 20 seconds |  |  |  |  |  |
| F3_9 If you do go out, staying 2m apart from other people at all times |  |  |  |  |  |
|  | **Not at all** | **Occasionally** | **Some of the time** | **Most of the time** | **All of the time** |
| F2 Overall, how much have you followed guidance from the government about COVID-19? |  |  |  |  |  |
| F3 How much will you follow *future* guidance from the government about COVID-19? |  |  |  |  |  |

**F4.** Please indicate for each one if you would:

| F4_1 Take a COVID-19 diagnostic test (to test if you currently have COVID-19) if offered? |  | Definitely  Probably  Possibly  Probably not  Definitely not  Don’t know |
| --- | --- | --- |
| F4_2 Take a COVID-19 antibody test (to test if you have previously had COVID-19) if offered |  | Definitely  Probably  Possibly  Probably not  Definitely not  Don’t know |

# G1. Questions about doctors and vaccine developers

For each statement, please indicate how much you disagree or agree:

|  | Disagree completely | Disagree | Agree | Agree completely | Don’t know |
| --- | --- | --- | --- | --- | --- |
| Doctors… |  |  |  |  |  |
| G1_1 I trust doctors |  |  |  |  |  |
| G1_2 They do not really care about me |  |  |  |  |  |
| G1_3 They have my best interests at heart |  |  |  |  |  |
| G1_4 They look down on me |  |  |  |  |  |
| G1_5 They have little respect for me |  |  |  |  |  |
| G1_6 They want to do their best |  |  |  |  |  |
| G1_7 They have no idea what my life is like |  |  |  |  |  |
| G1_8 They often make mistakes |  |  |  |  |  |
| G1_9 They are in it for the money |  |  |  |  |  |
| G1_10 They would give me a vaccine even if it was bad for me |  |  |  |  |  |
| G1_11 I do not trust doctors |  |  |  |  |  |
| COVID-19 vaccine developers… |  |  |  |  |  |
| G1_12 They put safety first |  |  |  |  |  |
| G1_13 I do not trust the vaccine developers |  |  |  |  |  |
| G1_14 They just want to make money |  |  |  |  |  |
| G1_15 They do not care about helping people |  |  |  |  |  |
| G1_16 They do not properly check that the vaccine is safe |  |  |  |  |  |

# H1. Attitudes to doctors and Medicine Questionnaire

For each statement, please indicate how much you disagree or agree:

|  | Strongly disagree | Disagree | Tend to disagree | Tend to agree | Agree | Strongly agree |
| --- | --- | --- | --- | --- | --- | --- |
| H1_1 All doctors are good doctors |  |  |  |  |  |  |
| H1_2. I have absolute faith in all hospital doctors |  |  |  |  |  |  |
| H1_3. No matter how long you have to wait to see a doctor, its worth it |  |  |  |  |  |  |
| H1_4. Doctors know what is best for you |  |  |  |  |  |  |
| H1_5. I only consult a doctor if I’m at death’s door |  |  |  |  |  |  |
| H1_6. Doctors blame their patients if their treatment doesn’t work |  |  |  |  |  |  |
| H1_7. No two doctors will agree with what is wrong with someone |  |  |  |  |  |  |
| H1_8. Doctors are too ready to solve patients’ problems by prescribing tranquilisers |  |  |  |  |  |  |
| H1_9. Doctors are important in keeping us healthy |  |  |  |  |  |  |
| H1_10. I don’t like medical people |  |  |  |  |  |  |
| H1_11. Medicine is based on scientific principles |  |  |  |  |  |  |
| H1_12. The improved health of the nation is due to effective medicine |  |  |  |  |  |  |
| H1_13. Medicine has cures for most diseases |  |  |  |  |  |  |
| H1_14. Medicine is the best profession a person can have |  |  |  |  |  |  |
| H1_15. Medicines can do as much harm as good |  |  |  |  |  |  |
| H1_16. The advice of doctors is mainly common sense |  |  |  |  |  |  |
| H1_17. Many medicines are just placebos or sugar pills |  |  |  |  |  |  |
| H1_18. Often the only purpose of tests is to make a doctor feel less anxious |  |  |  |  |  |  |
| H1_19. Most tests and investigations are done routinely rather than for a particular purpose |  |  |  |  |  |  |

# I. Views about self (The MacArthur Scale of Subjective Social Status)


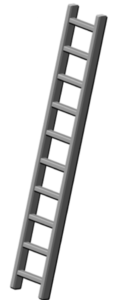
At the top of the ladder are the people who are the best off—those who have the most money, the most education and the most respected jobs.

At the bottom are the people who are the worst off—who have the least money, least education, and the least respected jobs or no job.

The higher up you are on the ladder, the closer you are to the people at the very top; the lower you are, the closer you are to the people at the very bottom.

**I1 place a large “X” on the rung (0 at the bottom, to 10 at the top) where you think you stand at this time in your life, relative to other people in the UK.**


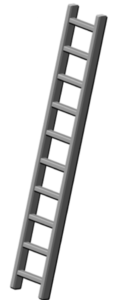


People define community in different ways; please define it in whatever way is most meaningful for you.

At the top of the ladder are the people who have the highest standing in their community.

At the bottom are the people who have the lowest standing in their community.

**I2 Please place a large “X” on the rung (0 at the bottom, to 10 at the top) where you think you stand at this time in your life, relative to other people in your community”.**

# J1. Beliefs about self (The Brief Core Schema Scales (BCSS))

This questionnaire lists beliefs that people can hold about themselves. Please indicate whether you hold each belief by selecting a number from 0-4.

Try to judge the beliefs on how you have viewed yourself **over the past week.**

|  | | Do not believe | Believe it slightly | | Believe it moderately | | Believe it very much | | Believe it totally | | |
| --- | --- | --- | --- | --- | --- | --- | --- | --- | --- | --- | --- |
| J1_1 I am unloved | 0 | | | 1 | | 2 | | 3 | | 4 |  |
| J1_2 I am worthless | 0 | | | 1 | | 2 | | 3 | | 4 |  |
| J1_3 I am weak | 0 | | | 1 | | 2 | | 3 | | 4 |  |
| J1_4 I am vulnerable | 0 | | | 1 | | 2 | | 3 | | 4 |  |
| J1_5 I am bad | 0 | | | 1 | | 2 | | 3 | | 4 |  |
| J1_6 I am a failure | 0 | | | 1 | | 2 | | 3 | | 4 |  |
| J1_7 I am respected | 0 | | | 1 | | 2 | | 3 | | 4 |  |
| J1_8 I am valuable | 0 | | | 1 | | 2 | | 3 | | 4 |  |
| J1_9 I am talented | 0 | | | 1 | | 2 | | 3 | | 4 |  |
| J1_10 I am successful | 0 | | | 1 | | 2 | | 3 | | 4 |  |
| J1_11 I am good | 0 | | | 1 | | 2 | | 3 | | 4 |  |
| J1_12 I am interesting | 0 | | | 1 | | 2 | | 3 | | 4 |  |

# K. Experiences with healthcare

# (General Practice Assessment Questionnaire)

We are interested in your typical experiences of your doctor. How good is your GP at:

|  | Very good | Good | Satisfactory | Poor | Very poor | Does not apply |
| --- | --- | --- | --- | --- | --- | --- |
| K1_1 Putting you at ease? |  |  |  |  |  |  |
| K1_2 Being polite and considerate? |  |  |  |  |  |  |
| K1_3 Listening to you? |  |  |  |  |  |  |
| K1_4. Giving you enough time? |  |  |  |  |  |  |
| K1_5. Assessing your medical condition? |  |  |  |  |  |  |
| K1_6 Explaining your condition and treatment? |  |  |  |  |  |  |
| K1_7 Involving you in decisions about your care? |  |  |  |  |  |  |
| K1_8 Providing or arranging treatment for you? |  |  |  |  |  |  |

K2. How likely are you to recommend your GP surgery to friends and family if they need similar care or treatment?

| Extremely likely | Likely | Neither likely nor unlikely | Unlikely | Extremely unlikely | Don’t know |
| --- | --- | --- | --- | --- | --- |
|  |  |  |  |  |  |

K3. We are interested in your typical experiences of the NHS.

| Have any of the following occurred: | No | Maybe | Yes | Does not apply |
| --- | --- | --- | --- | --- |
| K3_1 Received excellent care |  |  |  |  |
| K3_2 Been put at the back of the queue for help |  |  |  |  |
| K3_3 Staff have gone out of their way to help |  |  |  |  |
| K3_4 Received harmful care |  |  |  |  |
| K3_5 Family received harmful care |  |  |  |  |

| K3x2 We are interested in your typical experiences of the NHS. |  | | |
| --- | --- | --- | --- |
| Do you think: | **No** | **Maybe** | **Yes** |
| K3x2_1 Your community is looked after well by the NHS |  |  |  |
| K3x2_2 The health concerns of your community are neglected by the NHS |  |  |  |
| K3x2_3 The NHS treats your community badly. |  |  |  |

# L. Broad explanations about the COVID-19 virus

For each statement, please indicate how much you disagree or agree:

|  | Do not agree | Agree a little | Agree moderately | Agree a lot | Agree completely | Don’t know |
| --- | --- | --- | --- | --- | --- | --- |
| L1_1. The virus is a hoax. |  |  |  |  |  |  |
| L1_2. The virus is manmade. |  |  |  |  |  |  |
| L1_3. The spread of the virus is a deliberate attempt to reduce the size of the global population. |  |  |  |  |  |  |
| L1_4. The spread of the virus is a deliberate attempt by governments to gain political control. |  |  |  |  |  |  |
| L1_5. The spread of the virus is a deliberate attempt by a group of powerful people to make money. |  |  |  |  |  |  |
| L1_6. The spread of the virus is a deliberate attempt by one nation to destabilize another. |  |  |  |  |  |  |
| L1_7. The spread of the virus is a deliberate attempt by global companies to take control. |  |  |  |  |  |  |

|  | Do not agree | Agree a little | Agree moderately | Agree a lot | Agree completely | Don’t know |
| --- | --- | --- | --- | --- | --- | --- |
| L1x2_8. COVID-19 is a bioweapon developed by China to destroy the West. |  |  |  |  |  |  |
| L1x2_9. The virus is a biological weapon manufactured by the United States. |  |  |  |  |  |  |
| L1x2_10. The United Nations (UN) and World Health Organisation (WHO) have manufactured the virus to take global control. |  |  |  |  |  |  |
| L1x2_11. Jews have created the virus to collapse the economy for financial gain. |  |  |  |  |  |  |
| L1x2_12. The elite have created the virus in order to establish a one-world government. |  |  |  |  |  |  |
| L1 x2_13. Bill Gates has created the virus in order to reduce the world population. |  |  |  |  |  |  |
| L1 x2_14. Big Pharma created COVID-19 to profit from the vaccines. |  |  |  |  |  |  |
| L1 x3_1. COVID-19 is being used by the government to implement a police state. |  |  |  |  |  |  |
| L1 x3_2. COVID-19 is caused by 5G and is a form of radiation poisoning transmitted through radio waves. |  |  |  |  |  |  |
| L1 x3_3. The virus is a smokescreen for a global conspiracy that swapped the real world with a simulation. |  |  |  |  |  |  |
| L1_x3_4 .COVID-19 was created to force everyone to get vaccinated. |  |  |  |  |  |  |
| L1_x3_5. The vaccine will be used to carry out mass sterilisation. |  |  |  |  |  |  |
| L1_x3_6. The World Health Organisation (WHO) already has a vaccine and are withholding it. |  |  |  |  |  |  |
| L1_x3_7. Antibody testing is a plot to harvest our DNA. |  |  |  |  |  |  |

# L. Specific explanations about the COVID-19 virus

# M. Statements about the world (Conspiracy Mentality Questionnaire)

Please rate each of the following statements according to the scale below:

| **0%**  **Certainly Not** | **10% Extremely Unlikely** | **20%**  **Very Unlikely** | **30% Unlikely** | **40%**  **Somewhat Unlikely** | **50%**  **Undecided** | **60%**  **Somewhat Likely** | **70%**  **Likely** | **80% Very Likely** | **90%**  **Extremely Likely** | **100%**  **Certain** |
| --- | --- | --- | --- | --- | --- | --- | --- | --- | --- | --- |

**M1 I think that…**

| M1_1… many very important things happen in the world, which the public is never informed about. | 0% | 100% |
| --- | --- | --- |
| M1_2… politicians usually do not tell us the true motives for their decisions. | 0% | 100% |
| M1_3… government agencies closely monitor all citizens. | 0% | 100% |
| M1_4 … events which superficially seem to lack a connection are often the result of secret activities. | 0% | 100% |
| M1_5… there are secret organizations that greatly influence political decisions. | 0% | 100% |

# N. Beliefs about vaccinations (Vaccine Conspiracy Beliefs Scale)

These questions relate to vaccines in general. They are not specific to COVID-19. For each statement, please indicate how much you disagree or agree:

|  | Strongly Disagree | Disagree | Somewhat Disagree | Neutral | Somewhat Agree | Agree | Strongly Agree |
| --- | --- | --- | --- | --- | --- | --- | --- |
| N1_1 Vaccine safety data is often fabricated (made up). | 1 | 2 | 3 | 4 | 5 | 6 | 7 |
| N1_2 Immunizing children is harmful and this fact is covered up. | 1 | 2 | 3 | 4 | 5 | 6 | 7 |
| N1_3 Pharmaceutical companies cover up the dangers of vaccines. | 1 | 2 | 3 | 4 | 5 | 6 | 7 |
| N1_4 People are deceived about the effectiveness of vaccines. | 1 | 2 | 3 | 4 | 5 | 6 | 7 |
| N1_5 Vaccine effectiveness data is often fabricated (made up). | 1 | 2 | 3 | 4 | 5 | 6 | 7 |
| N1_6 People are deceived about vaccine safety. | 1 | 2 | 3 | 4 | 5 | 6 | 7 |
| N1_7 The government is trying to cover up the link between vaccines and autism. | 1 | 2 | 3 | 4 | 5 | 6 | 7 |

# O. Everyday experiences scale (Everyday Discrimination Scale)

In your day to day life, how often do any of the following things happen to you?

|  | Almost everyday | At least once a week | A few times a month | A few times a year | Less than once a year | Never |
| --- | --- | --- | --- | --- | --- | --- |
| O1_1. You are treated with less courtesy than other people are. |  |  |  |  |  |  |
| O1_2. You are treated with less respect than other people are. |  |  |  |  |  |  |
| O1_3. You receive poorer service than other people at restaurants or shops. |  |  |  |  |  |  |
| O1_4. People act as though they think you are not intelligent. |  |  |  |  |  |  |
| O1x2_1. People act as if they are afraid of you. |  |  |  |  |  |  |
| O1x2_2. People act as if they think you are dishonest. |  |  |  |  |  |  |
| O1x2_3. People act as if they are better than you are. |  |  |  |  |  |  |
| O1x2_4. You are called names or insulted. |  |  |  |  |  |  |
| O1x2_5. You are threatened or harassed. |  |  |  |  |  |  |

# P. Questions about anger (Dimensions of Anger Reactions-5)

Thinking over the ***past 4 weeks****,* choose the number that best describes the amount of time you felt that way.

|  | | None or almost  none of the time | A little  of the time | Some of  the time | Most of  the time | All or almost  all of the time |
| --- | --- | --- | --- | --- | --- | --- |
| P1_1 | I found myself getting angry at people or situations. | 1 | 2 | 3 | 4 | 5 |
| P1_2 | When I got angry, I got really mad. | 1 | 2 | 3 | 4 | 5 |
| P1_3 | When I got angry, I stayed angry. | 1 | 2 | 3 | 4 | 5 |
| P1_4 | When I got angry at someone, I wanted to hit them. | 1 | 2 | 3 | 4 | 5 |
| P1_5 | My anger prevented me from getting along with people as well as I'd have liked to. | 1 | 2 | 3 | 4 | 5 |

# Q. Worldviews (A “need for chaos”)

For each statement, please indicate how much you disagree or agree:

|  | Strongly disagree | Disagree | Somewhat disagree | Neither agree nor disagree | Somewhat agree | Agree | Strongly agree |
| --- | --- | --- | --- | --- | --- | --- | --- |
| Q1_1. I get a kick when natural disasters strike in foreign countries |  |  |  |  |  |  |  |
| Q1_2. Our social institutions are rotten to the core |  |  |  |  |  |  |  |
| Q1_3. I fantasize about a natural disaster wiping out most of humanity such that a small group of people can start all over |  |  |  |  |  |  |  |
| Q1_4. I think society should be burned to the ground |  |  |  |  |  |  |  |
| Q1_5. When I think about our political and social institutions, I cannot help thinking ‘just let them all burn’ |  |  |  |  |  |  |  |
| Q1_6. We cannot fix the problems in our social institutions, we need to tear them down and start over |  |  |  |  |  |  |  |
| Q1_7. We need to tear down the current political institutions and start all over |  |  |  |  |  |  |  |
| Q1_8. I prepare for a time when the military, police and the state no longer can protect me |  |  |  |  |  |  |  |
| Q1_9. I need chaos around me – it is too boring if nothing is going on |  |  |  |  |  |  |  |
| Q1_10. Sometimes I just feel like destroying beautiful things |  |  |  |  |  |  |  |
| Q1_11. There is no right and wrong in the world |  |  |  |  |  |  |  |

# R. Views on society (Lifestyle and Economic Government Liberty Scale)

For each statement, please indicate how much you disagree or agree:

|  | Strongly Disagree | Moderately Disagree | Slightly Disagree | Slightly Agree | Moderately Agree | Strongly Agree |
| --- | --- | --- | --- | --- | --- | --- |
| R1_1 People who are successful in business have a right to enjoy their wealth as they see fit. |  |  |  |  |  |  |
| R1_2 Society works best when it lets individuals take responsibility for their own lives without telling them what to do. |  |  |  |  |  |  |
| R1_3 The government interferes far too much in our everyday lives. |  |  |  |  |  |  |
| R1_4 The government should do more to advance the common good, even if that means limiting the freedom and choices of individuals. |  |  |  |  |  |  |
| R1_5 Property owners should be allowed to develop their land or build their homes in any way they choose, as long as they don't endanger their neighbors. |  |  |  |  |  |  |
| R1_6 I think everyone should be free to do as they choose, so long as they don't infringe upon the equal freedom of others. |  |  |  |  |  |  |
| R1_7 People should be free to decide what group norms or traditions they themselves want to follow. |  |  |  |  |  |  |

|  | Strongly Disagree | Moderately Disagree | Slightly Disagree | Slightly Agree | Moderately Agree | Strongly Agree |
| --- | --- | --- | --- | --- | --- | --- |
| R1_8 The politicians in the UK Parliament need to follow the will of the people. |  |  |  |  |  |  |
| R1_9 The people, and not politicians, should make our most important policy decisions. |  |  |  |  |  |  |
| R1_10 I would rather be represented by a citizen than by a specialized politician. |  |  |  |  |  |  |
| R1_11 Elected officials talk too much and take too little action. |  |  |  |  |  |  |
| R1_12 What people call “compromise” in politics is really just selling out on one’s principles. |  |  |  |  |  |  |

# (Very Short Authoritarian Scale)

For each statement, please indicate how much you disagree or agree:

|  | Very strongly disagree  1 | Strongly disagree  2 | Moderately disagree  3 | Slightly disagree  4 | Neutral  5 | Slightly agree  6 | Moderately Agree  7 | Strongly agree  8 | Very strongly agree  9 |
| --- | --- | --- | --- | --- | --- | --- | --- | --- | --- |
| R2_1 It’s great that many young people today are prepared to defy authority. |  |  |  |  |  |  |  |  |  |
| R2_2 What our country needs most is discipline, with everyone following our leaders in unity. |  |  |  |  |  |  |  |  |  |
| R2_3 God’s laws about abortion, pornography, and marriage must be strictly followed before it is too late. |  |  |  |  |  |  |  |  |  |
| R2_4 There is nothing wrong with premarital sexual intercourse. |  |  |  |  |  |  |  |  |  |
| R2_5 Our society does NOT need tougher government and stricter laws. |  |  |  |  |  |  |  |  |  |
| R2_6 The facts on crime and the recent public disorders show we have to crack down harder on troublemakers, if we are going to preserve law and order. |  |  |  |  |  |  |  |  |  |

# S. Religious beliefs and health

For each statement, please indicate how much you disagree or agree:

|  | Strongly disagree | Disagree | Agree | Strongly agree |
| --- | --- | --- | --- | --- |
| S1_1 I tend to avoid things harmful to my body because of my religious/spiritual beliefs. |  |  |  |  |
| S1_2 Religious/spiritual beliefs have great influence on my health. |  |  |  |  |
| S1_3 God helps me to maintain a healthy lifestyle. |  |  |  |  |
| S1_4 God helps me to avoid bad health habits. |  |  |  |  |
| S1_5 Because of my religious/spiritual beliefs, I do not put harmful substances into my body. |  |  |  |  |
| S1_6 I try to engage in “clean living” because this is what my religion/spirituality teaches. |  |  |  |  |
| S1_7 Because of my religious/spiritual beliefs, I abstain from tobacco/smoking. |  |  |  |  |

# S. Illness as a Punishment for sin

|  | Strongly disagree | Disagree | Agree | Strongly agree |
| --- | --- | --- | --- | --- |
| S1_8 Illness is the result of one’s negative thoughts. |  |  |  |  |
| S1_9 God uses sickness to send a message to people. |  |  |  |  |
| S1_10 Illness comes because of something bad a person has done in their life. |  |  |  |  |
| S1_11 God sometimes uses physical illnesses to punish people. |  |  |  |  |
| S1_12 God uses sickness as punishment for the things people have done wrong in their lives. |  |  |  |  |
| S1_13 Illness is a punishment/result from God for sinful behaviours or lifestyle. |  |  |  |  |
| S1_14 Illness is caused by a sinful lifestyle. |  |  |  |  |
| S1_15 God uses sickness as a way to punish people for their sins. |  |  |  |  |
