# Supplementary material for: COVID-19 vaccine hesitancy in the UK: the Oxford coronavirus explanations, attitudes, and narratives survey (Oceans) II
Source: Psychol Med. 2020 Dec 11:1–15. doi: 10.1017/S0033291720005188 (PMC7804077; doi:10.1017/S0033291720005188)
Supplement: Supplementary file 1 [file S0033291720005188sup.zip › S0033291720005188sup002.docx]

**Supplementary materials: Statistical analysis**

**Survey details**

An online survey with a quota sampled UK participant group of 5,114 adults (18+ years old) was conducted from 24th September to the 17th October 2020 via a market research company (Lucid; https://luc.id/). The quotas used were based upon UK Office for National Statistics (ONS) population estimate data for gender, age, ethnicity, income, and region. Lucid’s platform serves as a centralised source for survey responses, working with over 250 survey suppliers. Lucid operate a marketplace in which they advertise the survey to suppliers, who provide individual participants, with sampling by Lucid from this pool. The advantage of using multiple survey sources is substantially less reliance on any particular demographic or segment of the population. Respondents will have been sourced from: ads and promotions across digital networks, search, word of mouth and membership referrals, social networks, online and mobile games, affiliate marketing, banner ads, offerwalls, TV and radio ads, and offline recruitment with mail campaigns. Participants have opted in to being a panel member for the supplier as well as providing informed consent to this particular survey. Invited respondents did not know the topic of the survey before saying that they would complete it. They were simply told that there was a new survey and informed of the time period for it to be completed. Only after agreeing to participate did they see the online introduction. There were 6,214 complete responses. Respondents were excluded before analysis (n=1100) if they did not meet the inclusion criteria (under 18 or did not consent), responded to all questions in the same way (or straight-lined all assessments in differing ways), or had a completion time lower than nine minutes. The average time to complete the survey was 36.5 (SD=59.0) minutes.

The Oxford Coronavirus Explanations, Attitudes, and Narratives Survey II (OCEANSII) was approved by the University of Oxford Central University Research Ethics Committee ((R71830/RE001) and all participants provided informed consent. Participants were given the following rationale for OCEANSII: “Medical researchers are developing and testing potential vaccinations for coronavirus. A vaccination against coronavirus may become available in the near future. We want to learn about people’s views about a coronavirus vaccination. In particular, we want to find out how many people would or would not wish to be vaccinated and the reasons behind their decision.”

**SEM analysis methods**

Structural equation modelling (SEM) was used to assess two explanatory models. The first was between beliefs about a COVID-19 vaccine and hesitancy, and the second was between the broader psychological constructs of mistrust and vaccine hesitancy. SEM is a collection of statistical techniques composed of two parts. The first part is the confirmatory measurement model, also known as the confirmation factor analysis (CFA), which estimates the relations among latent constructs and their observed indicators, and the second part is the structural model, which estimates the relations among constructs (Kline, 2015). This combination allows the elimination of unreliability of measurement in the models and can be used to make assumptions about how latent constructs derived from observed variables are associated with each other. The lavaan R package was used to conduct the SEM analysis (Rosseel, 2012).

First, we assessed the measurement model based on factor loadings, inter-correlation between factors, and several goodness-of-fit indices, to ensure appropriate specification. Second, we then evaluated the structural model. With non-normal distributions and missing values within the data, which in this case is considered either missing completely at random (MCAR) or missing at random (MAR) due to test design (Rubin, 1976; Little & Rubin, 1986), we utilised the full information maximum likelihood (FIML) estimation procedure with robust (Huber-White) standard errors and a scaled test statistic to estimate the SEM models. Previous research has indicated that using the FIML is more efficient and involves less bias than other ad hoc missing data techniques (Enders, 2001; Enders & Bandalos, 2001). The data are supported by the SEM model when the root mean square error of approximate (RMSEA) and standardized root mean square residual (SRMR) are less than 0.08 (Kline, 2005). Additional fit indices included the comparative fit index (CFI) and Tucker-Lewis Index (TLI), all of which should exceed 0.90 (Kline, 2010), with RMSEA < 0.06, and CFI and TLI > 0.95 indicating good model data fit (Hu & Bentler, 1999).

The predictors in the SEM models consisted of both new and existing measures. We conducted EFA and CFA on new measures (with the data randomly separated into two equal sizes), and CFA on existing measures to evaluate their factorial structure (for full details see the supplementary materials). We examined fit indices for the models independently and applied the criteria described above when judging data-model adequacy. Only items that are reflective of the respective latent factor based on acceptable model fit and adequate factor loadings are used in the final SEM model. Factor loadings with values less than 0.40 were removed from the respective models. The factor loadings and goodness of fit indices for each measurement model are reported in full in the supplementary materials. Modification indices (MIs) were also inspected during the analysis of each measure and four pairs of items from four different measures (Vaccination Knowledge Scale; NHS Experience Questionnaire; Everyday Discrimination Scale; Dimensions of Anger Reactions-5) had within-factor correlated residuals, which were carried forward to the SEM model to isolate sources of poor fit. We only allowed for within-factor correlated residuals, which was considered a reasonable modification as this likely represented non-random error within the factor (Gerbing & Anderson, 1984).

In assessing the relations among constructs in the structural model, we employed the backward elimination procedure to reduce multicollinearity issues (Hocking, 1976; Grewal et al 2004). We fixed the direct effects of the variables to zero in the structural model for those with p-values above 0.05. The aim was to determine the most representative subset of predictors that ensured accurate predictions. Thus, it does not mean that the dropped predictors are unrelated to the predicted variable, but rather, that they do not provide additional explanatory effect beyond those predictors in the model. The least significant path is first fixed to zero from the structural model (highest p value) and the SEM is refitted, and the p values are inspected again. This process is repeated until all predictors in the model are significant at the cut-off threshold. The Akaike Information Criteria (AIC) and Bayesian Information Criteria (BIC) were used to assess model fit between the full and reduced models, with lower values indicating better fitting models relative to the ones in comparison (Akaike, 1987; Schwarz, 1978).

It is fairly common for measurement models in the medical sciences to have latent factors that are highly related to each other. However, regression weights only show the additional effect of a predictor on top of the others and do not reveal their shared effects. Thus, when the number of predictors increases, it becomes likely that predictors will be highly collinear, which makes the regression weights unstable. This situation is commonly known as the ‘bouncing beta problem’ (Kiers & Similde, 2007). Thus, to overcome this issue, we conducted a series of procedures to determine if specific predictors were functioning as suppressor variables (Knowlden, 2014; Paulhus et al, 2004). The first procedure involved examination of the correlation matrix to determine the relationship between the hypothesized suppressors, the other independent variables in the model, and the outcome variable. Suppressors are variables that have a minimal relation to the dependent variable, while being significantly correlated to the other independent variables in the model (Lancaster, 1999). The next procedure was the inspection of the sign of the suppressor’s beta weight relative to the correlation with the dependent variable, which may indicate the type of suppressor (Watson et al 2013). The final procedure tested the possibility of multicollinearity through the examination of the variance inflation factor (VIF) and tolerance values. VIF values greater than 5.0 and tolerance values of less than 0.1 are generally accepted cut-offs for indicating the presence of multicollinearity (Walker, 2003; Afifi et al 2003).

**Description of potential suppressors**

Further review of the beta estimates suggested that several predictors may be acting as suppressors within the model. Thus, we used the specified series of procedures to identify potential suppressors. We first inspected the factor correlations of SEM model 5 (see Table S6 in supplementary materials) and identified that five predictors had near zero or weak correlations with vaccine hesitancy (negative self r = 0.06; anger r = 0.11; illness as punishment r = 0.13; others disrespectful r = -0.10; positive self r = -0.03); yet, four of these predictors had substantial correlations with mistrust (negative self r = 0.50; anger r = 0.58; illness as punishment r = 0.64; others disrespectful r = -0.44) and positive self had substantial correlations with subjective sense of social status in UK (r = -0.41) and subjective sense of social status in community (r = -0.44). We also found that the beta weights of these five variables were in the opposite direction of the factor correlations (negative self b = - 0.187; anger b = - 0.174; illness as punishment b = -0.333; others disrespect b = 0.074; positive self = 0.071), implying the status of a cross-over suppression (Watson et al, 2013).

**Key:**

IMP = collective importance; SPD = speed of development; WRK = vaccine will be effective; S.EF = side effects; VAC.HES = vaccine hesitancy; CVK = Knowledge about childhood vaccinations; GEN.K = general knowledge about vaccines; DIS.DOC = interpersonal disrespect from doctors; NEG.VD = negative views of vaccine developers; NHS.NEG= negative experiences of NHS care; CHAOS = need for chaos; C19.CON = coronavirus general conspiracy beliefs; VAC.CON = vaccination conspiracy beliefs; RES.DOC = respect from doctors; POS.DOC (or POS.ATT) = positive attitudes to doctors; NHS.POS = positive NHS experiences; POS.MED = positive attitudes to medication; GP.POS = positive GP experiences; MISTRUST = higher order excessive mistrust factor; +VE HC = higher order positive healthcare experiences factor; SLF.COM = subjective sense of social status in community; SLF.UK = subjective sense of social status in UK; VAC.HES = vaccine hesitancy; ILL.PUN = Illness as punishment by God for sin; REL.H = perceived religious influence on health behaviour; POP=populist beliefs; LIB=libertarian beliefs; ANGER = anger; POS.SELF= positive self beliefs; NEG.SELF= negative self beliefs

**Model 1: Beliefs about a COVID-19 vaccine**

IMPT=collective importance; SPV=Speed of development; WRKS=efficacy, SEF=side effects

| Table S1. Factor correlations of CFA model 1 | | | | | | | |
| --- | --- | --- | --- | --- | --- | --- | --- |
| Constructs | IMPT | | SPV | | WRKS | SEF | |
| IMPT | 1.00 | |  | |  |  | |
| SPV | 0.84 | | 1.00 | |  |  | |
| WRKS | 0.86 | | 0.81 | | 1.00 |  | |
| SEF | 0.92 | | 0.79 | | 0.81 | 1.00 | |
|  | | | | | | | |
| Table S2. Factor loadings of CFA Model 1 | | | | | | |  |
| Items | | Factors | | Factor loadings | | |  |
| B20 | | IMPT | | 0.87 | | |  |
| B21 | |  |  | 0.87 | | |  |
| B24 | |  |  | 0.68 | | |  |
| B28 | |  |  | 0.81 | | |  |
| B32 | |  |  | 0.82 | | |  |
| B22 | | SPV | | 0.89 | | |  |
| B23 | |  |  | 0.90 | | |  |
| B33 | |  |  | 0.73 | | |  |
| B16 | | WRK | | 0.45 | | |  |
| B17 | |  |  | 0.88 | | |  |
| B18 | |  |  | 0.90 | | |  |
| B26 | | SEF | | 0.67 | | |  |
| B27 | |  |  | 0.77 | | |  |
| B34 | |  |  | 0.76 | | |  |
| SEF | | BELIEFS | | 0.94 | | |  |
| IMPT | |  |  | 0.99 | | |  |
| SDV | |  |  | 0.86 | | |  |
| WRK | |  |  | 0.87 | | |  |
| B1 | | VACCINE HESITANCY | | 0.92 | | |  |
| B2 | |  |  | 0.91 | | |  |
| B3 | |  |  | 0.91 | | |  |
| B5 | |  |  | 0.91 | | |  |
| B6 | |  |  | 0.88 | | |  |
| B11 | |  |  | 0.92 | | |  |
| B12 | |  |  | 0.90 | | |  |
|  | | | | | | |  |

**Model 2: Mistrust**

| Table S3. Factor correlations in CFA model with higher order factors | | | | | | | | | | | | |
| --- | --- | --- | --- | --- | --- | --- | --- | --- | --- | --- | --- | --- |
| Constructs | CVK | GEN.K | DIS.DOC | RES.DOC | NEG.VD | POS.ATT | POS.MED | NEG.SLF | POS.SELF | GP.POS | NHS.POS | NHS.NEG |
| CVK | 1 | NA | NA | NA | NA | NA | NA | NA | NA | NA | NA | NA |
| GEN.K | 0.51 | 1 | NA | NA | NA | NA | NA | NA | NA | NA | NA | NA |
| DIS.DOC | 0.52 | 0.63 | 1 | NA | NA | NA | NA | NA | NA | NA | NA | NA |
| RES.DOC | -0.30 | -0.40 | -0.41 | 1 | NA | NA | NA | NA | NA | NA | NA | NA |
| NEG.VD | 0.52 | 0.63 | 0.64 | -0.41 | 1 | NA | NA | NA | NA | NA | NA | NA |
| POS.ATT | -0.29 | -0.38 | -0.39 | 0.72 | -0.38 | 1 | NA | NA | NA | NA | NA | NA |
| POS.MED | -0.26 | -0.34 | -0.35 | 0.65 | -0.35 | 0.61 | 1 | NA | NA | NA | NA | NA |
| NEG.SLF | 0.19 | 0.40 | 0.41 | -0.20 | 0.41 | -0.19 | -0.17 | 1 | NA | NA | NA | NA |
| POS.SELF | 0.02 | 0.06 | 0.06 | 0.20 | 0.06 | 0.19 | 0.17 | -0.23 | 1 | NA | NA | NA |
| GP.POS | -0.25 | -0.33 | -0.34 | 0.62 | -0.33 | 0.59 | 0.53 | -0.16 | 0.16 | 1 | NA | NA |
| NHS.POS | -0.28 | -0.37 | -0.38 | 0.70 | -0.37 | 0.66 | 0.59 | -0.18 | 0.18 | 0.57 | 1 | NA |
| NHS.NEG | 0.48 | 0.58 | 0.59 | -0.37 | 0.58 | -0.35 | -0.32 | 0.37 | 0.05 | -0.3 | -0.34 | 1 |
| C19.CON | 0.49 | 0.59 | 0.61 | -0.38 | 0.60 | -0.36 | -0.32 | 0.38 | 0.05 | -0.31 | -0.35 | 0.55 |
| VAC.CON | 0.53 | 0.64 | 0.66 | -0.42 | 0.66 | -0.39 | -0.35 | 0.42 | 0.06 | -0.34 | -0.38 | 0.6 |
| OTH.DIS | -0.20 | -0.35 | -0.36 | 0.26 | -0.36 | 0.25 | 0.22 | -0.50 | 0.18 | 0.22 | 0.24 | -0.33 |
| OTH.NEG | -0.23 | -0.44 | -0.45 | 0.24 | -0.44 | 0.22 | 0.20 | -0.54 | -0.03 | 0.19 | 0.22 | -0.4 |
| ANGER | 0.26 | 0.47 | 0.48 | -0.19 | 0.47 | -0.18 | -0.16 | 0.53 | 0.00 | -0.16 | -0.18 | 0.43 |
| CHAOS | 0.47 | 0.57 | 0.59 | -0.37 | 0.58 | -0.35 | -0.31 | 0.37 | 0.05 | -0.30 | -0.34 | 0.53 |
| LIB | 0.14 | 0.15 | 0.15 | 0.06 | 0.15 | 0.06 | 0.05 | 0.04 | 0.08 | 0.05 | 0.06 | 0.14 |
| POP | 0.13 | 0.20 | 0.20 | -0.05 | 0.20 | -0.05 | -0.04 | 0.08 | -0.02 | -0.04 | -0.05 | 0.18 |
| REL.H | 0.26 | 0.36 | 0.37 | -0.04 | 0.37 | -0.03 | -0.03 | 0.23 | 0.27 | -0.03 | -0.03 | 0.34 |
| ILL.PUN | 0.34 | 0.51 | 0.52 | -0.11 | 0.52 | -0.11 | -0.10 | 0.36 | 0.23 | -0.09 | -0.10 | 0.47 |
| VAC.HES | 0.43 | 0.39 | 0.40 | -0.43 | 0.39 | -0.41 | -0.37 | 0.07 | -0.08 | -0.36 | -0.40 | 0.36 |
| MISTRUST | 0.65 | 0.79 | 0.80 | -0.51 | 0.80 | -0.48 | -0.43 | 0.51 | 0.07 | -0.42 | -0.47 | 0.73 |
| POS.HEALTHCARE | -0.35 | -0.46 | -0.47 | 0.87 | -0.47 | 0.82 | 0.74 | -0.23 | 0.23 | 0.72 | 0.80 | -0.43 |
|  | | | | | | | | | | | | |

| Cont. | | | | | | | | | | | | | |
| --- | --- | --- | --- | --- | --- | --- | --- | --- | --- | --- | --- | --- | --- |
| Constructs | C19.CON | VAC.CON | OTH.DIS | OTH.NEG | ANGER | CHAOS | LIB | POP | REL.H | ILL.PUN | VAC.HES | MISTRUST | POS.HEALTHCARE |
| CVK | NA | NA | NA | NA | NA | NA | NA | NA | NA | NA | NA | NA | NA |
| GEN.K | NA | NA | NA | NA | NA | NA | NA | NA | NA | NA | NA | NA | NA |
| DIS.DOC | NA | NA | NA | NA | NA | NA | NA | NA | NA | NA | NA | NA | NA |
| RES.DOC | NA | NA | NA | NA | NA | NA | NA | NA | NA | NA | NA | NA | NA |
| NEG.VD | NA | NA | NA | NA | NA | NA | NA | NA | NA | NA | NA | NA | NA |
| POS.ATT | NA | NA | NA | NA | NA | NA | NA | NA | NA | NA | NA | NA | NA |
| POS.MED | NA | NA | NA | NA | NA | NA | NA | NA | NA | NA | NA | NA | NA |
| NEG.SLF | NA | NA | NA | NA | NA | NA | NA | NA | NA | NA | NA | NA | NA |
| POS.SELF | NA | NA | NA | NA | NA | NA | NA | NA | NA | NA | NA | NA | NA |
| GP.POS | NA | NA | NA | NA | NA | NA | NA | NA | NA | NA | NA | NA | NA |
| NHS.POS | NA | NA | NA | NA | NA | NA | NA | NA | NA | NA | NA | NA | NA |
| NHS.NEG | NA | NA | NA | NA | NA | NA | NA | NA | NA | NA | NA | NA | NA |
| C19.CON | 1 | NA | NA | NA | NA | NA | NA | NA | NA | NA | NA | NA | NA |
| VAC.CON | 0.62 | 1 | NA | NA | NA | NA | NA | NA | NA | NA | NA | NA | NA |
| OTH.DIS | -0.34 | -0.37 | 1 | NA | NA | NA | NA | NA | NA | NA | NA | NA | NA |
| OTH.NEG | -0.42 | -0.45 | 0.8 | 1 | NA | NA | NA | NA | NA | NA | NA | NA | NA |
| ANGER | 0.44 | 0.48 | -0.45 | -0.54 | 1 | NA | NA | NA | NA | NA | NA | NA | NA |
| CHAOS | 0.55 | 0.60 | -0.33 | -0.4 | 0.43 | 1 | NA | NA | NA | NA | NA | NA | NA |
| LIB | 0.14 | 0.16 | -0.02 | 0.01 | 0.11 | 0.14 | 1 | NA | NA | NA | NA | NA | NA |
| POP | 0.19 | 0.20 | -0.09 | 0.01 | 0.12 | 0.18 | 0.59 | 1 | NA | NA | NA | NA | NA |
| REL.H | 0.35 | 0.38 | -0.18 | -0.33 | 0.32 | 0.34 | 0.08 | 0.03 | 1 | NA | NA | NA | NA |
| ILL.PUN | 0.49 | 0.53 | -0.30 | -0.50 | 0.49 | 0.47 | 0.08 | 0.03 | 0.76 | 1 | NA | NA | NA |
| VAC.HES | 0.37 | 0.40 | -0.11 | -0.09 | 0.10 | 0.36 | 0.11 | 0.15 | 0.05 | 0.1 | 1 | NA | NA |
| MISTRUST | 0.75 | 0.82 | -0.45 | -0.55 | 0.59 | 0.73 | 0.19 | 0.25 | 0.46 | 0.65 | 0.49 | 1 | NA |
| POS.HEALTHCARE | -0.44 | -0.48 | 0.30 | 0.27 | -0.22 | -0.42 | 0.07 | -0.06 | -0.04 | -0.13 | -0.50 | -0.58 | 1 |
|  | | | | | | | | | | | | | |

| Table S4. Factor loadings of CFA model 2 with higher order factors | | | |
| --- | --- | --- | --- |
| Items | Factors | Factor Loadings |  |
| D1_4 | CVK | 0.61 |  |
| D1_5 |  | 0.63 |  |
| D1_7 |  | 0.74 |  |
| D1_9 |  | 0.69 |  |
| D1_1 | GEN.K | 0.65 |  |
| D1_2 |  | 0.62 |  |
| D1_3 |  | 0.67 |  |
| D1_8 |  | 0.61 |  |
| G1_2 | DIS.DOC | 0.69 |  |
| G1_5 |  | 0.79 |  |
| G1_7 |  | 0.57 |  |
| G1_8 |  | 0.67 |  |
| G1_9 |  | 0.76 |  |
| G1_11 |  | 0.83 |  |
| G1_1 | RES.DOC | 0.78 |  |
| G1_3 |  | 0.78 |  |
| G1_6 |  | 0.75 |  |
| G1_13 | NEG.VD | 0.83 |  |
| G1_14 |  | 0.79 |  |
| G1_15 |  | 0.86 |  |
| G1_16 |  | 0.86 |  |
| H1_1 | POS.ATT | 0.60 |  |
| H1_3 |  | 0.84 |  |
| H1_4 |  | 0.85 |  |
| H1_12 |  | 0.71 |  |
| H1_13 |  | 0.72 |  |
| H1_14 |  | 0.54 |  |
| J1_2 | NEG.SLF | 0.89 |  |
| J1_3 |  | 0.87 |  |
| J1_4 |  | 0.71 |  |
| J1_5 |  | 0.79 |  |
| J1_6 |  | 0.87 |  |
| J1_7 | POS.SLF | 0.74 |  |
| J1_9 |  | 0.83 |  |
| J1_10 |  | 0.84 |  |
| J1_12 |  | 0.77 |  |
| K1_2 | GP.POS | 0.79 |  |
| K1_4 |  | 0.80 |  |
| K1_6 |  | 0.88 |  |
| K1_7 |  | 0.88 |  |
| K1_8 |  | 0.87 |  |
| K3_1 | NHS.POS | 0.66 |  |
| K3_3 |  | 0.57 |  |
| K3x2_1 |  | 0.74 |  |
| K3_2 | NHS.NEG | 0.58 |  |
| K3_4 |  | 0.77 |  |
| K3_5 |  | 0.74 |  |
| K3x2_3 |  | 0.67 |  |
| L1_1 | C19.CON | 0.67 |  |
| L1_3 |  | 0.85 |  |
| L1_4 |  | 0.91 |  |
| L1_5 |  | 0.92 |  |
| L1_6 |  | 0.87 |  |
| L1_7 |  | 0.92 |  |
| N1_1 | VAC.CON | 0.84 |  |
| N1_2 |  | 0.86 |  |
| N1_3 |  | 0.87 |  |
| N1_4 |  | 0.91 |  |
| N1_5 |  | 0.92 |  |
| N1_6 |  | 0.92 |  |
| N1_7 |  | 0.86 |  |
| O1_2 | OTH.DIS | 0.84 |  |
| O1_3 |  | 0.81 |  |
| O1_4 |  | 0.85 |  |
| O1x2_3 |  | 0.79 |  |
| O1x2_1 | OTH.NEG | 0.79 |  |
| O1x2_2 |  | 0.86 |  |
| O1x2_5 |  | 0.80 |  |
| P1_1 | ANGER | 0.74 |  |
| P1_2 |  | 0.88 |  |
| P1_3 |  | 0.90 |  |
| P1_4 |  | 0.80 |  |
| P1_5 |  | 0.79 |  |
| Q1_1 | CHAOS | 0.82 |  |
| Q1_3 |  | 0.84 |  |
| Q1_5 |  | 0.76 |  |
| Q1_9 |  | 0.82 |  |
| Q1_11 |  | 0.65 |  |
| R1_2 | LIB | 0.53 |  |
| R1_5 |  | 0.65 |  |
| R1_6 |  | 0.80 |  |
| R1_7 |  | 0.70 |  |
| R1_8 | POP | 0.60 |  |
| R1_10 |  | 0.70 |  |
| R1_11 |  | 0.79 |  |
| R1_12 |  | 0.77 |  |
| S1_1 | REL.H | 0.79 |  |
| S1_2 |  | 0.86 |  |
| S1_3 |  | 0.86 |  |
| S1_5 |  | 0.88 |  |
| S1_6 |  | 0.88 |  |
| S1_7 |  | 0.85 |  |
| S1_8 | ILL.PUN | 0.78 |  |
| S1_9 |  | 0.88 |  |
| S1_10 |  | 0.88 |  |
| S1_11 |  | 0.91 |  |
| S1_12 |  | 0.92 |  |
| S1_13 |  | 0.91 |  |
| S1_14 |  | 0.87 |  |
| S1_15 |  | 0.91 |  |
| B1 | VACCINE HESITANCY | 0.92 |  |
| B2 |  | 0.92 |  |
| B3 |  | 0.92 |  |
| B5 |  | 0.91 |  |
| B6 |  | 0.87 |  |
| B11 |  | 0.92 |  |
| B12 |  | 0.89 |  |
| DIS.DOC | MISTRUST | 0.81 |  |
| NEG.VD |  | 0.81 |  |
| NHS.NEG |  | 0.73 |  |
| CHAOS |  | 0.72 |  |
| C19.CON |  | 0.76 |  |
| VAC.CON |  | 0.82 |  |
| GEN.K |  | 0.78 |  |
| CVK |  | 0.63 |  |
| RES.DOC | POS.HEALTHCARE | 0.87 |  |
| POS.ATT |  | 0.82 |  |
| NHS.POS |  | 0.80 |  |
| POS.MED |  | 0.74 |  |
| GP.POS |  | 0.71 |  |
|  | | | |

Figure S1. SEM model with two higher order factors


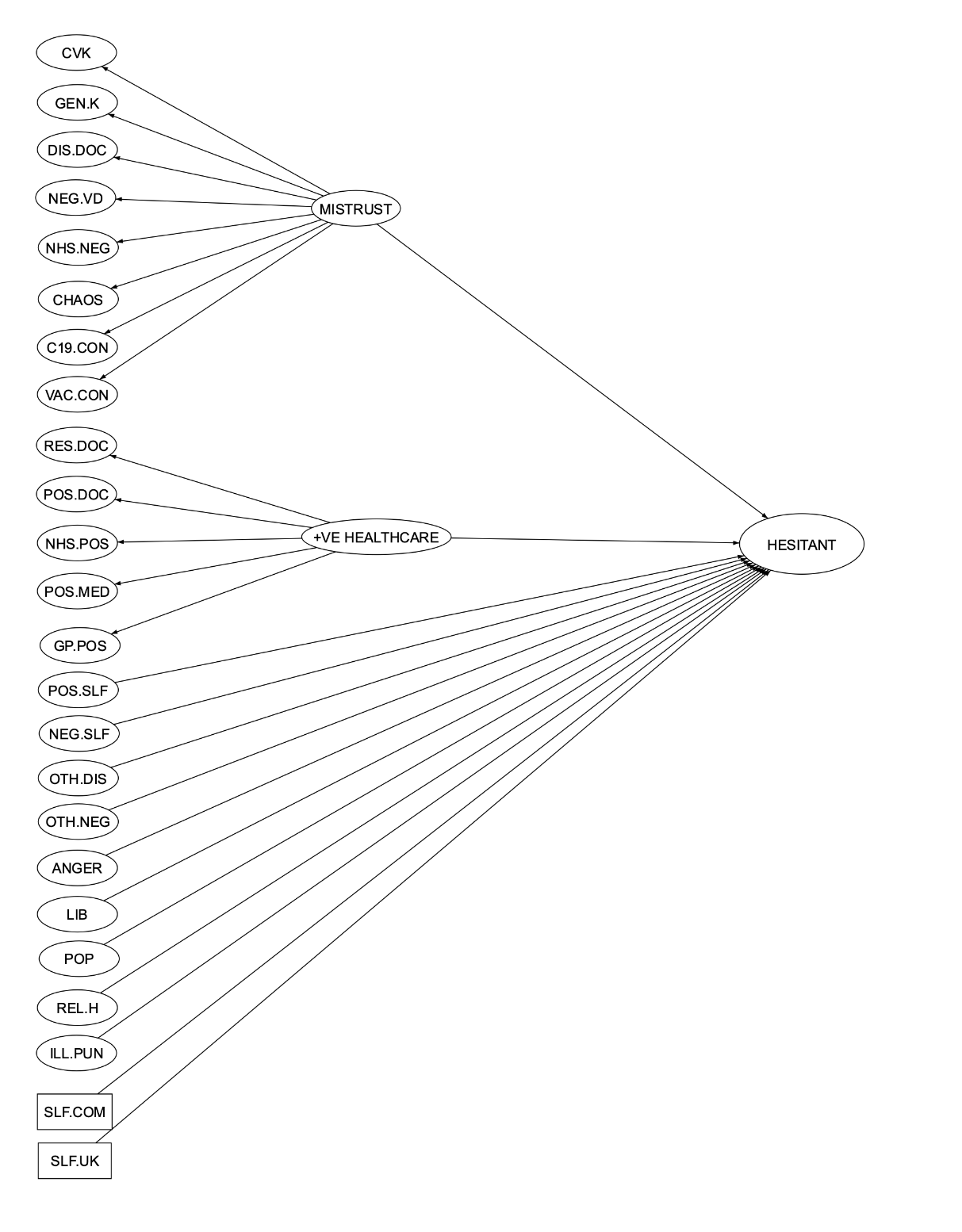


| Table S5. Model Comparisons between nested SEM models | | | |  |  |  |  |  |  |
| --- | --- | --- | --- | --- | --- | --- | --- | --- | --- |
| Model Comparisons | Df | AIC | BIC | | Chisq | Chisq diff | Df diff | Pr(>Chisq) |  |
| SEM Model 1 | 5693 | 1235738 | 1238393 | | 36967 |  |  |  |  |
| SEM Model 2 | 5694 | 1235736 | 1238385 | | 36968 | 0.41 | 1 | 0.52 |  |
| SEM Model 3 | 5695 | 1235736 | 1238378 | | 36970 | 1.49 | 1 | 0.22 |  |
| SEM Model 4 | 5696 | 1235737 | 1238373 | | 36973 | 3.48 | 1 | 0.06 |  |
| SEM Model 5 | 5697 | 1235738 | 1238367 | | 36976 | 2.69 | 1 | 0.10 |  |
|  | | | | | | | | | |

| Table S6. Factor correlations of SEM model | | | | | | | | | | | | | | | | | | | | | | | | |  |  |  |  |
| --- | --- | --- | --- | --- | --- | --- | --- | --- | --- | --- | --- | --- | --- | --- | --- | --- | --- | --- | --- | --- | --- | --- | --- | --- | --- | --- | --- | --- |
| Constructs | CVK | | GEN.K | | DIS.DOC | | RES.DOC | | NEG.VD | | POS.ATT | | | POS.MED | | | NEG.SLF | | POS.SELF | | GP.POS | | NHS.POS | | | NHS.NEG |  |  |
| CVK | 1 | | NA | | NA | | NA | | NA | | NA | | | NA | | | NA | | NA | | NA | | NA | | | NA |  |  |
| GEN.K | 0.49 | | 1 | | NA | | NA | | NA | | NA | | | NA | | | NA | | NA | | NA | | NA | | | NA |  |  |
| DIS.DOC | 0.51 | | 0.63 | | 1 | | NA | | NA | | NA | | | NA | | | NA | | NA | | NA | | NA | | | NA |  |  |
| RES.DOC | -0.32 | | -0.4 | | -0.41 | | 1 | | NA | | NA | | | NA | | | NA | | NA | | NA | | NA | | | NA |  |  |
| NEG.VD | 0.51 | | 0.63 | | 0.66 | | -0.41 | | 1 | | NA | | | NA | | | NA | | NA | | NA | | NA | | | NA |  |  |
| POS.ATT | -0.30 | | -0.37 | | -0.39 | | 0.72 | | -0.39 | | 1 | | | NA | | | NA | | NA | | NA | | NA | | | NA |  |  |
| POS.MED | -0.27 | | -0.34 | | -0.35 | | 0.65 | | -0.35 | | 0.61 | | | 1 | | | NA | | NA | | NA | | NA | | | NA |  |  |
| NEG.SLF | 0.31 | | 0.39 | | 0.40 | | -0.20 | | 0.40 | | -0.19 | | | -0.17 | | | 1 | | NA | | NA | | NA | | | NA |  |  |
| POS.SELF | 0.04 | | 0.05 | | 0.05 | | 0.20 | | 0.05 | | 0.19 | | | 0.17 | | | -0.23 | | 1 | | NA | | NA | | | NA |  |  |
| GP.POS | -0.26 | | -0.32 | | -0.34 | | 0.62 | | -0.34 | | 0.59 | | | 0.53 | | | -0.16 | | 0.16 | | 1 | | NA | | | NA |  |  |
| NHS.POS | -0.29 | | -0.36 | | -0.38 | | 0.70 | | -0.38 | | 0.66 | | | 0.59 | | | -0.18 | | 0.18 | | 0.57 | | 1 | | | NA |  |  |
| NHS.NEG | 0.46 | | 0.57 | | 0.59 | | -0.37 | | 0.59 | | -0.35 | | | -0.31 | | | 0.36 | | 0.05 | | -0.30 | | -0.34 | | | 1 |  |  |
| C19.CON | 0.47 | | 0.59 | | 0.61 | | -0.38 | | 0.61 | | -0.36 | | | -0.33 | | | 0.38 | | 0.05 | | -0.31 | | -0.35 | | | 0.55 |  |  |
| VAC.CON | 0.51 | | 0.64 | | 0.66 | | -0.42 | | 0.67 | | -0.39 | | | -0.35 | | | 0.41 | | 0.05 | | -0.34 | | -0.38 | | | 0.6 |  |  |
| OTH.DIS | -0.28 | | -0.35 | | -0.36 | | 0.26 | | -0.36 | | 0.25 | | | 0.23 | | | -0.50 | | 0.18 | | 0.22 | | 0.24 | | | -0.32 |  |  |
| OTH.NEG | -0.34 | | -0.42 | | -0.44 | | 0.24 | | -0.44 | | 0.22 | | | 0.20 | | | -0.54 | | -0.03 | | 0.19 | | 0.22 | | | -0.39 |  |  |
| ANGER | 0.36 | | 0.45 | | 0.47 | | -0.19 | | 0.47 | | -0.18 | | | -0.16 | | | 0.53 | | 0.00 | | -0.16 | | -0.18 | | | 0.42 |  |  |
| CHAOS | 0.45 | | 0.57 | | 0.58 | | -0.37 | | 0.59 | | -0.35 | | | -0.31 | | | 0.36 | | 0.05 | | -0.30 | | -0.34 | | | 0.53 |  |  |
| LIB | 0.12 | | 0.15 | | 0.16 | | 0.06 | | 0.16 | | 0.06 | | | 0.05 | | | 0.04 | | 0.08 | | 0.05 | | 0.06 | | | 0.14 |  |  |
| POP | 0.15 | | 0.19 | | 0.20 | | -0.05 | | 0.20 | | -0.05 | | | -0.04 | | | 0.08 | | -0.02 | | -0.04 | | -0.05 | | | 0.18 |  |  |
| REL.H | 0.29 | | 0.36 | | 0.37 | | -0.04 | | 0.37 | | -0.03 | | | -0.03 | | | 0.23 | | 0.27 | | -0.03 | | -0.03 | | | 0.33 |  |  |
| ILL.PUN | 0.40 | | 0.50 | | 0.51 | | -0.11 | | 0.52 | | -0.11 | | | -0.1 | | | 0.36 | | 0.23 | | -0.09 | | -0.10 | | | 0.46 |  |  |
| VAC.HES | 0.32 | | 0.40 | | 0.41 | | -0.42 | | 0.41 | | -0.40 | | | -0.36 | | | 0.06 | | -0.03 | | -0.34 | | -0.39 | | | 0.37 |  |  |
| MISTRUST | 0.63 | | 0.78 | | 0.81 | | -0.51 | | 0.81 | | -0.48 | | | -0.43 | | | 0.50 | | 0.07 | | -0.42 | | -0.47 | | | 0.73 |  |  |
| POS.HEALTHCARE | -0.36 | | -0.45 | | -0.47 | | 0.87 | | -0.47 | | 0.82 | | | 0.74 | | | -0.22 | | 0.23 | | 0.71 | | 0.80 | | | -0.42 |  |  |
|  | | | | | | | | | | | | | | | | | | | | | | | | | | | | |
| Cont. | | | | | | | | | | | | | | | | | | | | | | | | |  |  |  |  |
| Constructs | | C19.CON | | VAC.CON | | OTH.DIS | | OTH.NEG | | ANGER | | CHAOS | LIB | | POP | REL.H | | ILL.PUN | | VAC.HES | | MISTRUST | | POS.HEALTHCARE | | | |  |
| CVK | | NA | | NA | | NA | | NA | | NA | | NA | NA | | NA | NA | | NA | | NA | | NA | | NA | | | |  |
| GEN.K | | NA | | NA | | NA | | NA | | NA | | NA | NA | | NA | NA | | NA | | NA | | NA | | NA | | | |  |
| DIS.DOC | | NA | | NA | | NA | | NA | | NA | | NA | NA | | NA | NA | | NA | | NA | | NA | | NA | | | |  |
| RES.DOC | | NA | | NA | | NA | | NA | | NA | | NA | NA | | NA | NA | | NA | | NA | | NA | | NA | | | |  |
| NEG.VD | | NA | | NA | | NA | | NA | | NA | | NA | NA | | NA | NA | | NA | | NA | | NA | | NA | | | |  |
| POS.ATT | | NA | | NA | | NA | | NA | | NA | | NA | NA | | NA | NA | | NA | | NA | | NA | | NA | | | |  |
| POS.MED | | NA | | NA | | NA | | NA | | NA | | NA | NA | | NA | NA | | NA | | NA | | NA | | NA | | | |  |
| NEG.SLF | | NA | | NA | | NA | | NA | | NA | | NA | NA | | NA | NA | | NA | | NA | | NA | | NA | | | |  |
| POS.SELF | | NA | | NA | | NA | | NA | | NA | | NA | NA | | NA | NA | | NA | | NA | | NA | | NA | | | |  |
| GP.POS | | NA | | NA | | NA | | NA | | NA | | NA | NA | | NA | NA | | NA | | NA | | NA | | NA | | | |  |
| NHS.POS | | NA | | NA | | NA | | NA | | NA | | NA | NA | | NA | NA | | NA | | NA | | NA | | NA | | | |  |
| NHS.NEG | | NA | | NA | | NA | | NA | | NA | | NA | NA | | NA | NA | | NA | | NA | | NA | | NA | | | |  |
| C19.CON | | 1 | | NA | | NA | | NA | | NA | | NA | NA | | NA | NA | | NA | | NA | | NA | | NA | | | |  |
| VAC.CON | | 0.62 | | 1 | | NA | | NA | | NA | | NA | NA | | NA | NA | | NA | | NA | | NA | | NA | | | |  |
| OTH.DIS | | -0.33 | | -0.36 | | 1 | | NA | | NA | | NA | NA | | NA | NA | | NA | | NA | | NA | | NA | | | |  |
| OTH.NEG | | -0.41 | | -0.44 | | 0.8 | | 1 | | NA | | NA | NA | | NA | NA | | NA | | NA | | NA | | NA | | | |  |
| ANGER | | 0.44 | | 0.48 | | -0.45 | | -0.54 | | 1 | | NA | NA | | NA | NA | | NA | | NA | | NA | | NA | | | |  |
| CHAOS | | 0.55 | | 0.59 | | -0.32 | | -0.39 | | 0.42 | | 1 | NA | | NA | NA | | NA | | NA | | NA | | NA | | | |  |
| LIB | | 0.15 | | 0.16 | | -0.02 | | 0.01 | | 0.11 | | 0.14 | 1 | | NA | NA | | NA | | NA | | NA | | NA | | | |  |
| POP | | 0.19 | | 0.2 | | -0.09 | | 0.01 | | 0.12 | | 0.18 | 0.59 | | 1 | NA | | NA | | NA | | NA | | NA | | | |  |
| REL.H | | 0.34 | | 0.37 | | -0.18 | | -0.33 | | 0.32 | | 0.33 | 0.08 | | 0.03 | 1 | | NA | | NA | | NA | | NA | | | |  |
| ILL.PUN | | 0.48 | | 0.52 | | -0.30 | | -0.50 | | 0.49 | | 0.46 | 0.08 | | 0.03 | 0.77 | | 1 | | NA | | NA | | NA | | | |  |
| VAC.HES | | 0.39 | | 0.42 | | -0.10 | | -0.11 | | 0.11 | | 0.37 | 0.08 | | 0.13 | 0.08 | | 0.13 | | 1 | | NA | | NA | | | |  |
| MISTRUST | | 0.76 | | 0.82 | | -0.44 | | -0.54 | | 0.58 | | 0.72 | 0.19 | | 0.25 | 0.46 | | 0.64 | | 0.51 | | 1 | | NA | | | |  |
| POS.HEALTHCARE | | -0.44 | | -0.48 | | 0.30 | | 0.27 | | -0.22 | | -0.42 | 0.07 | | -0.06 | -0.04 | | -0.13 | | -0.48 | | -0.58 | | 1 | | | |  |
|  | | | | | | | | | | | | | | | | | | | | | | | | |  |  |  |  |

| Table S7. Variance inflation factor and tolerance levels | | |
| --- | --- | --- |
| Variables | Tolerance | VIF |
| I1 | 0.57 | 1.76 |
| I2 | 0.56 | 1.80 |
| Mistrust | 0.58 | 1.74 |
| POS.HC | 0.57 | 1.77 |

| Table S8. Parameter estimates of the final SEM model. | | | | |
| --- | --- | --- | --- | --- |
| Variables | *B* | SE *B* | 𝛽 | P |
| I1 | 0.05 | 0.01 | 0.05 | <0.001 |
| I2 | 0.02 | 0.01 | 0.02 | 0.044 |
| Mistrust | 0.42 | 0.04 | 0.28 | <0.001 |
| POS.HC | -0.59 | 0.04 | -0.34 | <0.001 |
|  | | | | |

**Exploratory and Confirmatory Factor Analysis**

We used the psych package (Revelle, 2017) to conduct exploratory factor analysis (EFA). The data was randomly separated into two equal size for measures that employed both EFA and CFA. The Kaiser-Meyer-Olkin (KMO) index is a measure of sampling adequacy with values ranging between 0 and 1 (Kaiser, 1970). Values closer to 1 suggest that the data is likely to recover latent factor(s) based on the correlation matrix (Williams, Onsman & Brown, 2010). Thus, the KMO value is reported as necessary when an EFA is employed. A parallel analysis (*n* iterations = 1,000) was subsequently conducted based on the principle axis factor analysis to determine latent dimensionality. Minimum residual solution was used to analyse the factor structure (Tellegen & Waller, 2008). The approach achieved a factor solution by minimizing the residuals of the correlation matrix and hence, obtaining communalities for the items (Harman & Jones, 1966). The factors for all EFA models were extracted using oblimin rotation.

The lavaan package was used to conduct confirmatory factor analysis (Rosseel, 2012). We used the same estimation method and goodness-of-fit criteria as described in the analysis section to assess item parameter estimates and the model fit.

***Oxford COVID-19 Vaccine Hesitancy Scale***

Exploratory factor analysis

Items B4, B7 and B13 were removed prior to EFA because it failed qualitative check. The overall KMO was 0.98. At an item level, the lowest KMO was 0.97 and the highest KMO was 0.99. Parallel analysis indicated a dominant single factor (Figure S2). EFA was conducted on a sample of 2557 participants. A one-factor solution EFA showed that it accounted for 84% of the variance.

Figure S2. Parallel Analysis


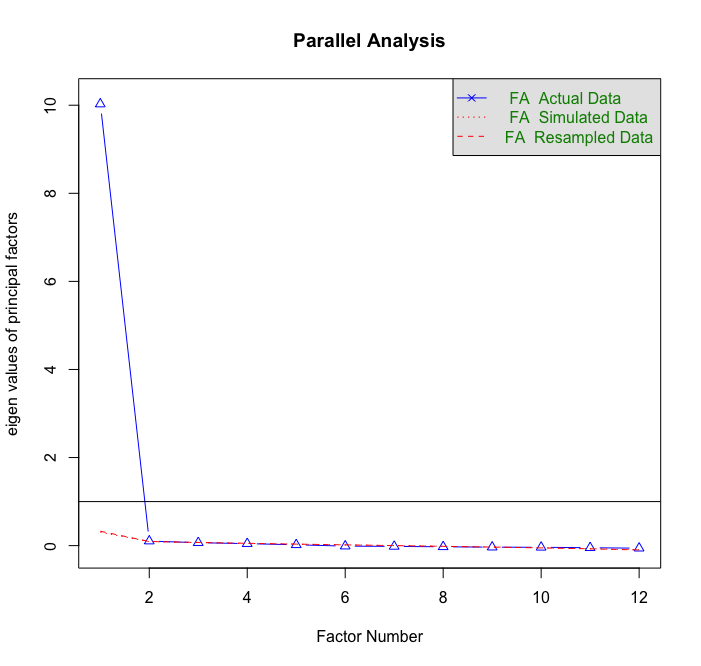


| Table A. Standardized loadings based on exploratory factor loadings | |
| --- | --- |
| Item | Factor loading |
| Item 1 (B1) | 0.92 |
| Item 2 (B2) | 0.91 |
| Item 3 (B3) | 0.91 |
| Item 5 (B5) | 0.92 |
| Item 6 (B6) | 0.87 |
| Item 8 (B8) | 0.92 |
| Item 9 (B9) | 0.92 |
| Item 10 (B10) | 0.95 |
| Item 11 (B11) | 0.92 |
| Item 12 (B12) | 0.89 |
| Item 14 (B14) | 0.92 |
| Item 15 (B15) | 0.91 |
| *Note*. Standardized item loadings for a single factor. | |

Confirmatory factor analysis

Initial review of the measurement model indicated that B15 had high modification indexes with several other items and was therefore removed from the analysis. Item B10 was also removed because the factor loading was substantially higher relative to the other items. Items B8, B9 and B14 were removed because we identified several item content overlap with the other remaining items. This resulted in a 7-item measure. The final CFA model indicated an excellent model fit (FIML, χ2(14, N = 2548) = 93.370, p <0.001, RMSEA = 0.047; SRMR = 0.01; CFI = 0.993; TLI = 0.989). The Cronbach’s alpha was 0.97.

| Table B. Standardized loadings based on confirmatory factor loadings | | | |
| --- | --- | --- | --- |
| Item | Factor loading | Mean | SD |
| Item 1 (B1) | 0.92 | 1.94 | 1.19 |
| Item 2 (B2) | 0.92 | 2.16 | 1.15 |
| Item 3 (B3) | 0.91 | 2.22 | 1.19 |
| Item 5 (B5) | 0.92 | 1.98 | 1.28 |
| Item 6 (B6) | 0.88 | 2.04 | 1.13 |
| Item 11 (B11) | 0.92 | 2.12 | 1.13 |
| Item 12 (B12) | 0.89 | 1.88 | 1.07 |
| *Note*. Standardized item loadings for a single factor. | | | |

***Oxford COVID-19 Vaccine Confidence & Complacency Scale***

Exploratory factor analysis

The overall KMO was 0.96. At an item level, the lowest KMO was 0.92 and the highest KMO was 0.97. Parallel analysis indicated a dominant single factor with 3 additional factors above the eigenvalues derived from random or simulated data (Figure S3). Thus, four-factor EFA was conducted on a sample of 2557 participants and a four-factor solution. The result of the EFA showed that it accounted for 65% of the variance.

Figure S3. Parallel Analysis


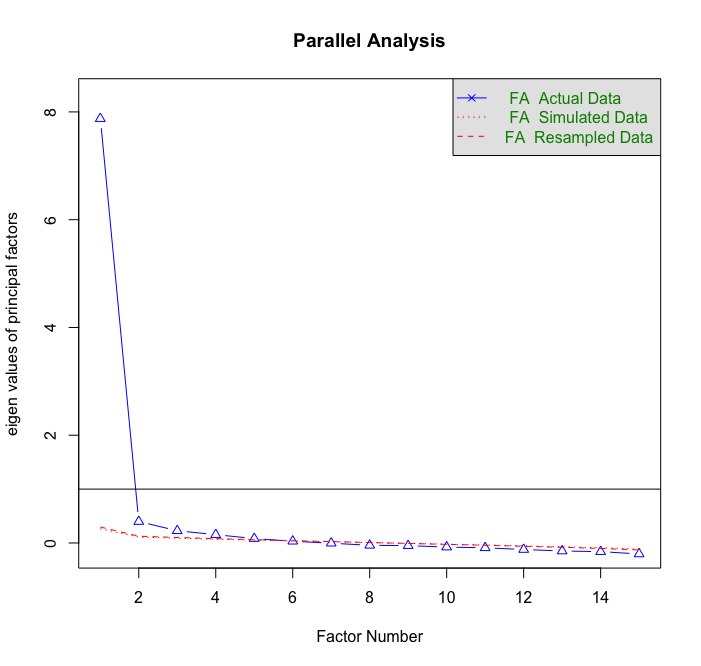


| Table C. Standardized loadings based on exploratory factor loadings | | | | |
| --- | --- | --- | --- | --- |
| Item | Factor 1 | Factor 2 | Factor 3 | Factor 4 |
| B16 | - | 0.43 | - | - |
| B17 | - | 0.82 | - | - |
| B18 | - | 0.91 | - | - |
| B20 | 0.78 | - | - | - |
| B21 | 0.76 | - | - | - |
| B22 | - | - | 0.76 | - |
| B23 | - | - | 0.92 | - |
| B24 | 0.77 | - | - | - |
| B26 | 0.36 | - | - | - |
| B27 | - | - | - | 0.41 |
| B28 | 0.68 | - | - | - |
| B32 | 0.74 | - | - | - |
| B33 | 0.63 | - | - | - |
| B34 | - | - | 0.32 | 0.38 |
| Note. Standardized item loadings for a four-factor solution. | | | | |

Confirmatory factor analysis

Upon reviewing the items loaded onto the various factors in the EFA, we found that the content of item B26 was similar to that of B27 and B34 and item B33 was similar to that of B22 and B23. Hence, they were grouped together as factors. . All the other items remained in their respective factors as indicated by EFA. A four-factor CFA was conducted and the model indicated an excellent fit (FIML, χ2(71, N = 2541) = 524.205, p <0.001, RMSEA = 0.050; SRMR = 0.03; CFI = 0.973; TLI = 0.965). The Cronbach’s alpha for IMPT was 0.90; SDV was 0.88; WRK was 0.77; SEF was 0.78.

| Table D. Standardized loadings based on confirmatory factor loadings | | | | |
| --- | --- | --- | --- | --- |
| Items | Factors | Factor loadings | Mean | SD |
| B20 | IMPT | 0.86 | 1.85 | 1.01 |
| B21 |  | 0.86 | 1.72 | 0.95 |
| B24 |  | 0.65 | 2.05 | 1.01 |
| B28 |  | 0.82 | 2.26 | 0.97 |
| B32 |  | 0.82 | 2.22 | 0.94 |
| B22 | SDV | 0.89 | 2.36 | 1.14 |
| B23 |  | 0.89 | 2.47 | 1.12 |
| B33 |  | 0.74 | 2.43 | 0.92 |
| B16 | WRK | 0.44 | 2.95 | 1.06 |
| B17 |  | 0.87 | 2.36 | 0.96 |
| B18 |  | 0.90 | 2.4 | 0.92 |
| B26 | SEF | 0.66 | 1.97 | 0.97 |
| B27 |  | 0.78 | 2.47 | 0.95 |
| B34 |  | 0.74 | 2.23 | 1.28 |
| Note. Standardized item loadings for a four-factor solution. | | | | |

**Vaccine Hesitancy Scale (VHS) (Shapiro et al, 2018)**

An EFA and CFA was previously conducted by Shapiro et al. (2018) that identified two factors, with seven items measuring ‘lack of confidence’ and two items measuring ‘risk’. Thus, we only conducted a CFA to evaluate factor loadings and model goodness-of-fit in this study. However, the initial two-factor CFA model showed negative variance estimates (item 9), which is also known as Heywood cases, indicating potential structural misspecification (Kolenikov & Bollen, 2012). Thus, we reverted to running a one-factor CFA model, which showed that item 5 and 9 had poor loadings. These items were thus, considered unreliable and subsequently removed. A one-factor CFA model was refitted to evaluate the remaining items and the result showed adequate model fit (FIML, χ2(14, N = 5114) = 464.403, p <0.001, RMSEA = 0.079; SRMR = 0.034; CFI = 0.959; TLI = 0.938). A one-factor CFA model of vaccine hesitancy is also supported by a previous study conducted by Domek et al. (2018). The Cronbach’s alpha was 0.94.

| **Children Vaccine Hesitancy scale** | | |  |
| --- | --- | --- | --- |
| Table E. Standardized loadings based on exploratory factor loadings | | | |
| Item | Factor loading | Mean | SD |
| Item 1 (C1_1) | 0.84 | 4.21 | 1.08 |
| Item 2 (C1_2) | 0.88 | 4.18 | 1.01 |
| Item 3 (C1_3) | 0.88 | 4.18 | 1.02 |
| Item 4 (C1_4) | 0.87 | 4.12 | 1.03 |
| Item 6 (C1_6) | 0.67 | 3.73 | 0.97 |
| Item 7 (C1_7) | 0.84 | 4.21 | 0.96 |
| Item 8 (C1_8) | 0.78 | 4.02 | 1.00 |
| *Note*. Standardized item loadings for a single factor. | | | |

Construct and Face Validity between Oxford vaccine hesitancy and Children Vaccine Hesitancy Scale.

The Oxford Vaccine Hesitancy measure exhibited a positive Pearson correlation with the one-factor VHS measure (*r* = 0.47, p < 0.001). Therefore, the common variance shared between both tests was 22%. This is not enough to suggest that the measures are assessing the same construct, but they are nonetheless related constructs. This is not surprising, given that the VHS consist of questions asking about general vaccinations in childhood, whereas the Oxford vaccine hesitancy scale consists of questions asking about Covid-19 vaccine.

Furthermore, face validity of the measure was evidenced through the development process involving clinical psychologists. Due to the unprecedented nature of this construct, there were no existing measure that was capable of assessing the same construct for the assessment of convergent validity.

**Vaccination Knowledge Scale (Zingg & Siergrist, 2012)**

Exploratory factor analysis

The overall KMO was 0.87. At an item level, the lowest KMO was 0.83 and the highest KMO was 0.90. Parallel analysis indicated three factors above the eigenvalues derived from random or simulated data (Figure S4). We conducted a two- and three-factor EFA on a sample of 2557 participants and found that a two-factor EFA solution provided clearer interpretable factors based on the theoretical model. Furthermore, a two-factor EFA solution accounted for 43% of the variance, whilst a three-factor EFA solution merely increased the amount of variance explained by 4%. Thus, a two-factor EFA model was retained as the final solution. Inspection of the factor loadings showed that item 6 had cross loadings and was removed from the analysis. The table below shows the factor loadings of the final two-factor EFA model.

Figure S4. Parallel Analysis


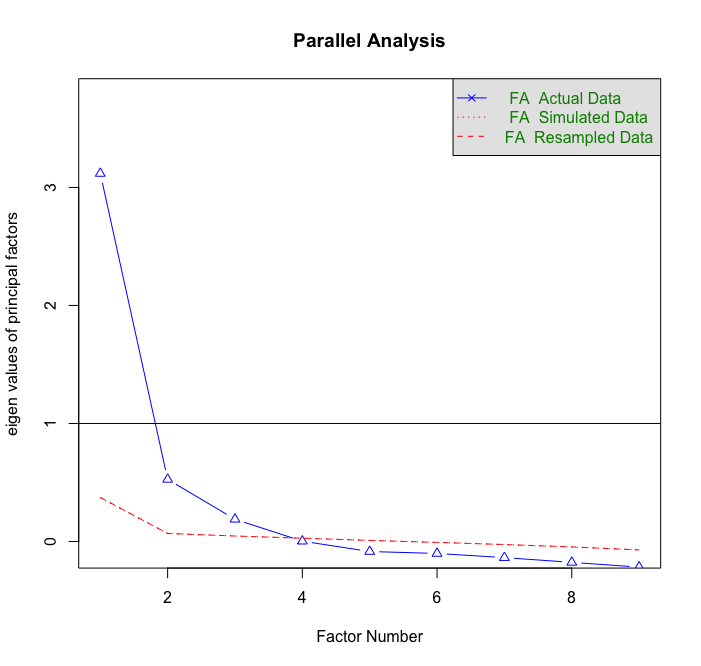


| Table F. Standardized loadings based on exploratory factor loadings | | |
| --- | --- | --- |
| Item | Factor 1 | Factor 2 |
| Item 1 (D1_1) | - | 0.44 |
| Item 2 (D1_2) | - | 0.76 |
| Item 3 (D1_3) | - | 0.70 |
| Item 4 (D1_4) | 0.44 | - |
| Item 5 (D1_5) | 0.61 | - |
| Item 7 (D1_7) | 0.71 | - |
| Item 8 (D1_8) | - | 0.44 |
| Item 9 (D1_9) | 0.76 | - |
| Note. Standardized item loadings for a two-factor solution. | | |

Confirmatory factor analysis

We conducted a two-factor CFA but the modification indices (MIs) were high for item 2 and item 3. We decided to correlate the residuals since we considered this a reasonable minimum modification as the within factor residuals covariance is likely a representative of a non-random error derived from subdomains of the same factor (Gerbing & Anderson, 1984). Furthermore, it may reduce inflated factor loadings due to larger covariance between paired items, when some of which is actually due to unique factor covariance (Hayes & Usami, 2020). Moreover, when the focus is on accurately estimating the structural model rather than the measurement model, simulation results from Hayes & Usami (2020) suggested that within-factor correlated uniqueness models almost always attenuate rather than magnify the biased structural parameters. The final CFA indicated a reasonable fit (FIML, χ2(18, N = 2557) = 211.920, p <0.001, RMSEA = 0.065; SRMR = 0.039; CFI = 0.958; TLI = 0.935). The Cronbach’s alpha for both general and childhood knowledge was 0.77.

| Table G. Standardized loadings based on confirmatory factor loadings | | | |
| --- | --- | --- | --- |
| Items | Factors | Factor loadings |  |
| Item 1 (D1_1) | General Knowledge | 0.68 |  |
| Item 2 (D1_2) |  | 0.61 |  |
| Item 3 (D1_3) |  | 0.65 |  |
| Item 8 (D1_8) |  | 0.65 |  |
| Item 4 (D1_4) | Childhood Knowledge | 0.64 |  |
| Item 5 (D1_5) |  | 0.63 |  |
| Item 7 (D1_7) |  | 0.73 |  |
| Item 9 (D1_9) |  | 0.69 |  |
| Note. Standardized item loadings for a two-factor solution. | | | |

**Oxford Trust in Doctors and Developers Questionnaire**

Items 10 and 12 were removed from analysis because the content was not sufficiently clear. Thus, an EFA and CFA was conducted on the 14-item measure.

Exploratory factor analysis

The overall KMO was 0.93. At an item level, the lowest KMO was 0.87 and the highest KMO was 0.97. Parallel analysis indicated three factors above and one factor marginally above the eigenvalues derived from random or simulated data (Figure S5). Therefore, we conducted a three- and four-factor EFA on a sample of 2557 participants and found that a three-factor EFA solution provided clearer interpretable factors based on the theoretical model. Furthermore, a three-factor EFA solution accounted for 60% of the variance, whilst a four-factor EFA solution merely increased the amount of variance explained by 2%. Thus, a three-factor EFA model was retained as the final solution. The table below shows the factor loadings of the final three-factor EFA model.

Figure S5. Parallel Analysis


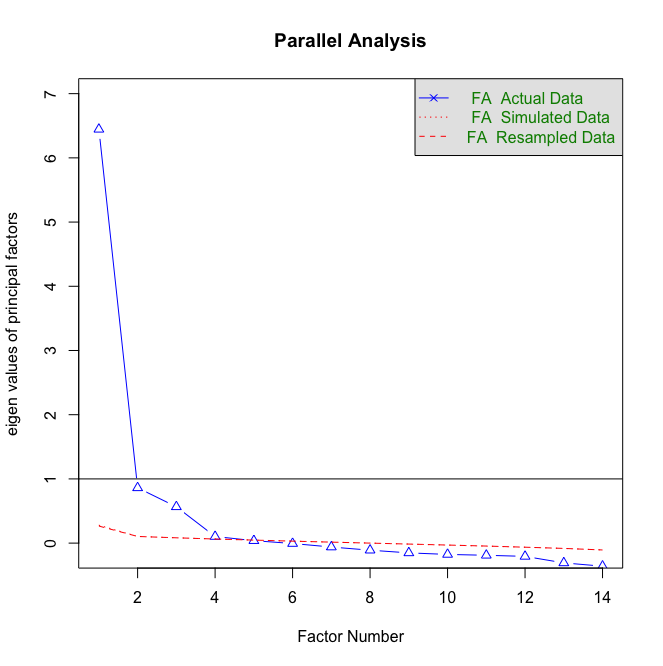


| Table H. Standardized loadings based on exploratory factor loadings | | | |
| --- | --- | --- | --- |
| Items | Factor 1 | Factor 2 | Factor 3 |
| Item 1 (G1_1) | - | - | 0.82 |
| Item 2 (G1_2) | 0.65 | - | - |
| Item 3 (G1_3) | - | - | 0.79 |
| Item 4 (G1_4) | 0.86 | - | - |
| Item 5 (G1_5) | 0.92 | - | - |
| Item 6 (G1_6) | - | - | 0.69 |
| Item 7 (G1_7) | 0.54 | - | - |
| Item 8 (G1_8) | 0.51 | - | - |
| Item 9 (G1_9) | 0.57 | - | - |
| Item 11 (G1_11) | 0.55 | - | - |
| Item 13 (G1_13) | - | 0.84 | - |
| Item 14 (G1_14) | - | 0.79 | - |
| Item 15 (G1_15) | - | 0.81 | - |
| Item 16 (G1_16) | - | 0.87 | - |
| Note. Standardized item loadings for a three-factor solution. | | | |

Confirmatory factor analysis

We conducted an initial three-factor CFA and found that the MIs for item 4 and item 5 were very high. Reviewing these items suggested that they were indeed very similar to each other; “They look down on me – Item 4” and “They have little respect for me – Item 5”. Thus, we decided to remove item 4 and the final model showed improvement in the goodness of fit of the model (FIML, χ2(62, N = 2557) = 393.540, p <0.001, RMSEA = 0.046; SRMR = 0.025; CFI = 0.972; TLI = 0.965). The Cronbach’s alpha for disrespect doctor is 0.87; respect doctor is 0.81; negative vaccine developers was 0.9.

| Table I. Standardized loadings based on confirmatory factor loadings | | | | |
| --- | --- | --- | --- | --- |
| Items | Factors | Factor loadings | Mean | SD |
| Item 2 (G1_2) | Disrespect Doc | 0.69 | 2.45 | 1.27 |
| Item 5 (G1_5) |  | 0.78 | 2.32 | 1.25 |
| Item 7 (G1_7) |  | 0.58 | 3.26 | 1.28 |
| Item 8 (G1_8) |  | 0.68 | 2.90 | 1.21 |
| Item 9 (G1_9) |  | 0.76 | 2.44 | 1.26 |
| Item 11 (G1_11) |  | 0.83 | 2.12 | 1.22 |
| Item 1 (G1_1) | Respect Doc | 0.77 | 4.16 | 0.92 |
| Item 3 (G1_3) |  | 0.77 | 4.02 | 1.02 |
| Item 6 (G1_6) |  | 0.77 | 4.30 | 0.88 |
| Item 13 (G1_13) | Negative Vaccine Developers | 0.84 | 2.54 | 1.25 |
| Item 14 (G1_14) |  | 0.80 | 2.73 | 1.3 |
| Item 15 (G1_15) |  | 0.86 | 2.38 | 1.2 |
| Item 16 (G1_16) |  | 0.84 | 2.46 | 1.28 |
| Note. Standardized item loadings for a three-factor solution. | | | | |

**Attitudes to Doctors and Medicine Questionnaire (Marteau, 1990). [only positive factors used in SEM]**

Confirmatory factor analysis

According to Marteau (1990), the scale measuring attitudes towards medicine and doctors comprises of four factors: ‘positive attitude towards doctors’, ‘positive attitude towards medicine’, ‘negative attitude towards doctors’ and ‘negative attitude towards medicine’. The scale was analysed using principle component analysis. Hence, it was not necessary to conduct an EFA. Nonetheless, we conducted a CFA to inspect the loadings and model fit to the data.

An initial three-factor CFA showed that item 9 and item 16 had loadings of less than 0.4 and was therefore, were removed from analysis. Further inspection of the modification indices showed that item 2 and item 11 had high MIs with several items and was thus, removed from subsequent analysis. The resulting model indicated a good fit (FIML, χ2(71, N = 5114) = 897.943, p <0.001, RMSEA = 0.048; SRMR = 0.051; CFI = 0.96; TLI = 0.95). The Cronbach’s alpha for positive attitude towards doctors was 0.85; positive attitude towards medicine was 0.69; negative attitude towards doctors was 0.82; negative attitude towards medicine was 0.80.

| Table J. Standardized loadings based on confirmatory factor loadings | | | | |
| --- | --- | --- | --- | --- |
| Items | Factors | Factor loadings | Mean | SD |
| Item 2 (H1_2) | POS.DOC | 0.76 | 3.78 | 1.21 |
| Item 3 (H1_3) |  | 0.84 | 3.99 | 1.17 |
| Item 4 (H1_4) |  | 0.84 | 4.04 | 1.08 |
| Item 11 (H1_11) | POS.MED | 0.43 | 4.50 | 1.09 |
| Item 13 (H1_13) |  | 0.71 | 4.17 | 1.08 |
| Item 14 (H1_14) |  | 0.63 | 3.73 | 1.20 |
| Item 6 (H1_6) | NEG.DOC | 0.78 | 2.84 | 1.29 |
| Item 7 (H1_7) |  | 0.72 | 3.20 | 1.23 |
| Item 8 (H1_8) |  | 0.68 | 3.15 | 1.34 |
| Item 10 (H1_10) |  | 0.73 | 2.50 | 1.34 |
| Item 15 (H1_15) | NEG.MED | 0.54 | 3.62 | 1.25 |
| Item 17 (H1_17) |  | 0.80 | 2.85 | 1.26 |
| Item 18 (H1_18) |  | 0.84 | 2.84 | 1.29 |
| Item 19 (H1_19) |  | 0.66 | 3.29 | 1.24 |
| Note. Standardized item loadings for a four-factor solution. POS.DOC = Positive attitude towards doctors. POS.MED = Positive attitude towards medicine. NEG.DOC = Negative attitude towards doctors. NEG.MED = Negative attitude towards medicine. | | | | |

***Brief Core Schema Scales – Self scales (BCSS) (Fowler et al, 2006)***

Confirmatory factor analysis

According to Fowler et. al (2006), the Brief Core Schema Scales (BCSS) consisted of 4 factors: ‘positive self’, ‘positive other’, ‘negative self’ and ‘negative other. However, we only used ‘positive self’ and ‘negative self’ in this study. We decided that an EFA was not necessary since a principle component analysis was conducted previously, which identified a four-factor model. Nonetheless, we conducted a CFA to inspect the factor loadings and model fit to the data.

An initial 2-factor CFA identified high MIs for 3 items (item 1, item 8 and item 11) and was subsequently removed from analysis. The resulting model indicated a good fit (FIML, χ2(26, N = 5114) = 563.428, p <0.001, RMSEA = 0.064; SRMR = 0.042; CFI = 0.967; TLI = 0.955). The Cronbach’s alpha for negative self was 0.9 and 0.87 for positive self.

| Table K. Standardized loadings based on confirmatory factor loadings | | | | |
| --- | --- | --- | --- | --- |
| Items | Factors | Factor loadings | Mean | SD |
| Item 2 (J1_1) | Negative Self | 0.88 | 1.73 | 1.14 |
| Item 3 (J1_3) |  | 0.88 | 1.76 | 1.13 |
| Item 4 (J1_4) |  | 0.72 | 2.05 | 1.20 |
| Item 5 (J1_5) |  | 0.78 | 1.56 | 1.03 |
| Item 6 (J1_6) |  | 0.88 | 1.79 | 1.19 |
| Item 7 (J1_7) | Positive Self | 0.73 | 2.99 | 1.14 |
| Item 9 (J1_9) |  | 0.83 | 2.90 | 1.22 |
| Item 10 (J1_10) |  | 0.84 | 2.73 | 1.22 |
| Item 12 (J1_12) |  | 0.77 | 3.10 | 1.16 |
| Note. Standardized item loadings for a two-factor solution. | | | | |

***General Practice Assessment Questionnaire-R2 (GPAQ-R2) (Rowland et al, 2013***

Exploratory factor analysis

The overall KMO was 0.95. At an item level, the lowest KMO was 0.95 and the highest KMO was 0.96. Parallel analysis indicated two factors above the eigenvalues derived from random or simulated data (Figure S6). Therefore, we conducted a two- and one-factor EFA on a sample of 2557 participants. We found that all items loaded onto a one-factor EFA solution and accounted for 75% of the variance. Thus, a one-factor EFA model was retained as the final solution. The table below shows the factor loadings of the one-factor EFA model.

Figure S6. Parallel Analysis


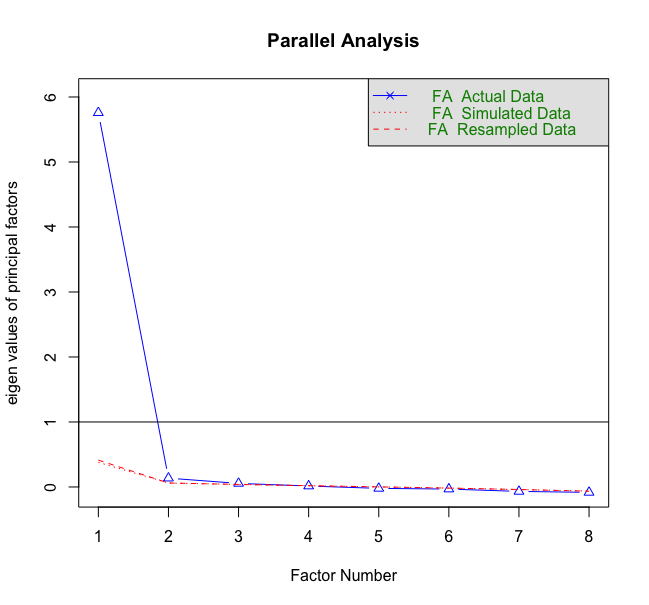


| Experiences with healthcare | |
| --- | --- |
| Table L. Standardized loadings based on exploratory factor loadings | |
| Item | Factor loading |
| Item 1 (K1_1) | 0.83 |
| Item 2 (K1_2) | 0.81 |
| Item 3 (K1_3) | 0.88 |
| Item 4 (K1_4) | 0.81 |
| Item 5 (K1_5) | 0.87 |
| Item 6 (K1_6) | 0.87 |
| Item 7 (K1_7) | 0.86 |
| Item 8 (K1_8) | 0.85 |
| *Note*. Standardized item loadings for a single factor. | |

Confirmatory factor analysis

We conducted an initial one-factor CFA and found that item 1, item 3 and item 5 had high MIs with several other items. Thus, we decided to remove these 3 items and the final model indicated an excellent model fit (FIML, χ2(5, N = 2498) = 13.546, p < 0.05, RMSEA = 0.026; SRMR = 0.007; CFI = 0.998; TLI = 0.996). The Cronbach’s alpha was 0.93.

| Table M. Standardized loadings based on confirmatory factor loadings | | | |
| --- | --- | --- | --- |
| Item | Factor loading | Mean | SD |
| Item 2 (K1_2) | 0.79 | 2.09 | 1.03 |
| Item 4 (K1_4) | 0.80 | 2.42 | 1.12 |
| Item 6 (K1_6) | 0.89 | 2.22 | 1.04 |
| Item 7 (K1_7) | 0.88 | 2.28 | 1.03 |
| Item 8 (K1_8) | 0.88 | 2.22 | 1.05 |
| *Note*. Standardized item loadings for a single factor. | | | |

***NHS experience questionnaire***

Exploratory factor analysis

The overall KMO was 0.81. At an item level, the lowest KMO was 0.71 and the highest KMO was 0.87. Parallel analysis indicated three factors above the eigenvalues derived from random or simulated data (Figure S7). We conducted a two- and three-factor EFA on a sample of 2557 participants and found that a two-factor EFA solution provided clearer interpretable factors based on the theoretical model. The two-factor EFA solution accounted for 49% of the variance. Thus, a two-factor EFA model was retained as the final solution. The table below shows the factor loadings of the final two-factor EFA model.

Figure S7. Parallel Analysis


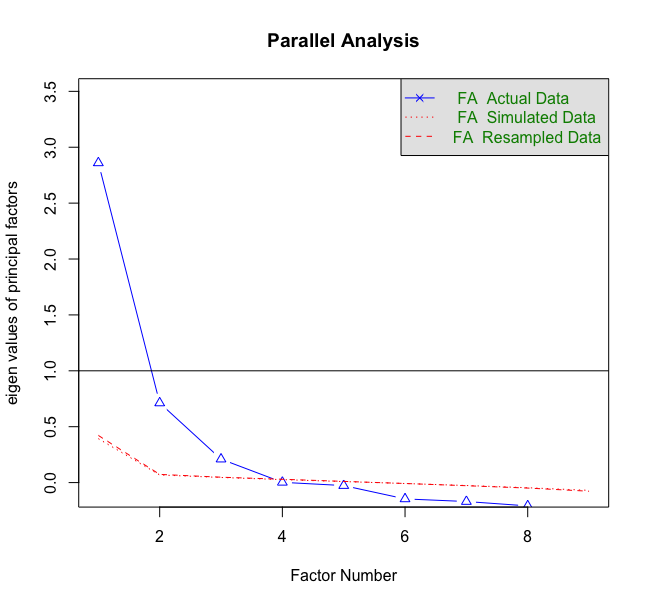


| Table N. Standardized loadings based on exploratory factor loadings | | |
| --- | --- | --- |
| Item | Factor 1 | Factor 2 |
| Item 1 (K3_1) | - | 0.78 |
| Item 2 (K3_2) | 0.56 | - |
| Item 3 (K3_3) | - | 0.73 |
| Item 4 (K3_4) | 0.75 | - |
| Item 5 (K3_5) | 0.73 | - |
| Item 6 (K3x2_1) | - | 0.52 |
| Item 7 (K3x2_2) | 0.63 | - |
| Item 8 (K3x2_3) | 0.69 | - |
| Note. Standardized item loadings for a two-factor solution. | | |

Confirmatory factor analysis

We conducted a two-factor CFA, with items reflective of the constructs ‘positive NHS experience’ and ‘negative NHS experience’. Item 7 was found to have high MIs with several items and was removed from the analysis. Item 1 and item 3 also had high MIs but were theoretically important to remain within the construct as a measure of positive healthcare experience and to ensure that the model is identifiable with at least 3 items per factor (Marsh, Hau, Balla, & Grayson, 1998). Therefore, we decided to correlate the residuals of those two items. The final model indicated an acceptable model fit (FIML, χ2(12, N = 2557) = 162.765, p <0.001, RMSEA = 0.070; SRMR = 0.040; CFI = 0.952; TLI = 0.916). The Cronbach’s alpha for positive healthcare experience was 0.74 and 0.78 for negative healthcare experience.

| Table O. Standardized loadings based on confirmatory factor loadings | | | | |
| --- | --- | --- | --- | --- |
| Items | Factors | Factor loadings | Mean | SD |
| Item 1 (K3_1) | Positive Healthcare Experience | 0.61 | 2.60 | 0.64 |
| Item 3 (K3_3) |  | 0.46 | 2.39 | 0.75 |
| Item 6 (K3x2_1) |  | 0.83 | 2.51 | 0.62 |
| Item 2 (K3_2) | Negative Healthcare Experience | 0.57 | 1.68 | 0.78 |
| Item 4 (K3_4) |  | 0.81 | 1.41 | 0.72 |
| Item 5 (K3_5) |  | 0.76 | 1.51 | 0.77 |
| Item 8 (K3x2_3) |  | 0.64 | 1.37 | 0.64 |
| Note. Standardized item loadings for a two-factor solution. | | | | |

**OCEANS Coronavirus Conspiracy Scale (Freeman et al, 2020)**

Confirmatory factor analysis

According to our analysis of data from the first OCEAN survey, the measure consisted of 1 factor based on an EFA and CFA analysis. Thus, conducting an EFA was not required. We did however, conducted a CFA to inspect the loadings and model fit to the data.

An initial one-factor CFA model indicated that item 2 had high MIs with several items and was removed from analysis. The final one-factor CFA model indicated an excellent fit (FIML, χ2(9, N = 4983) = 68.926, p <0.001, RMSEA = 0.037; SRMR = 0.01; CFI = 0.993; TLI = 0.988). The Cronbach’s alpha was 0.94.

| Table P. Standardized loadings based on confirmatory factor loadings | | | |
| --- | --- | --- | --- |
| Item | Factor loading | Mean | SD |
| Item 1 (L1_1) | 0.66 | 1.53 | 1.08 |
| Item 3 (L1_3) | 0.85 | 2.01 | 1.37 |
| Item 4 (L1_4) | 0.91 | 1.92 | 1.33 |
| Item 5 (L1_5) | 0.92 | 1.94 | 1.36 |
| Item 6 (L1_6) | 0.87 | 2.04 | 1.38 |
| Item 7 (L1_7) | 0.91 | 1.90 | 1.34 |
| *Note*. Standardized item loadings for a single factor. | | | |

**Vaccine Conspiracy Beliefs Scale (Shapiro et al, 2016)**

Confirmatory factor analysis

According to Shapiro, Holding, Perez, Amsel & Rosberger (2016), the 7-item measure consisted of 1 factor based on a principle component analysis. Thus, we only conducted a CFA to inspect the factor loadings and model fit to the data.

A one-factor CFA model indicated a good fit (FIML, χ2(14, N = 5114) = 304.607, p <0.001, RMSEA = 0.064; SRMR = 0.017; CFI = 0.981; TLI = 0.971). The Cronbach’s alpha was 0.96.

| Table Q. Standardized loadings based on confirmatory factor loadings | | | |
| --- | --- | --- | --- |
| Item | Factor loading | Mean | SD |
| Item 1 (N1_1) | 0.83 | 3.11 | 1.65 |
| Item 2 (N1_2) | 0.85 | 2.70 | 1.65 |
| Item 3 (N1_3) | 0.87 | 3.24 | 1.71 |
| Item 4 (N1_4) | 0.92 | 3.18 | 1.71 |
| Item 5 (N1_5) | 0.92 | 3.08 | 1.71 |
| Item 6 (N1_6) | 0.93 | 3.17 | 1.75 |
| Item 7 (N1_7) | 0.86 | 2.98 | 1.78 |
| *Note*. Standardized item loadings for a single factor. | | | |

**Everyday discrimination scale (Williams et al, 1997)**

Exploratory factor analysis

The overall KMO was 0.91. At an item level, the lowest KMO was 0.87 and the highest KMO was 0.95. Parallel analysis indicated three factors above the eigenvalues derived from random or simulated data (Figure S8). Thus, we conducted a two- and three-factor EFA on a sample of 2557 participants and found that a two-factor EFA solution provided clearer interpretable factors based on the theoretical model. The two-factor EFA solution accounted for 69% of the variance. Thus, a two-factor EFA model was retained as the final solution. The table below shows the factor loadings of the final two-factor EFA model.

Figure S8. Parallel Analysis


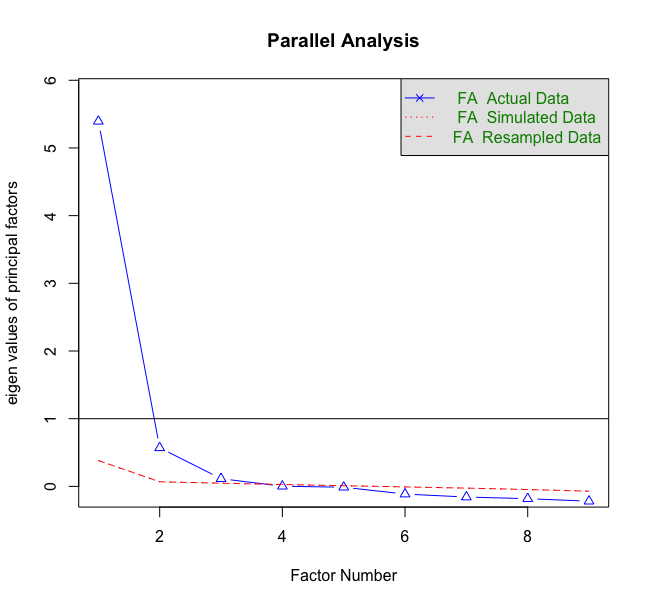


| Table R. Standardized loadings based on exploratory factor loadings | | |
| --- | --- | --- |
| Item | Factor 1 | Factor 2 |
| Item 1 (O1_1) | 0.91 | - |
| Item 2 (O1_2) | 0.99 | - |
| Item 3 (O1_3) | 0.52 | - |
| Item 4 (O1_4) | 0.68 | - |
| Item 5 (O1x2_1) | - | 0.78 |
| Item 6 (O1x2_2) | - | 0.87 |
| Item 7 (O1x2_3) | 0.54 | - |
| Item 8 (O1x2_4) | - | 0.74 |
| Item 9 (O1x2_5) | - | 0.82 |
| Note. Standardized item loadings for a two-factor solution. | | |

Confirmatory factor analysis

We conducted a two-factor CFA, with items reflective of the constructs ‘others disrespect’ and ‘others react’. Item 1 and item 8 were found to have high MIs with several items and was removed from the analysis. Item 3 also had high MI with item 7 but the items were theoretically important to remain within the construct. Therefore, we decided to correlate the residuals of those two items. The final model indicated a good model fit (FIML, χ2(12, N = 2557) = 126.614, p <0.001, RMSEA = 0.061; SRMR = 0.03; CFI = 0.980; TLI = 0.965). The Cronbach’s alpha for others disrespect was 0.89 and 0.86 for others react.

| Table S. Standardized loadings based on confirmatory factor loadings | | | | |
| --- | --- | --- | --- | --- |
| Items | Factors | Factor loadings | Mean | SD |
| Item 2 (O1_2) | Others Disrespect | 0.83 | 4.33 | 1.52 |
| Item 3 (O1_3) |  | 0.83 | 4.72 | 1.41 |
| Item 4 (O1_4) |  | 0.85 | 4.42 | 1.58 |
| Item 7 (O1x2_3) |  | 0.79 | 4.13 | 1.53 |
| Item 5 (O1x2_1) | Others React | 0.79 | 5.04 | 1.43 |
| Item 6 (O1x2_2) |  | 0.88 | 5.02 | 1.39 |
| Item 9 (O1x2_5) |  | 0.79 | 5.01 | 1.37 |
| Note. Standardized item loadings for a two-factor solution. | | | | |

**Dimensions of Anger Reactions-5 (DAR-5)**

Confirmatory factor analysis

According to Forbes et. al (2014), a CFA supported a single factor model of the 5-item measure. Thus, we only conducted a CFA to inspect the factor loadings and model fit to the data. Unlike the study conducted by Forbest et. al (2014) however, we found high MI for item 4 and item 5. To ensure the integrity of the factorial structure, we decided to correlate the residuals for these two items. The final CFA model resulted in a good model fit (FIML, χ2(4, N = 5114) = 93.859, p <0.001, RMSEA = 0.066; SRMR = 0.016; CFI = 0.989; TLI = 0.973). The Cronbach’s alpha was 0.91.

| Table T. Standardized loadings based on confirmatory factor loadings | | | |
| --- | --- | --- | --- |
| Item | Factor loading | Mean | SD |
| Item 1 (P1_1) | 0.75 | 2.25 | 1.12 |
| Item 2 (P1_2) | 0.89 | 1.96 | 1.15 |
| Item 3 (P1_3) | 0.90 | 1.86 | 1.12 |
| Item 4 (P1_4) | 0.79 | 1.69 | 1.11 |
| Item 5 (P1_5) | 0.78 | 1.73 | 1.09 |
| *Note*. Standardized item loadings for a single factor. | | | |

**Need for chaos (Petersen et al, 2020)**

Confirmatory factor analysis

Petersen, Osmundsen & Arceneaux (2018) identified a two-factor structure based on an EFA for the ‘need for chaos’ measure. Based on the item loadings, cross loadings, and item clarity, we retained eight of the original items and used these to form our new unidimensional. These were items 1, 3, 4, 5, 6, 9, 10 and 11. Based on the CFA, we found that item 4, item 6 and item 10 had high MIs with several other items and were thus, removed from further analysis. The final CFA model resulted in an excellent fit (FIML, χ2(5, N = 5114) = 125.657, p <0.001, RMSEA = 0.069; SRMR = 0.023; CFI = 0.980; TLI = 0.960). The Cronbach’s alpha was 0.88.

| Table U. Standardized loadings based on confirmatory factor loadings | | | |
| --- | --- | --- | --- |
| Item | Factor loading | Mean | SD |
| Item 1 (Q1_1) | 0.82 | 1.98 | 1.57 |
| Item 3 (Q1_3) | 0.85 | 2.04 | 1.61 |
| Item 5 (Q1_5) | 0.75 | 2.36 | 1.72 |
| Item 9 (Q1_9) | 0.82 | 2.17 | 1.63 |
| Item 11 (Q1_11) | 0.64 | 2.65 | 1.74 |
| *Note*. Standardized item loadings for a single factor. | | | |

**Lifestyle and Economic/Government Liberty (Iyer et al, 2012)**

Exploratory factor analysis

Iyer, Koleva, Graham, Ditto & Haidt (2012) identified a two-factor model based on PCA for a nine-item measure. However, two of these items had a different rating scaling and were removed from data collection to avoid confusion for the participants. Thus, we used the seven remaining items to measure libertarian views. Since the factorial structure of the seven-item measure has not been analysed before, we decided to conduct an EFA to evaluate dimensionality.

The overall KMO was 0.78. At an item level, the lowest KMO was 0.73 and the highest KMO was 0.91. Parallel analysis indicated three factors above the eigenvalues derived from random or simulated data (Figure S9). Thus, we conducted a two- and three-factor EFA on a sample of 2557 participants. A three factor EFA showed that item 2 had cross loadings and item 4 had loadings less than 0.2. Moreover, there were only two items loaded onto factor 2 and 3. Thus, a three-factor solution does not appear to be a reasonable factorial structure for this measure. Next, we conducted a two-factor EFA but only item 4 loaded onto the second factor. Thus, we conducted a single factor EFA which showed a clear and interpretable factorial structure. Item 4 had loadings less than 0.3 and hence, was removed from subsequent analysis. The one-factor EFA solution accounted for 34% of the variance and was retained as the final solution. The table below shows the factor loadings of the final one-factor EFA model.

Figure S9. Parallel Analysis


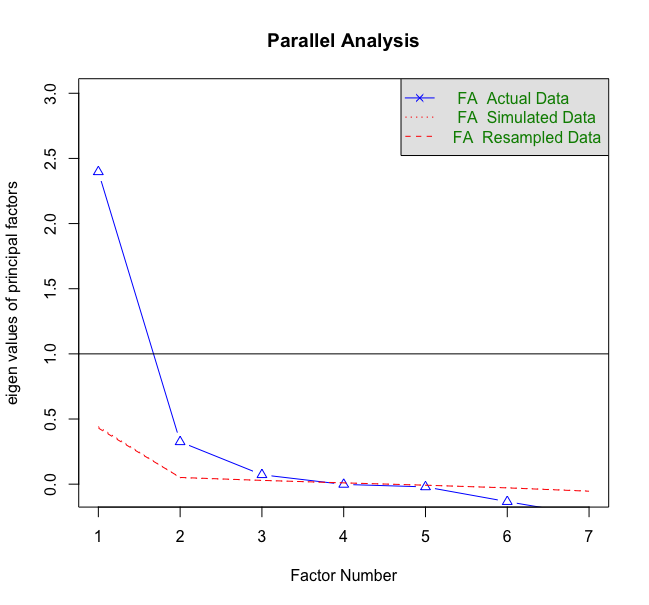


| Table V. Standardized loadings based on exploratory factor loadings | |
| --- | --- |
| Item | Factor loading |
| Item 1 (R1_1) | 0.53 |
| Item 2 (R1_2) | 0.68 |
| Item 3 (R1_3) | 0.53 |
| Item 5 (R1_5) | 0.62 |
| Item 6 (R1_6) | 0.73 |
| Item 7 (R1_7) | 0.65 |
| *Note*. Standardized item loadings for a single factor. | |

Confirmatory factor analysis

We conducted a one-factor CFA and found that item 1 and item 3 were found to have high MIs with several items and were removed from the analysis. The final model with 4 items indicated a good model fit (FIML, χ2(2, N = 2557) = 20.571, p <0.001, RMSEA = 0.060; SRMR = 0.020; CFI = 0.988; TLI = 0.964). The Cronbach’s alpha was 0.76.

| Table W. Standardized loadings based on confirmatory factor loadings | | | |
| --- | --- | --- | --- |
| Item | Factor loading | Mean | SD |
| Item 2 (R1_2) | 0.49 | 4.03 | 1.27 |
| Item 5 (R1_5) | 0.82 | 3.61 | 1.51 |
| Item 6 (R1_6) | 0.69 | 3.86 | 1.41 |
| Item 7 (R1_7) | 0.88 | 4.22 | 1.28 |
| *Note*. Standardized item loadings for a single factor. | | | |

**Populist attitudes (Akkerman et al, 2014)**

Exploratory factor analysis

We used the populist measure described by Akkerman, Mudde & Zaslove (2014) to measure populism. Instead of using all 8 items, we selected 5 which is consistent to what was used in a previous British Election study (2019-2024). Thus, the items used this study were 1,2,4,5 and 7. Since the factorial structure of the 5-item measure has not been analysed before, we decided to conduct an EFA to evaluate dimensionality.

The overall KMO was 0.79. At an item level, the lowest KMO was 0.77 and the highest KMO was 0.83. Parallel analysis indicated three factors above the eigenvalues derived from random or simulated data (Figure S10). Thus, we conducted a two- and three-factor EFA on a sample of 2557 participants. A three factor EFA showed that only two items loaded the first factor and one item loaded onto the third factor. Three items loaded onto the second factor. Thus, a three-factor solution does not appear to be a reasonable factorial structure for this measure. Next, we conducted a two-factor EFA but a Heywood case was detected and thus the estimated were deemed unreliable (Wothke, 1993). Therefore, we conducted a single factor EFA which showed a clear and interpretable factorial structure. All factor loadings were greater than 0.5 and the one-factor solution accounted for 51% of the variance. The table below shows the factor loadings of the final one-factor EFA model.

Figure S10. Parallel Analysis


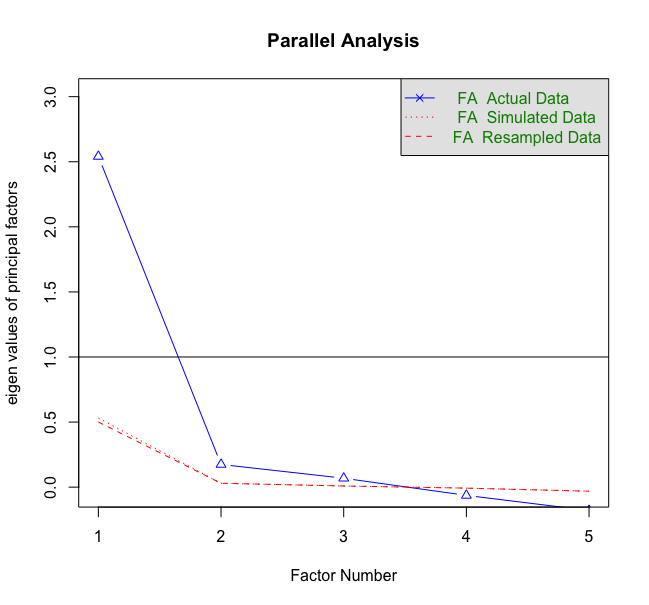


| Table X. Standardized loadings based on exploratory factor loadings | |
| --- | --- |
| Item | Factor loading |
| Item 1 (R1_8) | 0.56 |
| Item 2 (R1_9) | 0.74 |
| Item 4 (R1_10) | 0.75 |
| Item 5 (R1_11) | 0.76 |
| Item 7 (R1_12) | 0.74 |
| *Note*. Standardized item loadings for a single factor. | |

Confirmatory factor analysis

We conducted a one-factor CFA and found that item 2 had very high MIs with several items and was subsequently removed from the analysis.

The final CFA model indicated an excellent model fit (FIML, χ2(2, N = 2557) = 6.951, p <0.05, RMSEA = 0.031; SRMR = 0.01; CFI = 0.998; TLI = 0.993). The Cronbach’s alpha was 0.81.

| Table Y. Standardized loadings based on confirmatory factor loadings | | | |
| --- | --- | --- | --- |
| Item | Factor loading | Mean | SD |
| Item 1 (R1_8) | 0.61 | 4.47 | 1.23 |
| Item 4 (R1_10) | 0.71 | 3.79 | 1.39 |
| Item 5 (R1_11) | 0.80 | 4.36 | 1.3 |
| Item 7 (R1_12) | 0.77 | 3.92 | 1.31 |
| *Note*. Standardized item loadings for a single factor. | | | |

**Very Short Authoritarian Scale**

Confirmatory factor analysis

We used the very short authoritarian scale described by Bizumic, B., & Duckitt, J. (2018) to measure authoritarian. Since a CFA was previously conducted, an EFA was not required. Therefore, we only conducted a CFA to inspect the factor loadings and model goodness-of-fit. However, the CFA showed that four of the five factor loadings were less than 0.4 and the overall model fit was poor. Thus, we decided not to include this scale for further analysis.

**Perceived religious influence on health behaviour and Illness as punishment by God for sin (Holt et al., 2009)**

Exploratory factor analysis

Holt et al. (2009) developed two scales to assess Potential Religion-Health Mechanisms in an African American population. These scales measured ‘perceived religious influence on health behaviour’ and ‘illness as punishment for sin’. Since an EFA had not be done previously, we decided to conduct two separate EFAs to evaluate the scales’ dimensionality.

*Perceived religious influence on health behaviour*

Only seven of the ten items were used for this study. The overall KMO was 0.94. At an item level, the lowest KMO was 0.92 and the highest KMO was 0.96. Parallel analysis indicated three factors above the eigenvalues derived from random or simulated data (Figure S11). Thus, we conducted a two- and three-factor EFA on a sample of 2557 participants. A three factor EFA showed all items loaded onto two factors, with one item on factor two and six items on factor one. Thus, a three-factor solution does not appear to be a reasonable factorial structure for this measure. Next, we conducted a two-factor EFA but all items loaded onto the first factor. Thus, we conducted a single factor EFA which showed a clear and interpretable factorial structure. All loadings were greater than 0.70. The one-factor EFA solution accounted for 74% of the variance and was retained as the final solution. The table below shows the factor loadings of the final two-factor EFA model.

Figure S11. Parallel Analysis


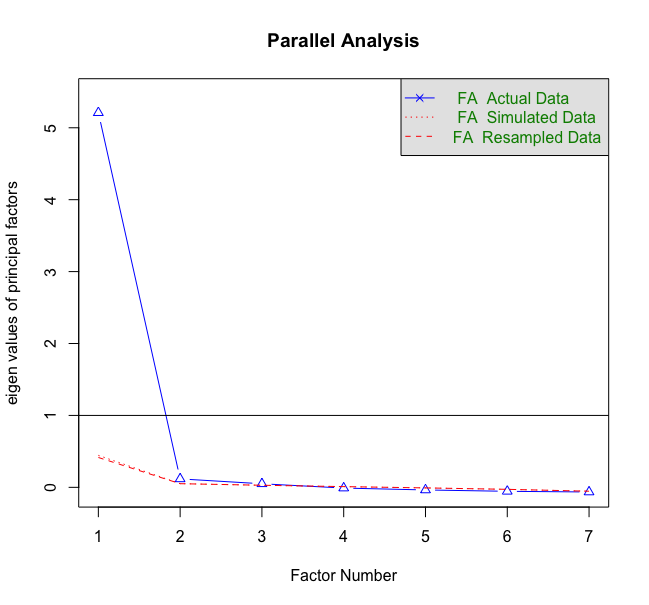


| Table Z. Standardized loadings based on exploratory factor loadings | |
| --- | --- |
| Item | Factor loading |
| Item 1 (S1_1) | 0.79 |
| Item 2 (S1_2) | 0.86 |
| Item 3 (S1_3) | 0.89 |
| Item 4 (S1_4) | 0.90 |
| Item 5 (S1_5) | 0.89 |
| Item 6 (S1_6) | 0.87 |
| Item 7 (S1_7) | 0.83 |
| *Note*. Standardized item loadings for a single factor. | |

*Illness as punishment for sin*

All eight items were used for this study. The overall KMO was 0.96. At an item level, the lowest KMO was 0.96 and the highest KMO was 0.97. Parallel analysis indicated one factor above the eigenvalues derived from random or simulated data (Figure S12). Thus, we conducted a one factor EFA on a sample of 2557 participants. A single factor EFA showed a clear and interpretable factorial structure. All loadings were greater than 0.70. The one-factor EFA solution accounted for 74% of the variance and was retained as the final solution. The table below shows the factor loadings of the final two-factor EFA model.

Figure S12. Parallel Analysis


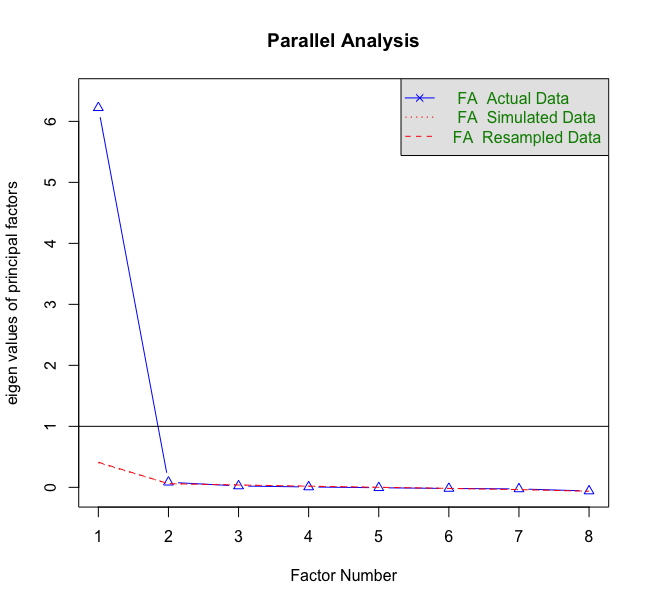


| Table AA. Standardized loadings based on exploratory factor loadings | |
| --- | --- |
| Item | Factor loading |
| Item 1 (S1_8) | 0.77 |
| Item 2 (S1_9) | 0.88 |
| Item 3 (S1_10) | 0.88 |
| Item 4 (S1_11) | 0.91 |
| Item 5 (S1_12) | 0.92 |
| Item 6 (S1_13) | 0.91 |
| Item 7 (S1_14) | 0.86 |
| Item 8 (S1_15) | 0.91 |
| *Note*. Standardized item loadings for a single factor. | |

**Confirmatory factor analysis**

*Perceived religious influence on health behaviour*

We conducted a one-factor CFA analysis and found that item 4 was found to have high MIs with several items and was removed from the analysis. The final model indicated an excellent model fit (FIML, χ2(9, N = 2557) = 90.212, p <0.001, RMSEA = 0.059; SRMR = 0.017; CFI = 0.984; TLI = 0.973). The Cronbach’s alpha was 0.94.

| Table AB. Standardized loadings based on confirmatory factor loadings | | | |
| --- | --- | --- | --- |
| Item | Factor loading | Mean | SD |
| Item 1 (S1_1) | 0.78 | 2.02 | 0.97 |
| Item 2 (S1_2) | 0.87 | 1.91 | 0.96 |
| Item 3 (S1_3) | 0.85 | 1.92 | 0.98 |
| Item 5 (S1_5) | 0.88 | 1.93 | 1.01 |
| Item 6 (S1_6) | 0.89 | 1.97 | 0.99 |
| Item 7 (S1_7) | 0.85 | 1.87 | 0.98 |
| *Note*. Standardized item loadings for a single factor. | | | |

*Illness as punishment for sin*

A one-factor CFA indicated an excellent model fit (FIML, χ2(20, N = 2557) = 142.548, p <0.001, RMSEA = 0.049; SRMR = 0.019; CFI = 0.982; TLI = 0.974). The Cronbach’s alpha was 0.97.

| Table AC. Standardized loadings based on confirmatory factor loadings | | | |
| --- | --- | --- | --- |
| Item | Factor loading | Mean | SD |
| Item 1 (S1_8) | 0.77 | 1.80 | 0.91 |
| Item 2 (S1_9) | 0.87 | 1.73 | 0.93 |
| Item 3 (S1_10) | 0.88 | 1.67 | 0.88 |
| Item 4 (S1_11) | 0.91 | 1.66 | 0.89 |
| Item 5 (S1_12) | 0.92 | 1.64 | 0.87 |
| Item 6 (S1_13) | 0.92 | 1.64 | 0.89 |
| Item 7 (S1_14) | 0.87 | 1.70 | 0.90 |
| Item 8 (S1_15) | 0.90 | 1.63 | 0.88 |
| *Note*. Standardized item loadings for a single factor. | | | |

**Multiple regression with all the socio-demographic variables**

Regression estimates:

Dummy coded: Ethnicity (white), Relationship status (married), Region (SE), Housing-situation (rented council), Employment before covid (unemployed), Has employment during covid (No). Variables in brackets are the ones used as reference group.

First column (Estimates) shows the unstandardised regression coefficient

Last column (Std.all) shows the standardised regression coefficient


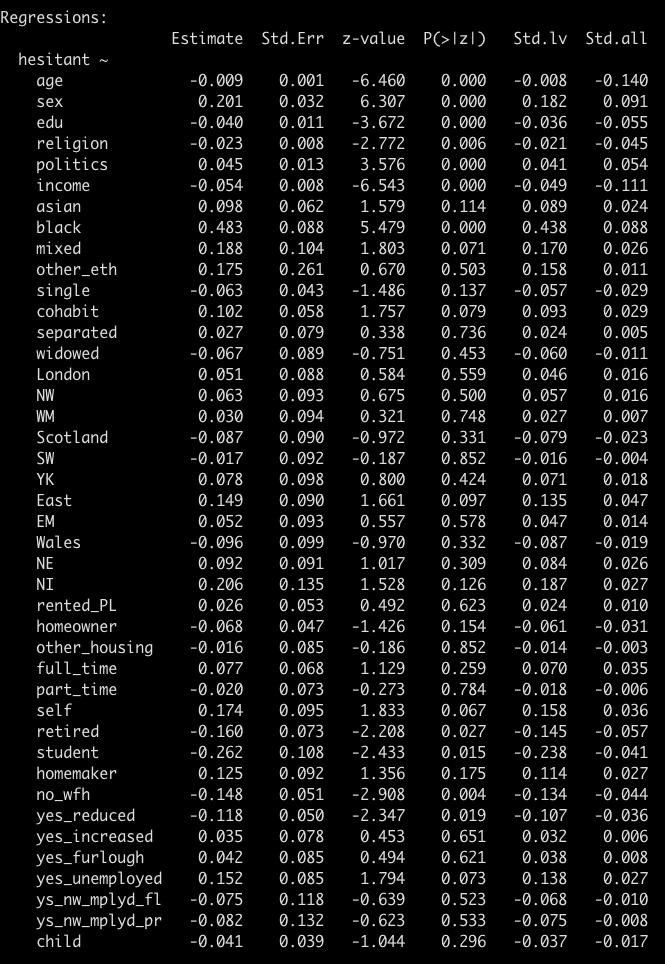


R-square

See last row: hesitant: 0.098 (10% variance explained)


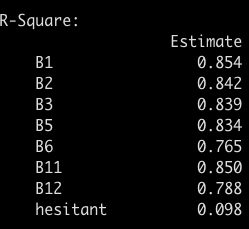


**References for supplementary materials**

Retrieved from https://www.britishelectionstudy.com/wp-content/uploads/2019/06/Bes_wave15Documentation_V2.pdf

Afifi, A., May, S., & Clark, V. A. (2003). Computer-aided multivariate analysis. Boca Raton, Florida: CRC Press.

Akaike, H. (1987). Factor analysis and AIC. In Selected papers of hirotugu akaike (pp. 371-386). Springer, New York, NY.

Akkerman A, Mudde C, Zaslove A. (2014) How Populist Are the People? Measuring Populist Attitudes in Voters. Comparative Political Studies. ;47(9):1324-1353. doi:10.1177/0010414013512600

Bizumic, B., & Duckitt, J. (2018). Investigating right wing authoritarianism with a very short authoritarianism scale.

Domek, G. J., O'Leary, S. T., Bull, S., Bronsert, M., Contreras-Roldan, I. L., Ventura, G. A. B., ... & Asturias, E. J. (2018). Measuring vaccine hesitancy: Field testing the WHO SAGE Working Group on Vaccine Hesitancy survey tool in Guatemala. Vaccine, 36(35), 5273-5281.

Enders, C. K. (2001). The impact of nonnormality on full information maximum-likelihood estimation for structural equation models with missing data. Psychological methods, 6, 352.

Enders, C. K. & Bandalos, D. L. (2001). The relative performance of full information maximum likelihood estimation for missing data in structural equation models. Structural equation modeling, 8, 430-457.

Forbes D, Alkemade N, Mitchell D, Elhai JD, McHugh T, Bates G, Novaco RW, Bryant R, Lewis V. (2014) Utility of the Dimensions of Anger Reactions-5 (DAR-5) scale as a brief anger measure. Depress Anxiety. Feb;31(2):166-73. doi: 10.1002/da.22148. Epub 2013 Jun 25. PMID: 23801571.

Fowler, D., Freeman, D., Smith, B., Kuipers, E., Bebbington, P., Bashforth, H., . . . Garety, P. (2006). The Brief Core Schema Scales (BCSS): Psychometric properties and associations with paranoia and grandiosity in non-clinical and psychosis samples. Psychological Medicine, 36(6), 749-759. doi:10.1017/S0033291706007355

Freeman, D., Waite, F., Rosebrock, L., Petit, A., Causier, C., East, A., Jenner, L., Teale, A., Carr, L., Mulhall, S., Bold, E., & Lambe, S. (2020). Coronavirus conspiracy beliefs, mistrust, and compliance with government guidelines in England. Psychological Medicine https://doi.org/10.1017/S0033291720001890

Gerbing, D. W., & Anderson, J. C. (1984). On the meaning of within-factor correlated measurement errors. Journal of Consumer Research, 11(1), 572-580.

Grewal, R., Cote, J. A., & Baumgartner, H. (2004). Multicollinearity and measurement error in structural equation models: Implications for theory testing. Marketing science, 23, 519-529.

Harman, H. H., & Jones, W. H. (1966). Factor analysis by minimizing residuals (minres). *Psychometrika*, *31*, 351-368.

Hayes, T., & Usami, S. (2020). Factor score regression in the presence of correlated unique factors. *Educational and Psychological Measurement*, *80*(1), 5-40.

Hocking, R. R. (1976). A Biometrics invited paper. The analysis and selection of variables in linear regression. Biometrics, 32(1), 1-49.

Holt, C. L., Clark, E. M., Roth, D., Crowther, M., Kohler, C., Fouad, M., ... & Southward, P. L. (2009). Development and validation of instruments to assess potential religion-health mechanisms in an African American population. *Journal of Black Psychology*, *35*(2), 271-288.

Hu, L., & Bentler, P. M. (1999). Cutoff criteria for fit indexes in covariance structure analysis: Conventional criteria versus new alternatives. Structural Equation Modeling, 6, 1–55.

Iyer, R., Koleva, S., Graham, J., Ditto, P., & Haidt, J. (2012). Understanding libertarian morality: The psychological dispositions of self-identified libertarians. *PloS one*, *7*(8), e42366.

Kaiser, H. F. (1970). A second generation little jiffy. Psychometrika, 35, 401-415.

Kiers, H. A., & Smilde, A. K. (2007). A comparison of various methods for multivariate regression with highly collinear variables. Statistical Methods and Applications, 16, 193–228.

Kline, R. B. (2015). Principles and practice of structural equation modeling. New York: Guilford publications.

Kolenikov, S., & Bollen, K. A. (2012). Testing negative error variances: Is a Heywood case a symptom of misspecification?. Sociological Methods & Research, 41(1), 124-167.

Knowlden, A. P. (2014). Role of Suppressor Variables in Primary Prevention Obesity Research: Examples from Two Predictive Models. International Scholarly Research Notices, Volume 2014, Article ID 567523, https://doi.org/10.1155/2014/567523.

Lancaster, B. P. (1999). Defining and Interpreting Suppressor Effects: Advantages and Limitations. Paper presented at the annual meeting of the Southwest Educational Research Association, San Antonio, TX.

Lin, L. C., Huang, P. H., & Weng, L. J. (2017). Selecting path models in SEM: A comparison of model selection criteria. Structural Equation Modeling: A Multidisciplinary Journal, 24, 855-869.

Little, R. J., & Rubin, D. B. (2019). Statistical analysis with missing data (Vol. 793). New Jersey: John Wiley & Sons.

Marsh, H. W., Hau, K. T., Balla, J. R., & Grayson, D. (1998). Is more ever too much? The number of indicators per factor in confirmatory factor analysis. *Multivariate Behavioral Research,* **33**(2), 181–220.

Marteau, T. M. (1990). Attitudes to doctors and medicine: The preliminary development of a new scale. Psychology and Health, 4, 351–356

Petersen, M. B., Osmundsen, M., & Arceneaux, K. (2018). A “need for chaos” and the sharing of hostile political rumors in advanced democracies.

Paulhus, D. L., Robins, R. W., Trzesniewski, K. H., & Tracy, J. L. (2004). Two replicable suppressor situations in personality research. Multivariate Behavioral Research, 39, 303-328.

Revelle, W. (2014). psych: Procedures for psychological, psychometric, and personality research. *Northwestern University, Evanston, Illinois*, *165*, 1-10.

Rosseel, Y. (2012). Lavaan: An R package for structural equation modeling and more. Version 0.5–12 (BETA). *Journal of statistical software*, *48*(2), 1-36.

Rubin, D. B. (1976). Inference and missing data. Biometrika, 63, 581-592.

Schwarz, G. (1978). Estimating the dimension of a model. The annals of statistics, 6, 461-464.

Shapiro, G. K., Holding, A., Perez, S., Amsel, R., & Rosberger, Z. (2016). Validation of the vaccine conspiracy beliefs scale. Papillomavirus research, 2, 167-172.

Shapiro GK, Tatar O, Dube E, et al. (2018) The vaccine hesitancy scale: Psychometric properties and validation. Vaccine.; 36(5):660-667. doi:10.1016/j.vaccine.2017.12.043

Tellegen, A., & Waller, N. G. (2008). Exploring personality through test construction: Development of the Multidimensional Personality Questionnaire. In G. J. Boyle, G. Matthews & D. H. Saklofske (Eds.), *The SAGE handbook of personality theory and assessment* (Vol. 2, pp. 261-292). Thousand Oaks, CA: Sage.

Vrieze, S. I. (2012). Model selection and psychological theory: a discussion of the differences between the Akaike information criterion (AIC) and the Bayesian information criterion (BIC). Psychological methods, 17, 228-243.

Walker, D. A. (2003). Suppressor variable (s) importance within a regression model: an example of salary compression from career services. Journal of College Student Development, 44, 127-133.

Watson, D., Clark, L. A., Chmielewski, M., & Kotov, R. (2013). The value of suppressor effects in explicating the construct validity of symptom measures. Psychological assessment, 25, 929-941.

Williams, B., Onsman, A., & Brown, T. (2010). Exploratory factor analysis: A five-step guide for novices. *Australasian Journal of Paramedicine*, *8*, 93. doi:10.33151/ajp.8.3.93

Wothke, W. (1993). Nonpositive definite matrices in structural modeling. *Sage Focus Editions*, *154*, 256-256.
